# Single step syntheses of (1*S*)-aryl-tetrahydroisoquinolines by norcoclaurine synthases

**DOI:** 10.1038/s42004-020-00416-8

**Published:** 2020-11-13

**Authors:** Rebecca Roddan, Altin Sula, Daniel Méndez-Sánchez, Fabiana Subrizi, Benjamin R. Lichman, Joseph Broomfield, Michael Richter, Jennifer N. Andexer, John M. Ward, Nicholas H. Keep, Helen C. Hailes

**Affiliations:** 1 Department of Biological Sciences, Institute of Structural and Molecular Biology, Birkbeck College, London, WC1E 7HX UK; 2 Department of Chemistry, Christopher Ingold Building, University College London, London, WC1H 0AJ UK; 3 Department of Biochemical Engineering, Bernard Katz Building, University College London, London, WC1E 6BT UK; 4 Fraunhofer Institute for Interfacial Engineering and Biotechnology IGB, Schulgasse 11a, 94315 Straubing, Germany; 5 Institute of Pharmaceutical Sciences, University of Freiburg, Albertstrasse 25, 79104 Freiburg, Germany; 6 Present Address: Centre for Novel Agricultural Products, Department of Biology, University of York, York, YO10 5DD UK

**Keywords:** X-ray crystallography, Biocatalysis

## Abstract

The 1-aryl-tetrahydroisoquinoline (1-aryl-THIQ) moiety is found in many biologically active molecules. Single enantiomer chemical syntheses are challenging and although some biocatalytic routes have been reported, the substrate scope is limited to certain structural motifs. The enzyme norcoclaurine synthase (NCS), involved in plant alkaloid biosynthesis, has been shown to perform stereoselective Pictet–Spengler reactions between dopamine and several carbonyl substrates. Here, benzaldehydes are explored as substrates and found to be accepted by both wild-type and mutant constructs of NCS. In particular, the variant M97V gives a range of (1 *S*)-aryl-THIQs in high yields (48–99%) and e.e.s (79–95%). A co-crystallised structure of the M97V variant with an active site reaction intermediate analogue is also obtained with the ligand in a pre-cyclisation conformation, consistent with (1 *S*)-THIQs formation. Selected THIQs are then used with catechol *O*-methyltransferases with exceptional regioselectivity. This work demonstrates valuable biocatalytic approaches to a range of (1 *S*)-THIQs.

## Abstract (web summary)

The 1-aryl-tetrahydroisoquinoline moiety is a desirable synthetic target, but generating single enantiomers of THIQ products is synthetically challenging. Here the authors demonstrate that the M97V variant of enzyme norcoclaurine synthase catalyzes the synthesis of (1 *S*)-aryl-THIQs in high yields and enantiomeric excesses.

## Introduction

**Fig. 1:**
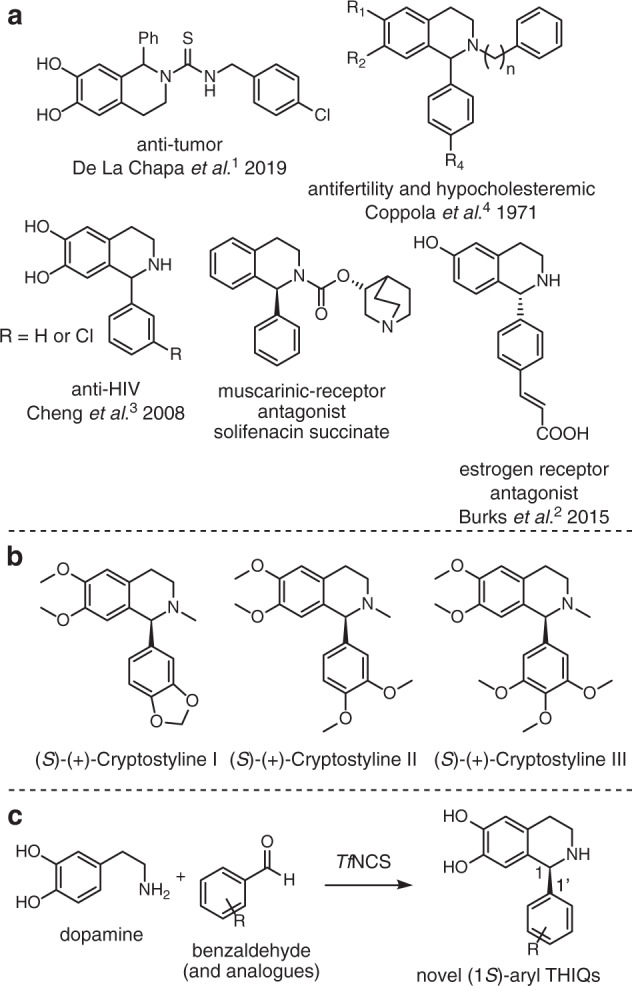
Pharmaceutically relevant 1-aryl-THIQs, naturally occurring 1-aryl-THIQs and a proposed route to these compounds using *Tf*NCS (NCS isolated from *Thalictrum flavum*). A range of **a** pharmaceutically relevant 1-aryl-THIQs and **b** the Cryptostylines, a variety of naturally occurring enantiopure 1-aryl-THIQs isolated from *C. fulva*. The analogous *levo*rotatory compounds were isolated from *C. erythroglossa*^20,22^. **c** The generation of a variety of (1 *S*)-aryl THIQ products in a single, regioselective and enantioselective step, using *Tf*NCS as a reaction catalyst (WT or single-point variant).

The tetrahydroisoquinoline (THIQ) moiety is present in many biologically active molecules and so is a desirable synthetic target for pharmaceutical research. Generating single enantiomer and single regioisomer THIQ products is synthetically challenging. The 1-aryl THIQ moiety is found in many synthetic biologically active small molecules (examples shown in Fig. 1a), which have a variety of medicinal benefits, including anti-tumour^1,2^, anti-HIV^3^ and contraceptive^4^ activities and natural products have also been isolated (Fig. 1b). Solifenacin succinate is a widely prescribed muscarinic receptor antagonist, used for the treatment of an overactive bladder with ~3 million US prescriptions per year^5^.

Routes to racemic 1-aryl-THIQs are currently via Pictet–Spengler reactions (acid or phosphate catalysed)^6,7^ or via a three-step synthesis, involving amide formation, a Bischler–Napieralski cyclisation to give the imine, followed by reduction to give the final THIQ scaffold^8^. However, these approaches are limited by poor reactivity and stereoselectivity (Pictet–Spengler reactions) or the use of forcing conditions (via the Bischler–Napieralski reaction).

Enantioselective syntheses of small molecules are meaningful because each enantiomer can possess different biological activities. Synthetic routes involve a chiral inductor^9^ or most commonly, a stereoselective reduction of the imine using an inorganic catalyst (often iridium-based)^10–12^. These catalysts can, however have narrow substrate scopes. In another synthetic route developed by Chen et al.^13^, the asymmetric hydrogenation of the heterocyclic ring of a variety of isoquinolines was performed using a chiral iridium catalyst, giving a range of (1 *R*)-THIQ products in high yields (>80%) and high enantiomeric excesses (e.e.s.; 90–99%). However, hydrogenation must be performed under high pressure (ca. 80 bar), the isoquinolines must be first prepared synthetically, and there are sustainability issues associated with the use of iridium^14,15^.

Some enzymatic routes have been developed to generate single enantiomer 1-aryl-THIQs. Routes using imine reductases (IREDs), from the corresponding dihydroisoquinoline, have involved the directed evolution of artificial metalloenzymes based upon biotin–streptavidin binding^16^ or the screening of large catalogues of IREDs^17,18^. However, there is a synthetic requirement to generate the starting material and harsh reaction conditions are involved. Another route to these compounds involves the use of an enantiospecific monoamine oxidase (MAO), developed using directed evolution methods^19^. The MAO selectively oxidises a single enantiomer of a racemic THIQ to give the imine, which is reduced in situ, resulting in the accumulation of the opposing single enantiomer of amine to the one that the MAO oxidises. Single enantiomers of a range 1-aryl-THIQs can be generated using these methods in high yields and e.e.s.; however, starting materials must be synthesised chemically and oxidatively sensitive groups (such as hydroxyls) are not tolerated on the bicyclic ring system, although this moiety is found in biologically active compounds (Fig. 1a).

The majority of examples of naturally occurring THIQs are benzylic at the C-1 position, generated in the NCS-mediated pathway to benzylisoquinoline alkaloids. A variety of enantiopure 1-aryl-THIQs, known as the cryptostylines have been isolated from two species of orchid in the 1970s (as shown in Fig. 1b). Interestingly, in *Cryptostylis erythroglossa*, solely the *R*-enantiomer of product was isolated and in *Cryptostylis fulva*, only the *S*-enantiomer was found (Fig. 1b)^20,21^. The stereochemistry of the isolated products was determined by x-ray crystallography^22^. Some isotope feeding studies were performed and determined that the amine required was dopamine, generated from tyrosine or phenylalanine in an analogous pathway to the biosynthetic routes in BIA-producing plants. The biosynthetic origin of the phenyl group at C-1 is unknown^21^. Due to the apparent isolation of single isomer products, it is likely that the biosynthesis involves an epimerase or Pictet–Spenglerase.

Work recently published by our group^23^, showed that despite previous reports, α-methyl-substituted aldehydes are well-tolerated as substrates for *Tf*NCS. When a racemic aldehyde was used as the carbonyl substrate, a kinetic resolution of the aldehyde was performed by the wild-type (WT) enzyme, giving THIQ products with (1 *S*,1′ *R*) stereochemistry in high diastereomeric ratios (d.r.s.; up to 96:4). Two active site mutants, L76V and M97V were shown to improve d.r.s. (up to 98:2) and conversions in products (up to >99%), respectively^23^. *Tf*NCS has also been shown to accept a variety of bulky ketones as substrates, to generate 1,1-disubstituted THIQs^24^. Therefore, it is not implausible that benzaldehydes may also be accepted as substrates.

Previous reports have shown that benzaldehyde derivatives were not well-tolerated as substrates by NCSs. Screening showed that only trace amounts of product were generated using benzaldehyde or 3-hydroxybenzaldehyde as substrates using WT*-Tf*NCS or *Cj*NCS2 (isolated from *Coptis japonica*)^25,26^. In both cases, enzymatic assays were performed with low substrate loading (10 μM–1 mM) and some of the product could have possibly been generated by non-enzymatic reactions. Subsequent studies have reported high *K*_d_ values for both amine and aldehyde substrates, indicating that high substrate loading is necessary for increased conversions^27^. Indeed, attempts to generate unnatural THIQ products using alpha-substituted amino acids (L-valine and L-isoleucine) in vivo, using a yeast-based platform have been unsuccessful, probably as high substrate loading is challenging to achieve in these systems^28^. In this work, we hypothesise that by modification of the reaction conditions, such as enzyme or substrate loading, and using WT and variants of *Tf*NCS together with benzaldehydes, the portfolio of asymmetric THIQs that can be synthesised via Pictet–Spenglerases can be significantly expanded. This can also lead to the first single-step syntheses of a range of (1 *S*)-1-aryl-THIQs which are challenging to synthesise chemically due to the oxidatively sensitive catechol (Fig. 1c).

## Results and discussion

### Expanding the NCS substrate scope

**Fig. 2:**
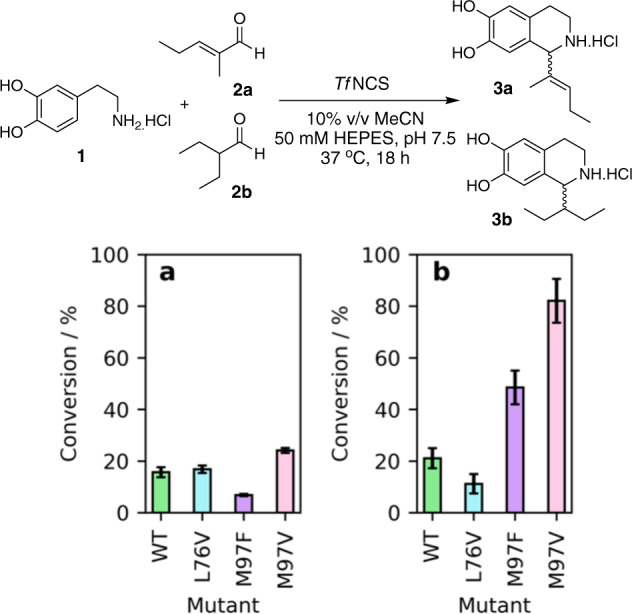
HPLC conversions (determined against product standards) of reactions between dopamine and 2-methyl-2-pentenal or 2-ethylbutanal. Reaction between dopamine (**1**) and **a** 2-methyl-2-pentenal (**2a**) to give (**3a**) or **b** 2-ethylbutanal (**2b**) to give **3b**. *Tf*NCSs were used as reaction catalysts at 0.2 mg mL^−1^ concentration. Reactions were performed in triplicate and standard deviations reported.

Previous work in our group has shown that α-methyl-substituted aldehydes are accepted as NCS substrates, despite earlier reports indicating little or no acceptance^25,29^. From this, initial test screens were performed using WT-*Tf*NCS and variants (L76V, M97F and M97V), with **1**, and the substrates 2-methyl-2-pentenal (**2a**) and 2-ethylbutanal (**2b**), where low conversions were previously observed with the WT enzyme (Fig. 2)^23^. These were selected as representative of larger disubstituted groups at the alpha-position and an *sp*^2^-carbon as is the case in benzaldehydes. There is interest in accessing the THIQ product (**3b**) as the racemic compound has been shown to act as a bronchodilator^30,31^.

For enzymatic reactions between dopamine (**1**) and (**2a**), conversions were similar when using the mutant and the WT constructs. Based on a co-crystallised structure of NCS gained with an α-methyl-substituted reaction mimic (PDB: 6RP3), the aldehyde side chain (i.e., here -C(CH_3_)( = CHC_2_H_5_) is likely to be proximal to the L76V region in the active site^23^. However, due to the lack of rotational freedom of this, a small increase in space may not be sufficient to improve conversions and conjugation of the alkene with the aldehyde results in poor activation of the aldehyde. For conversions with 2-ethylbutanal (**2b**) as the substrate, a fourfold improvement was observed with M97V (and also interestingly by M97F), further highlighting its applicability with α-substituted aldehydes as substrates. These initial screens indicated that unsaturated aldehydes and more sterically challenging α-disubstituted aldehydes could be accepted by NCS variants.

### Acceptance of benzaldehyde and derivatives by NCS

**Fig. 3:**
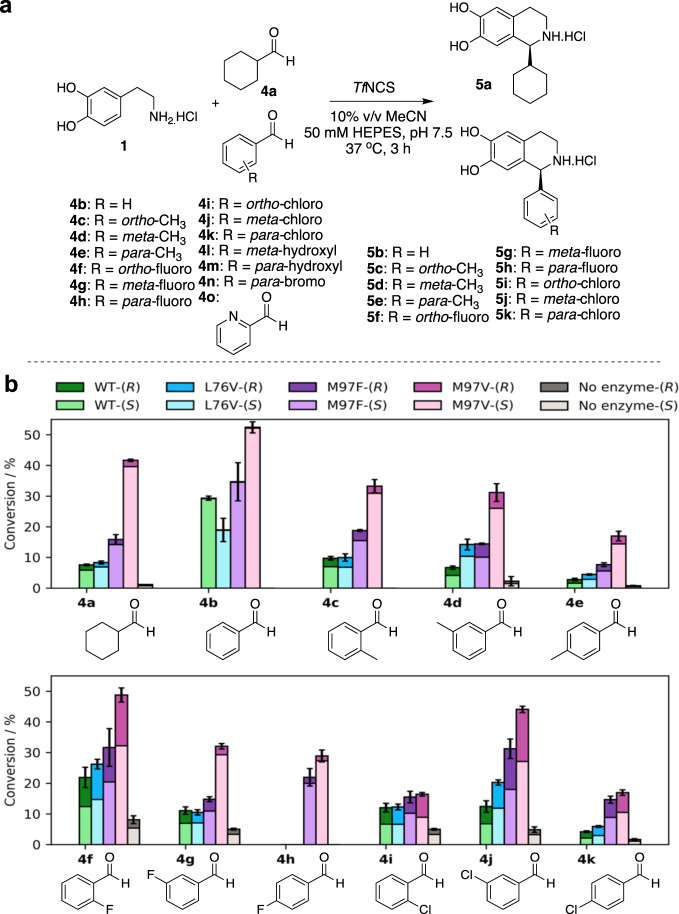
Initial *Tf*NCS-catalysed reactions between dopamine and aldehydes 4a–o. **a** Reactions performed between dopamine (**1**) and aldehydes (**4a**–**k**). Reactions with aldehydes **4l**–**o** were performed using 0.5 mg mL^−1^ final concentration of Δ29*Tf*NCS-M97V. **b** Conversions of reactions between dopamine (**1**) and benzaldehyde derivatives (**4a–k**). WT-*Tf*NCS or active site mutants of *Tf*NCS were used as the reaction catalyst with 0.2 mg mL^−1^ enzyme. Samples were prepared by workup method 1, conversions were determined by monitoring product formation against standards (Supplementary Figs. 24–26 and Supplementary Methods) by analytical achiral HPLC (method 1). Product enantiomeric purities are given by indicating the amounts of *R*- and *S*-product generated and were determined by chiral HPLC analysis. Reactions were performed in triplicate and standard deviations reported.

In previous screens, when dopamine and benzaldehyde were used with *Tf*NCS, products were not detected or trace conversions were observed, possibly due to the lower enzyme or substrate loading used^25^. As demonstrated in previous reports, high substrate loading is well-tolerated by NCS and is indeed required to gain high conversions of the substrate (or high yields of product)^23,24^. The *K*_d_ value of dopamine was determined by NMR titration experiments and found to be 5 mM, thus indicating very weak substrate binding^27^. With this in mind, assays performed in this study used increased substrate loading; 10 mM amine and 20 mM aldehyde. An excess of aldehyde was used as benzaldehydes can be easily oxidised^32^. Assays were performed using benzaldehyde and a variety of analogues with dopamine (**1**) as the amine substrate. Conversions using WT-*Tf*NCS and the mutants L76V, M97F and M97V are given in Fig. 3. Reactions were performed for 3 h to minimise the contribution of the background Pictet–Spengler reaction between the two substrates, as the aldehyde is highly activated^7,33,34^. Racemic standards for chiral HPLC analyses were prepared using a phosphate-mediated Pictet–Spengler reaction (Supplementary Fig. 7 and Supplementary Methods).

Remarkably, all the benzaldehydes screened, possessing both electron-donating and electron-withdrawing groups (**4a–k**), were accepted as substrates by all NCSs, including WT*-Tf*NCS, despite previous reports of no or trace conversions (Fig. 3)^25^. Indeed, on performing the reaction to give **5b** under the conditions described by O’Connor et al. (in their supplementary data) only trace amounts of **5b** were formed (Supplementary Fig. 9). Modification of these conditions, highlighted that both higher enzyme and substrate concentrations enhanced product formation.

High enantiopurity was observed with **4a** (>99% for all *Tf*NCS variants). Conversions with WT-NCS and L76V with aldehydes **4a–k** were similar and conversions with both substitutions at residue 97 resulted in improved yields. This is particularly interesting with the M97F variant as due to the bulky phenyl group, there is less space in this region of the active site^23^. In addition, for reactions with ketones as substrates, mutations at the M97 position (to phenylalanine, leucine or valine) resulted in poorer yields compared with WT-*Tf*NCS. Previous NMR and molecular dynamics studies of WT-*Tf*NCS enzyme, indicated it to be highly dynamic during the catalytic process with substrate binding, resulting in large conformational changes across the entire protein^23,27^. The increased conversions and e.e.s. in products from reactions with M97F and M97V could therefore be due to an effect whereby when faster binding of the substrates occurs (by increasing hydrophobicity or active site space, respectively) and turnover is faster, so the background Pictet–Spengler reaction is less efficient and product enantiopurity is improved.

The effects of electron-donating (**4c–e**) and electron-withdrawing substituents (**4f–k**) on the aldehyde aromatic ring were explored by performing reactions with *ortho*-, *meta*- and *para*-substituted analogues. For reactions with the substituted aldehydes (**4a** and **4c–k**), e.e.s. in the product were decreased compared with benzaldehyde (**4b**), despite often low or no observed background reactions. This suggested that the lower stereoselectivities were due to imine intermediate positioning in the active site. This was most notably observed with the more activated aldehydes, the *ortho*-halogenated benzaldehydes (**4f** and **4i**). The fluorinated benzaldehydes (**4f–h**), which are of a similar size to benzaldehyde, were well-accepted as substrates. *Para*-substitutions on the aryl ring were generally less well-tolerated, potentially due to steric hinderance in the active site, particularly with methyl- and chloro-substituted benzaldehydes (**4e** and **4k**, respectively). Using these reaction conditions, enzymatic reactions towards **5a–k** were performed on a biocatalytic preparative scale (10 mL scale), with products readily isolated and characterised (Supplementary Methods).

With a view to further extending the benzaldehyde substrate scope, further activated halogenated and a heterocyclic derivative (**4l–o**, Fig. 3a) were tested, however, all were poorly accepted. *Para*-substitution has already been shown to be poorly tolerated (**4e**, **h** and **k**, Fig. 3b) and performing reactions with *para-*hydroxylated (**4** **m**) and *para*-brominated (**4n**) aldehydes resulted in trace conversions, probably due to increased steric hindrance in the active site. The *meta*-hydroxylated aldehyde (**4** **l**) was also poorly accepted, likely due to carbonyl deactivation and steric effects. A high background reaction was observed with 2-pyridinecarboxaldehyde (**4o**) as a substrate because the aldehyde is highly activated due to electron-withdrawing effects.

### Optimisation of reaction conditions

**Fig. 4:**
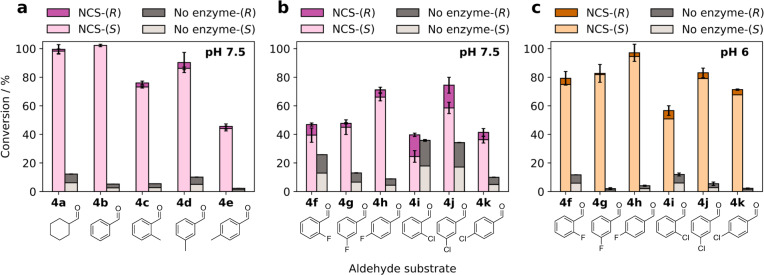
Conversions (determined against product standards) and enantiomeric excesses of products generated by a M97V-Δ29*Tf*NCS-catalysed reaction between dopamine and a variety of aldehyde derivatives. Reactions were performed using 0.5 mg mL^−1^ final concentration of enzyme for 24 h. **a** Reactions between dopamine (**1**) and **4a**-**e** at pH 7.5. **b** Reactions between dopamine (**1**) and **4f**–**k** at pH 7.5. **c** Reactions between dopamine (**1**) and **4f**–**k** at pH 6. Reactions were performed in triplicate and the error bars given are the standard deviations.

It was hoped that reaction optimisation using the most productive variant M97V could lead to higher conversions and e.e.s in the products. Timepoint assays were therefore performed for the reactions between dopamine (**1**) and a representative substrate *meta*-methylbenzaldehyde (**4d**) (Supplementary Fig. 8), using varying concentrations of M97V as the reaction catalyst with the reactions halted at various timepoints. For an NCS-mediated reaction with an enzyme concentration of 0.2 mg mL^−1^ and a reaction time of 3 h (Fig. 3b), the product (**5d**) was obtained in poor enantiopurity (67% e.e.) and low yield (31%). Using higher enzyme concentrations (0.5 mg mL^−1^), a high racemic background reaction was again observed (15% conversion after 24 h). However, the amount of *R*-product formed was higher than would be expected based upon the reactions in the absence of enzyme, suggesting that this is an enzyme-mediated reaction, but high concentrations of enzyme are also required for fast turnover of the substrates to give high e.e.s. For all reactions with **4d**, with 0.2 mg mL^−1^ of enzyme e.e.s of 74–78% were observed, whereas for 0.5 mg mL^−1^ enzyme, e.e.s of 84–85% were observed at 2–6 h, increasing to an e.e. of 87% at 24 h, combined with very high conversion levels (94%). These reaction conditions were then used with the other aldehydes (**4a–k**) (M97V-Δ29*Tf*NCS 0.5 mg mL^−1^, 24 h) and the conversions, with the amounts of *R* and *S* products generated, are given in Fig. 4a, b.

Complete conversions were observed for reactions to give **5a** and **5b** with both products formed in excellent enantiopurities (97% and >99%, respectively, Fig. 4a). These conditions also significantly improved both the conversions and enantiopurity of the products with methylated benzaldehyde derivatives (**4c–e**). Conversions of 76% and 90% were observed for the *ortho*- (**4c**) and *meta*- (**4d**) substituted compounds, respectively, in >90% e.e. Lower conversions were again observed with the *para*-substituted analogue (**4e**), (45% yield) but improved enantiopurities were noted. Despite the observation of a high racemic background reaction for NCS-catalysed reactions involving fluorinated benzaldehyde derivatives, reasonable e.e.s in the products were obtained (68–88%). Higher background reactions were observed for reactions with the chlorinated benzaldehydes (**4i-k**). Interestingly, a similar trend was observed with the halogenated analogues; lower background reactions were observed with the *para* < *meta* < *ortho*. Presumably, this is due to a combination of steric factors and activation of the carbonyl group. Despite this, the *para*-chlorinated product (**5k**) was generated in reasonable yield (41%), and increased enantiopurity (75% vs. 21% e.e., Fig. 4b vs. Fig. 3).

Gaining high e.e.s. in the products for reactions with halogenated benzaldehydes (**5f–k**) still proved challenging. It was hypothesised that this was due to a combination of a high background reaction (as the aldehydes are highly activated) and it was suspected that this could be due to increased lability of the C-1 proton, due to the presence of electron-withdrawing halogen atoms on the aryl ring. In an attempt to minimise in situ racemisation at this position, reactions were performed at a lower pH (6 vs. 7.5). Conversions and enantiomeric ratios of the resulting products (**5f–k**) are given in Fig. 4c.

Remarkably, improved yields and e.e.s. were observed in all cases. In particular, with the *ortho*-chlorinated product (**5i**), at pH 7.5, the yield and e.e. observed were 40% and 19%, respectively, whereas at a lower pH, both were improved to 57% and 79%. This was in part due to the minimal background reaction observed, which was unexpected, considering that the Pictet–Spengler reaction is catalysed by acidic conditions. Previous reports of *Tf*NCS-catalysed reactions are typically performed at near neutral pH (7–7.5)^29,35,36^, so it is interesting that performing reactions at pH 6 improves both the enantiopurity and conversions with these substrates. It is possible that the catecholic side reactions are reduced at this pH. It is however clear that optimising reaction conditions is worthwhile for each substrate type.

### Determination of the absolute stereochemistry of products

To confirm the stereochemistry at the C-1 position, the product **5b** was *O*-methylated as chiral HPLC and optical rotation data has been reported for this analogue^37^. THIQ **5b** was first *N*-Boc protected (Supplementary Fig. 29). Deprotonation of the catechol is required for methylation to occur; however, the C-1 proton of the *N*-Boc protected THIQ is labile due to the neighbouring, electron-withdrawing Boc group. Attempts to perform this step using potassium carbonate as the base were unsuccessful, with racemisation observed (Supplementary Figs. 21 and 22). Therefore, the reaction was repeated, using a bulkier base, caesium carbonate. An e.e of 20% was observed in the resultant product, with the major peak corresponding to the (1 *S*)-isomer (Supplementary Figs. 21 and 22), confirming that the major enantiomer in the 1-aryl-THIQs generated by NCSs was (1 *S*). Full synthetic procedures given in the Supplementary Methods.

### Co-crystallised structure of M97V-*Tf*NCS with a mimic

**Fig. 5:**
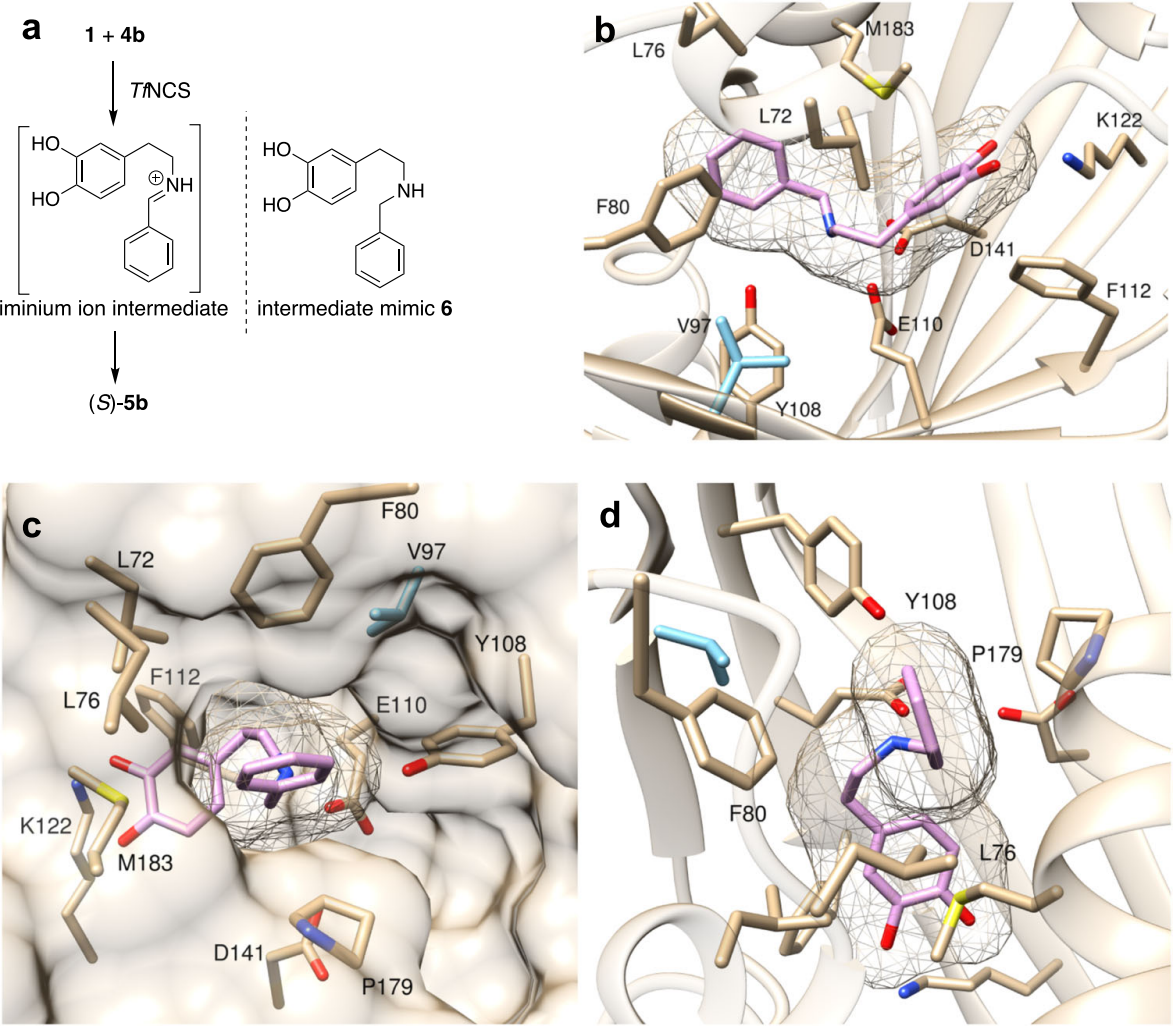
Crystallographic investigations of M97V-*Tf*NCS. **a** Rationale of (**6**) design, a non-productive analogue of the iminium ion intermediate of the NCS-mediated reaction between **1** and **4b**, to give **5b**. **b**–**d** Location of **6** bound in the active site of M97V-Δ33*Tf*NCS (PDB: 6Z82), with the mutation M97V given in blue.

To gain further insight into the NCS mechanistic process, a co-crystallised structure of the most promising single-point variant, M97V, with a non-productive reaction intermediate bound in the active site was obtained. This approach has been successful for gaining mechanistic insights into the WT enzyme^23,38^ and has recently been used to show that when altering the aldehyde substrate of another Pictet–Spenglerase, strictosidine synthase, inverted substrate binding occurred compared with when using the natural substrates^39^. The non-productive secondary amine mimic (**6**) of the iminium ion intermediate of the reaction between **1** and **4b** was synthesised (Supplementary Fig. 30 and Supplementary Methods), and a truncated construct of M97V-*Tf*NCS generated, M97V-Δ33*Tf*NCS, omitting residues (1–33 and 196–210; Supplementary Figs. 1–3 and Supplementary Methods) in the signal peptides at both termini, as with previous studies^23,38^. A co-crystallised structure was gained of M97V-Δ33*Tf*NCS with **6** bound in the active site (Fig. 5) at 2.3 Å with a single copy in the asymmetric unit (PDB: 6Z82; Supplementary Table 1, Supplementary Fig. 23, and Supplementary Methods).

The ligand orientation was consistent with the ‘dopamine-first mechanism’ with mechanistically relevant hydrogen-bonding interactions observed between the catechol moiety of **6** and K122, and the secondary amine of **6** and E110. The ligand was also observed in a pre-cyclisation conformation, which interestingly was consistent with the stereochemical outcome of the NCS reaction with benzaldehydes as substrates^40^. The analogous iminium ion to **6** would be in a *trans*-conformation, oriented for *Re*-face attack by the pi-system, thus resulting in the (*S*)-quinone, followed by deprotonation to give a (*S*)-THIQ. This structure is also unique as it is the first crystallographic study of a *Tf*NCS variant and shows that it is highly conserved, compared to the WT structure. Residue V97 sits close to the alkyl region of the dopamine end of the putative iminium carbon of the mimic. The mutation from methionine to valine decreases both flexibility, and steric bulk at this position, so the reasons for increased conversions may be due to faster substrate binding. It is however unknown how conversions are improved with the M97F variant compared with the WT enzyme, and possible that there may be pi-stacking interactions with the benzaldehyde phenyl ring at some point in the mechanistic process, thus aiding substrate turnover.

### Increasing molecular complexity using *O*-methyltransferases

**Fig. 6:**
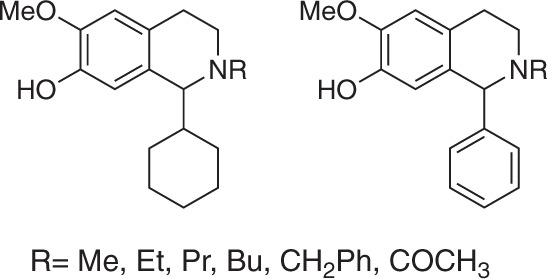
Examples of some patented *meta*-methoxylated, *N*-acylated 1,2,3,4-THIQs. The compounds shown have been patented for the treatment of a range of neurodegenerative diseases.

A variety of *meta*-methoxylated, *N*-acylated 1,2,3,4-THIQs (Fig. 6) are under patent for preventing or treating numerous degenerative or inflammatory diseases, by inhibiting the production of inflammatory substances by activated microglial cells^41^. Performing the regioselective *meta*-methylation of a catechol is challenging chemically, as there is a lack of regioselective control and synthetic routes, therefore, require the use of a precursor with the correct methylation pattern and/or employ a multi-step, protecting group strategy.

There are several research efforts into the discovery and isolation of regioselective methyltransferase enzymes, to avoid the challenges of synthetic, regioselective methylations^42^. Regioselective methylation reactions are also particularly useful for drug discovery purposes due to ‘the magic methyl effect’, whereby the substitution of H to CH_3_ can increase the potency of a drug molecule by up to 100-fold. In 2011, it was noted that methyl groups are found in >67% of the year’s top selling drugs^43^. Beyond the advantage of controlled regioselectivity, biocatalytic reactions involving methyltransferases are more environmentally benign than traditional, chemical alternatives as they avoid the use of carcinogenic alkylating agents (for example, methyl iodide) as the methyl donor. However, the major barrier to the widespread applicability of these enzymes in preparative-scale syntheses is the narrow substrate scope of many of the enzymes discovered and the requirement for stoichiometric quantities of the methyl donor, (*S*)-adenosyl-L-methionine (SAM). SAM is expensive and unstable, so reactions are not economically viable on a preparative scale. Recent efforts to overcome this have involved the use of an enzymatic co-factor generation system. Instead of using SAM directly, adenosine triphosphate (ATP) and L-methionine are added to the reaction with two enzymes so that SAM is formed in situ using a methionine adenosyl transferase (MAT, E.C.2.5.1.6), and the product (*S*)-adenosyl-L-homocysteine (SAH), is broken down by methylthioadenosine/SAH nucleosidase (MTAN, E.C.3.2.2.9) to prevent inhibition of the methyltransferase reaction^44^.

A range of regioselective catechol *O*-methyltransferases have been reported in the literature to accept various catecholamines as substrates, but THIQs have received little attention to date. Two catechol *O*-methyltransferases were selected; *Rn*COMT (isolated from *Rattus norvegicus*) and *Mx*SafC (isolated from *Myxococcus xanthus*)^45^. For reactions involving *Rn*COMT, methylation is most commonly observed on the *meta*-hydroxyl. The known substrate scope includes a variety of catecholamines (dopamine, dihydrocaffeic acid and 3,4-dihydroxybenzoic acid)^45,46^. The enzyme is often not completely regioselective, and some *para*-methylation can be observed^45^. On the other side, *Mx*SafC is reported to have complementary regioselectivity, with methylation favoured at the *para*-position with L-dopa, dopamine and caffeic acid, although a preference for the *meta*-position is observed with 3,4-dihydroxybenzoic acid^47^.

**Fig. 7:**
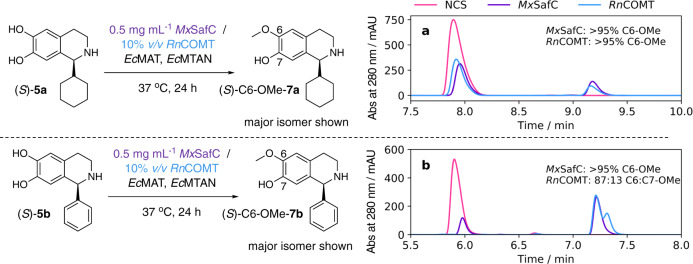
Achiral HPLC traces of the regioselective *meta*-*O*-methylation of two THIQ products (*S*)-5a and (*S*)-5b. **a** Methylation of **5a** to give **7a**; **b** methylation of **5b** to give **7b**. In all cases, the methylation system generating SAM is used to avoid the addition of stoichiometric quantities of SAM and is used with the *O*-methyltransferases *Rn*COMT and *Mx*SafC. The methyl donor, SAM is generated in situ by the enzyme *Ec*MAT and the reaction byproduct, SAH is broken down by another enzyme, *Ec*MTAN.

Methylation reactions were performed using analogous conditions to those detailed by Siegrist and co-workers^44^, except that unpurified preparation of the substrate and purified MAT enzyme from *Escherichia coli* (Supplementary Figs. 5 and 6, and Supplementary Methods) was used due to the increased activities with L-methionine, rather than *Tk*MAT (from *Thermococcus kodakarensis*), as previously reported^44^. The two *O*-methyltransferases (*Rn*COMT and *Mx*SafC; Supplementary Fig. 4 and Supplementary Methods) were tested with two (1 *S*)-1-substituted THIQ products (**5a** and **5b**) generated from the NCS reaction. Achiral HPLC traces of the reactions performed are shown in Fig. 7. The analytical HPLC trace of the product of the NCS reaction is also shown for comparison.

The regioselectivities of the resultant products were determined based on 2D-NMR spectroscopic data of the purified products and compared to the observed RP-HPLC traces. For methylations involving *Rn*COMT, a ratio of 87:13 of C6:C7-OMe-**7b** was observed by ^1^H-NMR when **5b** was used as the substrate (Fig. 7b) along with complete consumption of the starting material. Interestingly, altering the phenyl ring to a cyclohexyl ring, using **5a** as the substrate, gave remarkable regioselectivity, with only C6-OMe-**7b** observed (Fig. 7a). Perhaps the presence of a bulkier, more flexible ring at the C-1 position means that the catechol is only capable of binding to the magnesium-bound ion in the methyltransferase in a single orientation. Exceptional regioselectivities were also observed for reactions involving *Mx*SafC, with only *meta-*methylation observed in products **7a** and **7b** (Fig. 7), again giving C6-OMe-**7a and** C6-OMe-**7b**. This is interesting as in previous reports, the enzyme is predominantly shown to be regioselective towards the *para*-position, when dopamine (**1**) and dihydrocaffeic acid are used as substrates. However, when 3,4-dihydrobenzoic acid was used as a substrate, the enzyme was observed to methylate predominantly at the *meta*-position^45^. The regioselectivity is clearly highly dependent on the catechol side chain. The enantioselectivities towards **5a** and **5b** with both methyltransferases were determined based upon RP-HPLC and chiral HPLC analyses of reaction mixtures, compared to starting materials and product standards (Supplementary Methods, and Supplementary Figs. 27 and 28). *Rn*COMT was found to not be enantioselective, with complete consumption of *rac*-**5a** and *rac*-**5b** observed in methylation reactions. However *Mx*SafC was found to be selective towards both (*S*)-**5a** and (*S*)-**5b**. This provides valuable insights into future applications of such methyltransferases.

Preparative-scale reactions were performed using the most promising methyltransferase, *Mx*SafC. Conversions, when using 5 mM substrate in 20 mL reaction volume, were found to be 33% (**7a**) and 46% (**7b**), respectively, as determined by RP-HPLC analysis of the two products compared with calibration curves of purified product standards (achiral analytical HPLC method 2, Supplementary Fig. 26, and Supplementary Methods).

## Conclusions

In this work, a variety of benzaldehydes were shown to be readily accepted in *Tf*NCS-mediated reactions with both WT and single-point variant enzymes. Previous reactions were performed with lower enzyme and substrate concentrations: here, as well as increased enzyme concentrations, higher substrate loadings were successfully used (10–20 mM) due to the low *K*_d_ of dopamine binding with *Tf*NCS. The active site mutant, M97V proved particularly promising and was capable of generating a variety of (1 *S*)-aryl THIQ derivatives in high yields and high e.e.s. This route is advantageous as the chiral THIQ scaffold can be generated in a single step under benign conditions, avoiding the use of high temperatures, high pressures and toxic reagents. Oxidatively sensitive hydroxyl groups were also tolerated on the THIQ moiety, which is valuable as analogous compounds are biologically active. In addition, although high background reactions were observed with a variety of halogenated (fluorinated and chlorinated) benzaldehyde derivatives as substrates, when using high enzyme loading and lowering the reaction pH to 6, the contribution of the spontaneous background reaction decreased, enzyme activity improved and this enhanced the enantiopurity of the resultant THIQ products. The mutant M97V proved to be superior in generating a range of (1 *S*)-aryl-THIQs in high yields and e.e.s. A co-crystallised structure of this productive mutant (M97V) was gained with a non-productive reaction intermediate analogue (of the reaction between dopamine and benzaldehyde) bound in the active site. The ligand was found to be in a pre-cyclisation conformation, consistent with the generation of products with (1 *S*)-stereochemistry. In future studies, further mutagenesis at this position could be explored, possibly to even smaller sidechains on the amino acid.

The products of NCS reactions between dopamine and benzaldehyde or cyclohexanecarboxaldehyde were also accepted, as substrates by *O*-methyltransferases. Regioselective methylation of the *meta*-hydroxyl was achieved using SafC from *M. xanthus*, which is unusual as with other catecholamines, the methylation is predominantly selective towards the *para*-hydroxyl. Performing regioselective methylation reactions is challenging chemically and the products generated here are pharmaceutically relevant. The expansion of the portfolio of regioselective methyltransferases available will likely lead to increased conversions to give both *meta*- and *para*-methoxy products, thus generating useful analogues for drug discovery purposes.

## Methods

### Cloning and enzyme sequences

Plasmid details for M97V, L76V, M97F and WT Δ29*Tf*NCS have been previously reported by Lichman et al.^24^. Plasmid details for the methyltransferase enzymes, *Rn*COMT, *Mx*SafC, *Ec*MAT and *Ec*MTAN (Supplementary Methods) have been previously reported by Siegrist et al.^45^. For Δ33*Tf*NCS, a truncated (residues 33–196 only), codon-optimised construct of the WT-*Tf*NCS gene with a single-point mutation, M97V (M97V-∆33*Tf*NCS) with an N-terminal hexahistidine tag and Tobacco Etch Virus (TEV) protease cleavage site (N-6His-TEV-M97V-∆33*Tf*NCS) was synthesised and cloned into BL21(DE3). UniProt codes are: *Tf*NCS (Q67A25), *Rn*COMT (P22734), *Mx*SafC (Q50859), *Ec*MAT (D1LDF1) and *Ec*MTAN (A7ZHQ1). Protein sequences and full plasmid details are given in the Supplementary Methods.

### Protein expression

All plasmids were transformed into BL21(DE3) *E. coli*. A total of 100 mL LB medium was inoculated with a single colony and grown at 37 °C for 18 h. A total of 500 mL LB media was inoculated with 1% v/v of overnight culture and grown at 37 °C for 2 h shaking in flasks. Isopropylthiogalactoside (for all NCSs, 0.5 mM final concentration was used and for *Rn*COMT, *Mx*SafC, *Ec*MAT and *Ec*MTAN 0.2 mM final concentration was used) was added and the culture was incubated for 18 h at 25 °C (for all gene expressions except *Mx*SafC, where incubation was performed at 16 °C for 36 h). Cell pellets were isolated by centrifugation and stored at −20 °C until further purification or lysis for enzymatic reactions.

### Lysate preparation

To prepare *Rn*COMT lysate for enzymatic reactions, the pellet was resuspended in buffer (4% v/v of final culture volume, 50 mM HEPES, pH 7.5) and cells were lysed by sonication (10 s ON, 10 s OFF, 10×). The resulting suspension was centrifuged (10 min, 6000 × *g*, 4 °C) and the supernatant used directly for enzymatic reactions or stored at −20 °C.

### Protein purification

For purification of 6His-TEV-M97V-∆33*Tf*NCS and WT, M97F-V-∆29*Tf*NCS, L76V-∆29*Tf*NCS, *Mx*SafC and *Ec*MAT, the cell pellet was resuspended in lysis buffer (100 mM HEPES, 20 mM imidazole, 100 mM NaCl, pH 7.5, and 15% v/v culture volume) with a small amount of DNAse1. The cells were lysed by sonication (3 × (3 min ON, 3 min OFF)) and centrifuged (15,000 × *g*, 45 min, 4 °C). The resulting supernatant was removed and filtered (0.45 µm). A 5 mL His-trap HP column was equilibrated with lysis buffer and the lysate loaded onto the column at 1 mL min^−1^. The column was washed with lysis buffer to remove any unbound protein (six column volumes), followed by washings with a stepwise gradient of increasing imidazole concentrations by combining lysis buffer and elution buffer (100 mM HEPES, 500 mM imidazole, 100 mM NaCl, pH 7.5). The column was washed with 8% elution buffer for six column volumes, 16% elution buffer for six column volumes followed by 100% elution buffer. All washings were performed at a flow rate of 1 mL min^−1^. Fractions were analysed by SDS–PAGE (Supplementary Figs. 1, 4–6) and fractions containing pure protein were combined and dialysed into 20 mM Tris, 50 mM NaCl, pH 7.5 buffer. The protein sample was centrifuged and concentrated to ~10 mg mL^−1^. Aliquots of protein were stored at −80 °C until use for enzymatic reactions or in the case of 6His-TEV-M97V-∆33*Tf*NCS, taken through to the next step of purification for removal of the hexahistidine tag.

For removal of the hexahistidine tag of 6His-TEV-M97V-∆33*Tf*NCS, TEV protease was added to the pooled fractions and the sample dialysed overnight (20 mM Tris, 50 mM NaCl, pH 7.5). The protein sample was then centrifuged (15,000 × *g*, 20 min, 4 °C) and loaded onto a 5 mL His-trap HP column. The supernatant was passed through the column. The column was then washed with varying amounts of imidazole-containing elution buffer (20 mM Tris, 50 mM NaCl, 500 mM imidazole, pH 7.5) mixed with the dialysis buffer. Fractions containing M97V-∆33*Tf*NCS were pooled (Supplementary Fig. 2) and further purified by gel filtration on a Superdex S75 column. Fractions within the major UV absorbance peak were pooled (Supplementary Fig. 3), concentrated (to ~10 mg mL^−1^) and stored at −80 °C.

### Protein crystallisation

NCS protein crystals were prepared by the sitting-drop method in 25% w/v PEG 1500, 20% glycerol. M97V-Δ33*Tf*NCS (12.6 mg mL^−1^ in 20 mM Tris, 50 mM NaCl) was prepared in a 95:5 ratio with the mimic **6** (at 200 mM in DMSO), resulting in final mimic concentration of 10 mM. Crystals were grown for 8 weeks at 20 °C then cryo-protected in the crystallisation condition containing 10 mM mimic. Diffraction data were collected at Diamond beamline i04. The images for both datasets were integrated using the XDS Dials software programme. Refinement of both datasets was performed using an apo dataset of Δ33*Tf*NCS (WT) in the same space group (*P*3_2_21) as the search model in Phaser^48^. Model building of both the protein and the ligand was performed using COOT^49^ with refinement performed, using REFMAC5 using TLS refinement^50^. Figures were prepared using USCF Chimera^51^. Data collection and refinement statistics are given in Supplementary Table 1.

### Analytical RP-HPLC analysis

Analysis of samples was performed using an Agilent 1260 Infinity liquid chromatography system comprising of a G1329B autosampler, G1311C quaternary pump, 1260 G1316A column oven and 1260 G1314F variable wavelength detector. The system was equipped with a HiChrom ACE C18 column (250 mm × 4.6 mm). Achiral HPLC method 1: acetonitrile (MeCN) in water (0.1% v/v TFA) was used as the mobile phase. The gradient used was: 10% MeCN for 1 min, a linear gradient to 70% MeCN over 5 min, 100% MeCN for 30 s, followed by 10% MeCN for 3.5 min. A flow rate of 1 mL min^−1^ was used. The initial reaction volume was diluted to 25% reaction concentration and 10 μL of sample was injected. UV absorbance was measured at 280 nm. Achiral HPLC method 2: MeCN in water (0.1% v/v TFA) was used as the mobile phase. The gradient used was: 10% MeCN for 1 min, a linear gradient to 70% MeCN over 15 min, 100% MeCN for 30 s, followed by 10% MeCN for 3.5 min. A flow rate of 1 mL min^−1^ was used. The initial reaction volume was diluted to 25% reaction concentration and 10 μL of sample was injected. UV absorbance was measured at 280 nm.

### Chiral HPLC analysis

Samples were analysed using a Hewlett Packard Series 1100 liquid chromatography system consisting of a G1311A Quaternary Pump, G1313A autosampler and G1314A variable wavelength detector (methods 2 and 3). Samples analysed by method 1 used the same system as all achiral analytical HPLC samples. Chiral HPLC method 1: an Agilent InfinityLab Poroshell 120 Chiral-V column (150 mm × 4.6 mm, 2.7 mM) was used with an isocratic MeOH (0.2 wt% ammonium formate) mobile phase at 1 mL min^−1^. UV absorbance at 280 nm was measured. The column temperature used was 30 °C. Chiral HPLC method 2: a Diacel Chiralpak® AD-H column (250 mm × 4.6 mm, 5 mM) was used with an isocratic *n*-hexane:ethanol:diethylamine (80:20:0.01) mobile phase at 1 mL min^−1^. UV absorbance at 280 nm was measured. The column temperature used was 30 °C. Chiral HPLC method 3: a Diacel Chiralpak® OD column (250 mm × 4.6 mm, 5 mM) was used with an isocratic *n*-hexane:isopropanol:diethylamine (90:10:0.1) mobile phase at 1 mL min^−1^. UV absorbance was measured at 280 nm. The column temperature used was 20 °C (ref. ^37^). Chiral HPLC method 4: a Diacel Chiralpak® OD column (250 mm × 4.6 mm, 5 mM) was used with an isocratic *n*-hexane:isopropanol:diethylamine (95:5:0.1) mobile phase at 1 mL min^−1^. UV absorbance was measured at 280 nm. The column temperature used was 20 °C (ref. ^37^).

Chiral HPLC traces are given in the Supplementary Information (Supplementary Figs. 10–22).

### Semi-preparative HPLC

Reverse-phase, semi-preparative HPLC was performed on a Dionex 580 HPLC machine with a PDA-100 photodiode array detector, P580 pump and a model ASI-100 automated sample injector. The column used was a Phenomenex Onyx C18 (100 × 10 mm). Water (0.1% v/v TFA) and MeCN (0.1% v/v TFA) were used as the mobile phase, using a linear gradient of 5–95% MeCN over 36 min. A flow rate of 2 mL min^−1^ was used with detection at 254 nm.

### Preparative HPLC

Purification was performed using Agilent 1200 Infinity series liquid chromatography system equipped with G1361A prep pump, G2260A autosampler, G1364A fraction collector and G7165 multiple wavelength detector. A Supelco Discovery®BIO Wide Pore C18-10 column (25 cm × 21.2 mm, 10 μM) using MeCN and water (both solvents contained 0.1% *v/v* TFA) as the mobile phase. UV absorbance was measured at 280 nm. Preparative HPLC method 1: 20-min gradient 25–95% MeCN (0.1% v/v TFA) in water (0.1% v/v TFA). Preparative HPLC method 2: 20-min gradient 35–80% MeCN (0.1% v/v TFA) in water (0.1% v/v TFA). Preparative HPLC method 3: 30-min gradient 5–95% MeCN (0.1% v/v TFA) in water (0.1% v/v TFA).

### Chemical reagents

All reagents were obtained from commercial sources (Sigma Aldrich, Fisher, Alfa Aesar) and used as received. Silica column chromatography was performed using Geduran® Si 60 Silica (43–60 μM). Thin layer chromatography was performed using plates with a silica gel matrix on an aluminium support. Ultraviolet light (254 nm) and ninhydrin stain was used to visualize the plates.

### Chemical analytics

^1^H and ^13^C NMR spectra were obtained using a Bruker Advance III 700 MHz spectrometer. Chemical shifts specified are relative to trimethylsilane (set at 0 p.p.m.) and referenced to the residual, protonated NMR solvent. Coupling constants in ^1^H-NMR spectra (*J*) are given in Hertz (Hz), and described as singlet (s), doublet (d), doublet of doublets (dd), triplet (t), quartet (q) and multiplet (m). NMR spectra for products are shown in Supplementary Figs. 32–72. Mass spectrometry data was obtained using a Waters Aquity UPLC-MS system (LRMS [ES+]). Infrared data were obtained using a Bruker Alpha Platinum-ATR machine.

### NCS-catalysed reactions

Enzymatic assays were performed on a 100 μL scale and larger-scale biotransformations were performed on a 10 mL scale. A solution of amine (final reaction concentration of 10 mM) and sodium ascorbate (final reaction concentration of 10 mM) was prepared in HEPES buffer (50 mM, pH 7.5 (except Fig. 1c where pH 6 was used)). A solution of aldehyde (200 mM in MeCN) was prepared and the two solutions mixed in a 9:1 ratio. *Tf*NCS (at ~10 mg mL^−1^ in 20 mM Tris, 50 mM NaCl, pH 7.5) was added and the reactions stirred at 37 °C. Control reactions were performed using the same conditions but the *Tf*NCS sample was substituted for enzyme buffer (20 mM Tris, 50 mM NaCl, pH 7.5). To quench the reactions, different conditions were used depending on the use of the sample. Workup method 1 (for RP-HPLC analysis): reactions were quenched by the addition of an equal volume of MeCN. Samples were centrifuged (16,000 × *g*, 10 min, 4 °C), the supernatant treated with an equal volume of water and analysed immediately by analytical RP-HPLC or stored at −20 °C. Workup method 2 (for chiral HPLC analysis): the reaction was saturated by addition of NaHCO_3_ and an equal volume of EtOAc to the reaction volume was added. The samples were vortexed (30 s) and centrifuged (16,000 × *g*, 5 min, 4 °C). The organic layer was removed and left to evaporate until dryness (ca. 48 h). The sample was resuspended in 80:20:0.1 *n-*hexane:ethanol:diethylamine (1.5 × initial reaction volume) and injected for HPLC analysis. Workup method 3 (for methyltransferase reactions): reactions were quenched by the addition of an equal volume of MeCN. Samples were centrifuged (16,000 × *g*, 10 min, 4 °C) and the supernatant removed and lyophilised.

### Methyltransferase reactions^44^

The lyophilised NCS reaction mixture (generated using optimised reaction conditions, Fig. 4) prepared by workup method 3 was resuspended in buffer (250 mM HEPES, 200 mM MgCl_2_, 2 M KCl, pH 7.5) and water (1:4 ratio) in an equal volume to the NCS reaction sample. ATP (100 mM in H_2_O, final concentration 10 mM), L-methionine (100 mM in H_2_O, final concentration 10 mM), *Ec*MAT (9 mg mL^−1^ purified, 0.4 mg mL^−1^ final concentration), *Ec*MTAN (2.5 mg mL^−1^ purified, 0.025 mg mL^−1^ final concentration) and methyltransferase (*Rn*COMT: 10% v/v lysate or *Mx*SafC: 6 mg mL^−1^ purified, 0.6 mg mL^−1^ final concentration) were added. Reaction volumes were adjusted by addition of HEPES buffer (50 mM, pH 7.5). Reactions were performed at 37 °C, 500 r.p.m. (200 μL scale) or 150 r.p.m. (10–20 mL scale) for 24 h. Products were prepared by preparative HPLC method 2.

### Calibration curves

All calibration curves used for determination of conversions to give products are given the Supplementary Information (Supplementary Figs. 24–26). Preparation of the calibration curves is described in the Supplementary Methods.

### Complete chemical syntheses and analyses

Complete synthetic methods, characterisation of THIQ products and corresponding chiral HPLC data are given in the Supplementary Information.

## Supplementary information

Supplementary Information

## Data Availability

Data to support this work is available from the corresponding authors upon reasonable request. Structural data for the co-crystallised structure of M97V-Δ33*Tf*NCS can be found at PDB: 6Z82.
