# Supplementary material for: Single step syntheses of (1S)-aryl-tetrahydroisoquinolines by norcoclaurine synthases
Source: Commun Chem. 2020 Nov 13;3:170. doi: 10.1038/s42004-020-00416-8 (PMC9814250; doi:10.1038/s42004-020-00416-8)
Supplement: Supplementary file 1 — Supplementary Information [file 42004_2020_416_MOESM1_ESM.pdf]

Supplementary Figures

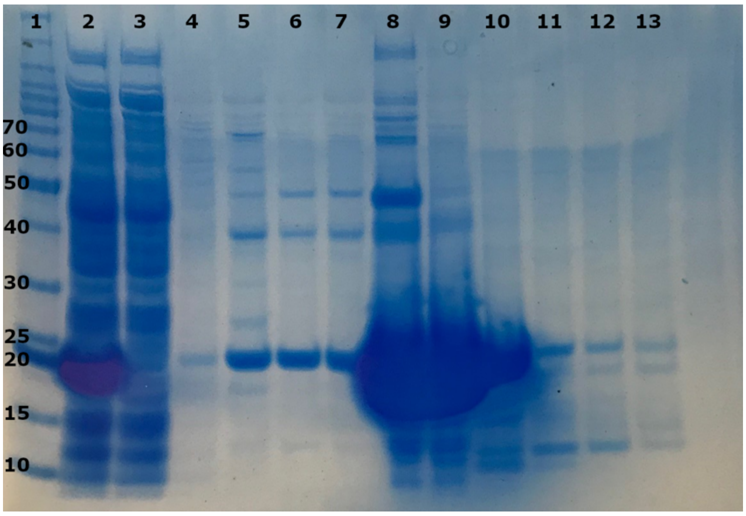

Supplementary Figure 1. SDS-PAGE analysis of His-trap purification of M97V-Δ33*Tf*NCS. CV = column volume. Lanes: 1, Benchmark™ Protein Ladder (masses given in kDa) 2, clarified cell lysate loaded onto column. 3, flow through with lysis buffer. 4, wash with 6 CV 20 mM imidazole buffer. 5, wash with 6 CV 40 mM imidazole buffer. 6-13, wash with 500 mM imidazole buffer and collected 3 mL fractions.

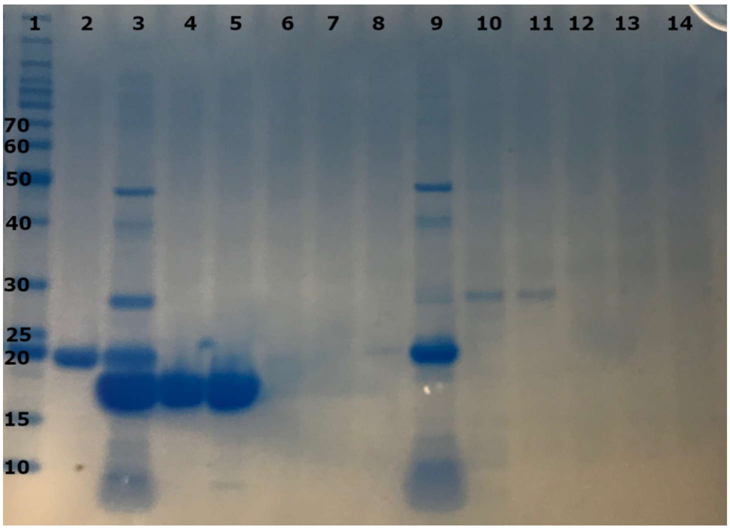

Supplementary Figure 2. SDS-PAGE analysis of His-trap purification of M97V-Δ33*Tf*NCS to remove TEV protease. CV = column volume. Lanes: 1, Benchmark™ Protein Ladder, masses given in kDa. 2, Pre-TEV cleavage sample (Lane 7 from gel 1) 3, sample loaded onto column. 4, flow through with lysis buffer. 5, 3 CV 20 mM imidazole. 6, 8 CV 20 mM imidazole. 7, 2 CV 40 mM imidazole. 8, 5 CV 40 mM imidazole. 9 and 10, 2 CV 500 mM imidazole. 11, 5 CV 500 mM imidazole.

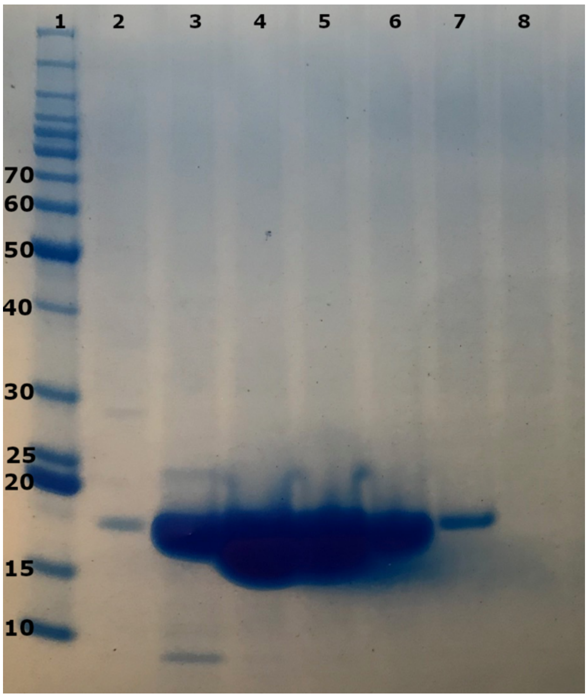

Supplementary Figure 3. SDS-PAGE analysis of gel filtration of M97V-Δ33*Tf*NCS: gel of peaks isolated from major peak from Superdex S75 gel filtration. Lane 1: Benchmark™ Protein Ladder, masses given in kDa.

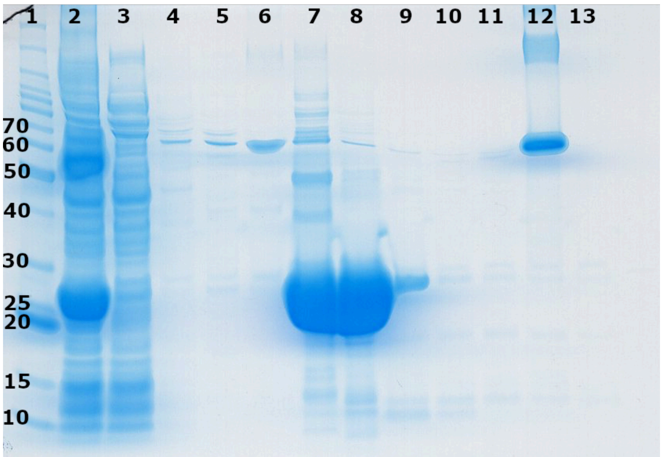

Supplementary Figure 4. SDS-PAGE analysis of His-trap purification of *Mx*SafC. CV = column volume. Lanes: 1, Benchmark™ Protein Ladder (masses given in kDa) 2, clarified cell lysate loaded onto column. 3, flow through with lysis buffer. 4, wash with 6 CV 20 mM imidazole buffer. 5, wash with 6 CV 40 mM imidazole buffer. 6-13, wash with 500 mM imidazole buffer and collected 3 mL fractions.

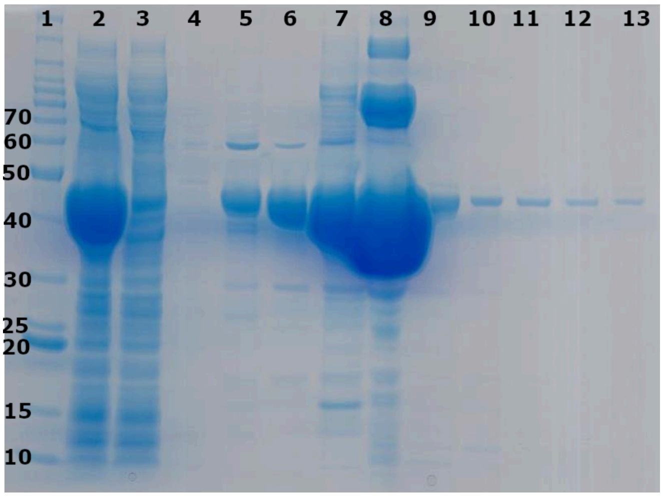

Supplementary Figure 5. SDS-PAGE analysis of His-trap purification of *Ec*MAT. CV = column volume. Lanes: 1, Benchmark™ Protein Ladder (masses given in kDa) 2, clarified cell lysate loaded onto column. 3, flow through with lysis buffer. 4, wash with 6 CV 20 mM imidazole buffer. 5, wash with 6 CV 40 mM imidazole buffer. 6-13, wash with 500 mM imidazole buffer and collected 3 mL fractions.

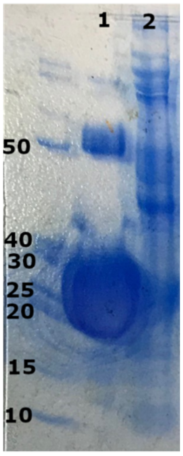

Supplementary Figure 6. SDS-PAGE analysis of Lane 1: *Ec*MTAN purification, Lane 2: *Rn*COMT expression.

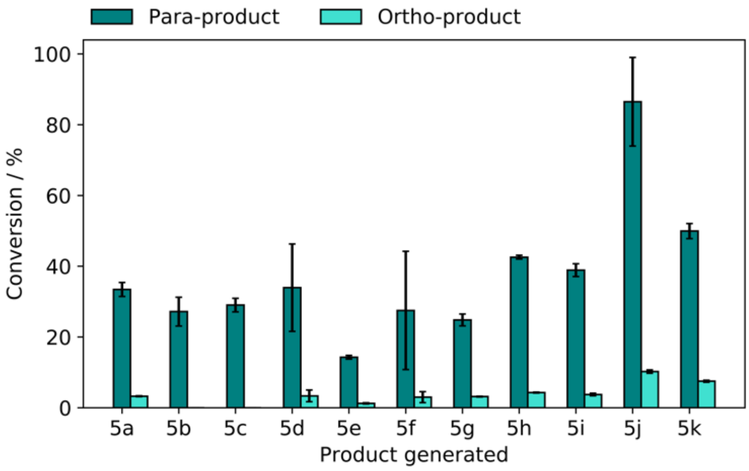

Supplementary Figure 7. Conversions of phosphate-mediated reactions between dopamine and a variety of benzaldehyde analogues. *Reaction conditions***:** 10 mM dopamine HCl, 10 mM sodium ascorbate, 20 mM benzaldehyde in KPi buffer (300 mM, pH 6) with 50% *v/v* MeCN. Reactions were performed on a 200 μL scale for 18 h at 60 °C. Samples were prepared by workup method 1. Yields were determined by monitoring product formation against standards by analytical RP-HPLC (method 1). Reactions were performed in triplicate and standard deviations reported.

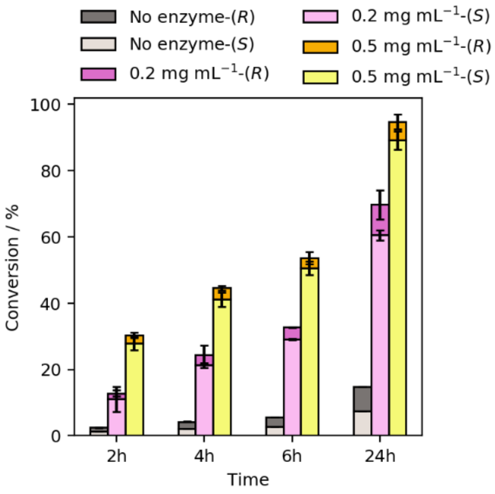

Supplementary Figure 8. Conversions and product stereochemistry for reactions between dopamine and 3-methylbenzaldehyde (**4d**) to give **5d**, catalysed by Δ29*Tf*NCS-M97V at different concentrations. *Reaction conditions***:** 10 mM dopamine HCl, 10 mM sodium ascorbate, 20 mM benzaldehyde in HEPES buffer (50 mM, pH 7.5) with 10% *v/v* MeCN and concentration of *Tf*NCS at 0.2 or 0.5 mg mL^{-1}. Control reactions were performed using the same conditions but the *Tf*NCS sample was substituted for enzyme buffer (20 mM Tris, 50 mM NaCl, pH 7.5). Reactions were performed at 37 °C for 3 h, 100 µL scale reactions; samples were prepared by workup method 1, yields were determined by monitoring product formation against standards by analytical achiral HPLC (method 1). Reactions were performed in triplicate and standard deviations reported.

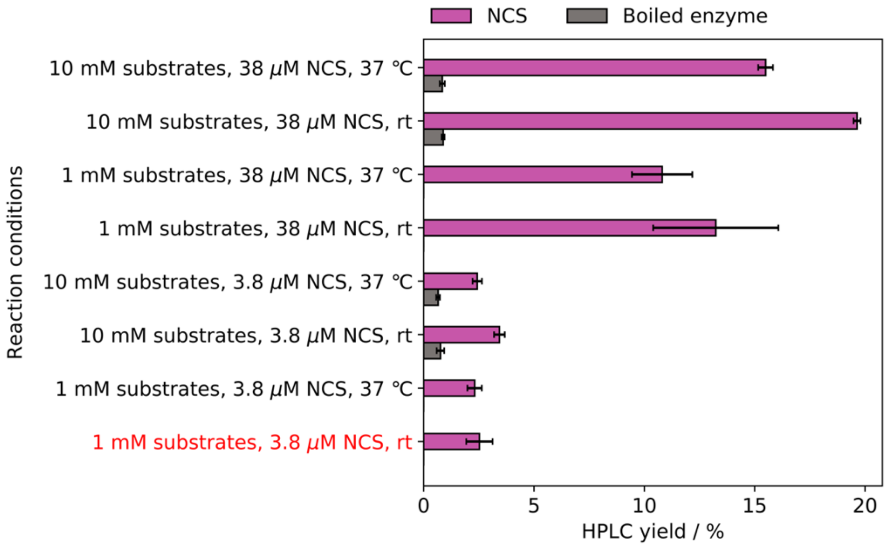

Supplementary Figure 9. Yields of product formation of **5b** following reaction conditions described in O'Connor *et al.*[1] (given in red). Reaction conditions were also altered to determine the cause of low conversions. *Reaction conditions*: Stock solutions of dopamine (100 mM in MeOH) and benzaldehyde (100 mM in MeOH) were prepared and added to TRIS buffer (100 mM, pH 7) and Δ29*Tf*NCS (stock at 480 μM in 20 mM TRIS, 50 mM NaCl, pH 7.5). Reactions were prepared to 100 μL and were performed for 3 h. Boiled enzyme was used as the negative control. Reactions were quenched by addition of 100 μL MeOH, centrifuged (13,000 rpm, 10 min, 4 °C) and the supernatant analysed by RP-HPLC (method 1). Conversions were determined based upon a calibration curve of **5b** (Supplementary Figure 24). Reactions were performed in triplicate and standard deviations reported.

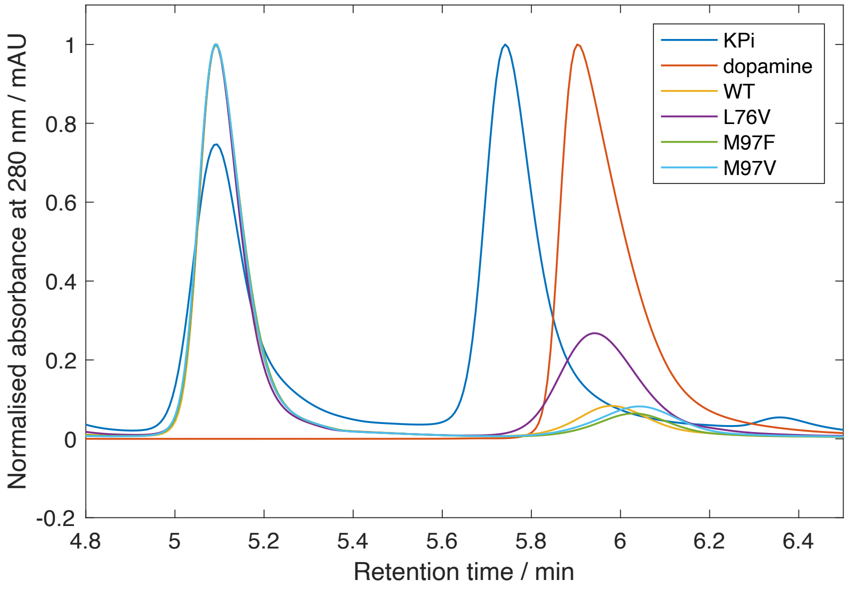

Supplementary Figure 10. Chiral HPLC analysis of **4a** by chiral HPLC method 1 using *Tf*NCS variants as reaction catalysts for the reaction between **1** and **4a**. *Preparation of dopamine* sample: A solution of dopamine HCl (5 mM) and sodium ascorbate (5 mM) was prepared. Retention time (t_R) (major, *S* enantiomer) = 5.1 min, t_R (minor, *R* enantiomer) = 5.7 min.

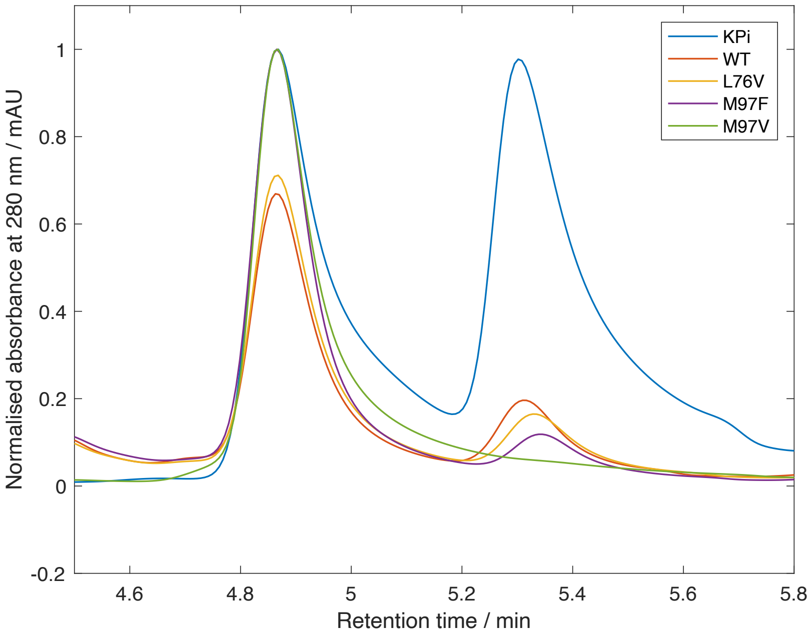

Supplementary Figure 11. Chiral HPLC analysis of **5b** by chiral HPLC method 1 using various different *Tf*NCS variants as reaction catalysts for the reaction between **1** and **5a**. t_R (major, *S* enantiomer) = 4.9 min, t_R (minor, *R* enantiomer) = 5.3 min.

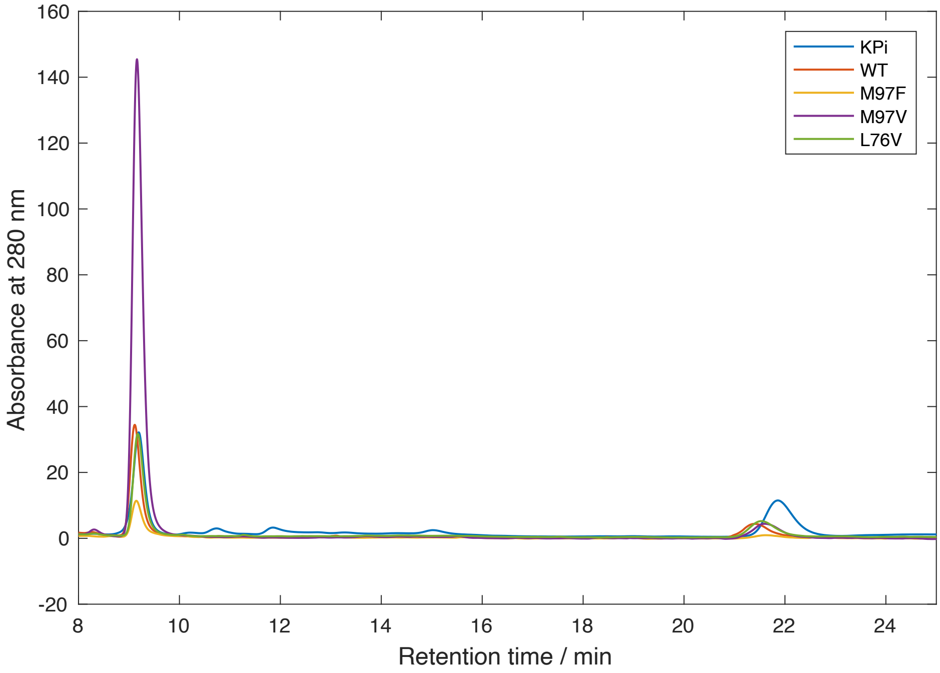

Supplementary Figure 12. Chiral HPLC analysis of **5c** by chiral HPLC method 2 using various different *Tf*NCS variants as reaction catalysts for the reaction between **1** and **5c**. t_R (major, *S* enantiomer) = 9 min, t_R (minor, *R* enantiomer) = 22 min.

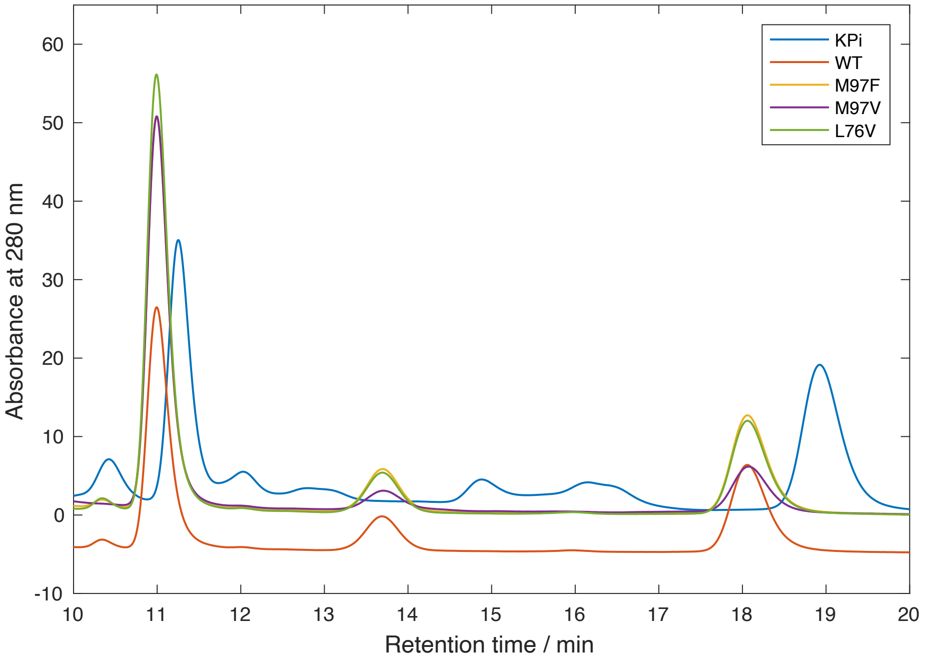

Supplementary Figure 13. Chiral HPLC analysis of **5d** by chiral HPLC method 2 using various different *Tf*NCS variants as reaction catalysts for the reaction between **1** and **4d**. t_R (major, *S* enantiomer) = 11 min, t_R (minor, *R* enantiomer) = 18-19 min.

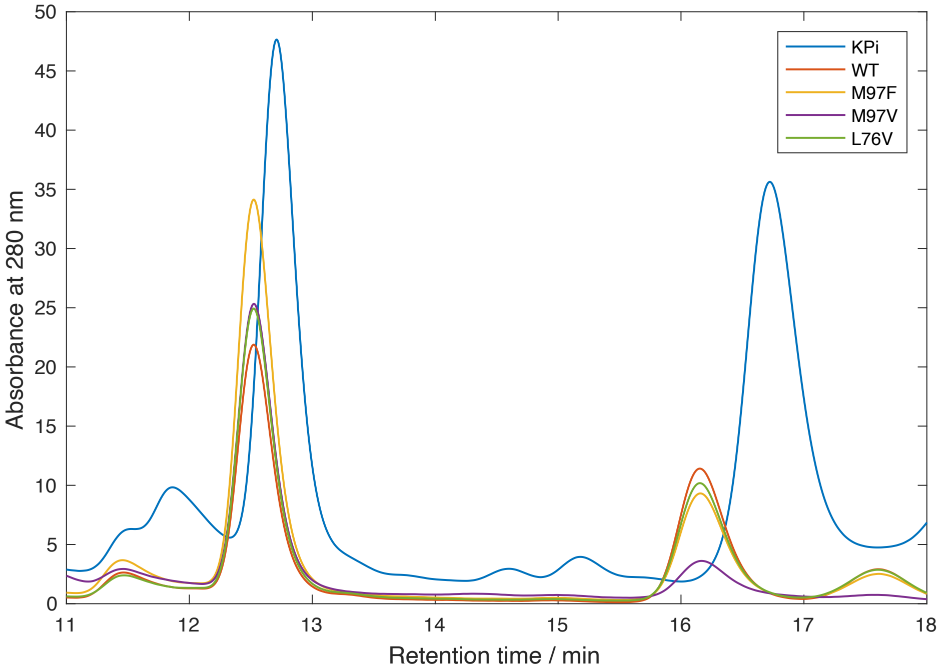

Supplementary Figure 14. Chiral HPLC analysis of **5e** by chiral HPLC method 2 using various different *Tf*NCS variants as reaction catalysts for the reaction between **1** and **4e**. t_R (major, *S* enantiomer) = 12-13 min, t_R (minor, *R* enantiomer) = 16 – 17 min.

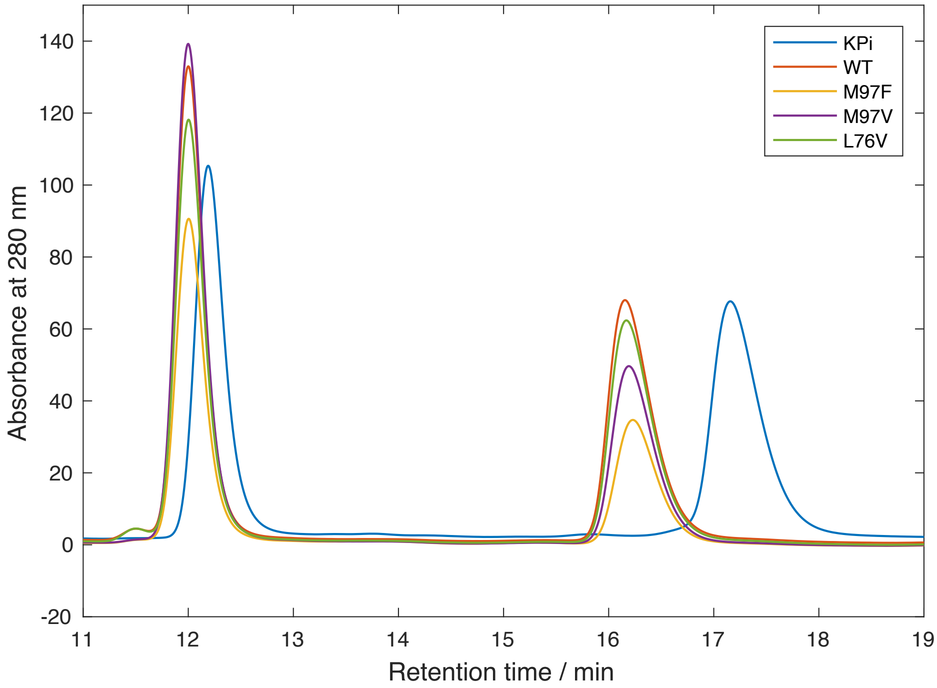

Supplementary Figure 15. Chiral HPLC analysis of **5f** by chiral HPLC method 2 using various different *Tf*NCS variants as reaction catalysts for the reaction between **1** and **4f**. t_R (major, *S* enantiomer) = 11 min, t_R (minor, *R* enantiomer) = 16-17 min.

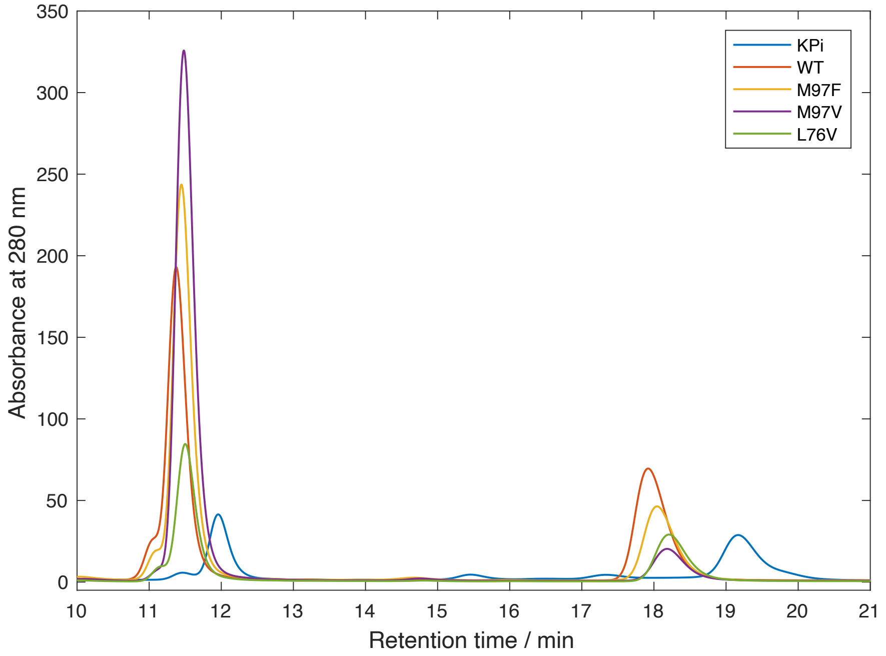

Supplementary Figure 16. Chiral HPLC analysis of **5g** by chiral HPLC method 2 using various different *Tf*NCS variants as reaction catalysts for the reaction between **1** and **4g**. t_R (major, *S* enantiomer) = 11 min, t_R (minor, *R* enantiomer) = 17-20 min.

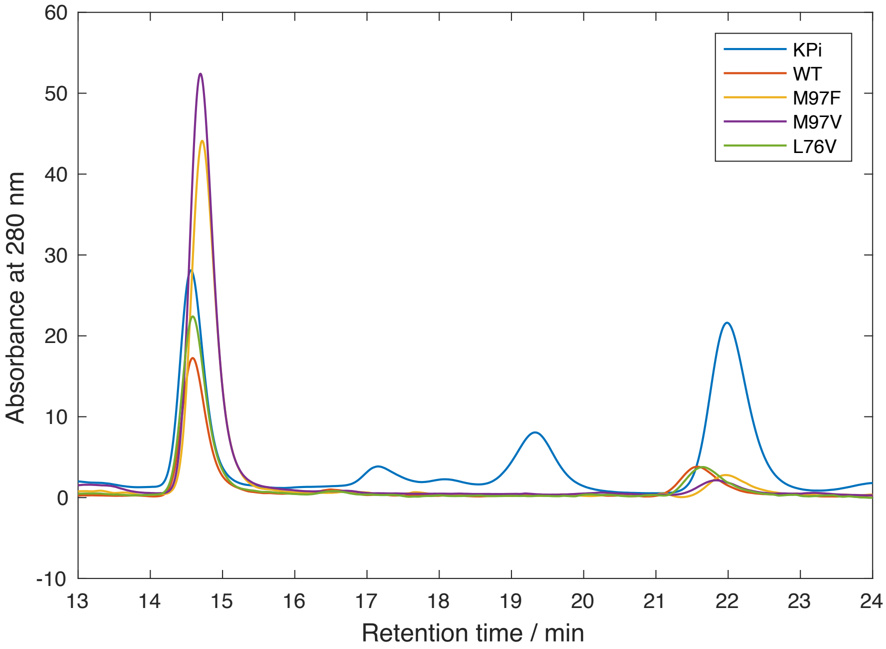

Supplementary Figure 17. Chiral HPLC analysis of **5h** by chiral HPLC method 2 using various different *Tf*NCS variants as reaction catalysts for the reaction between **1** and **4h**. t_R (major, *S* enantiomer) = 14-15 min, t_R (minor, *R* enantiomer) = 21-23 min.

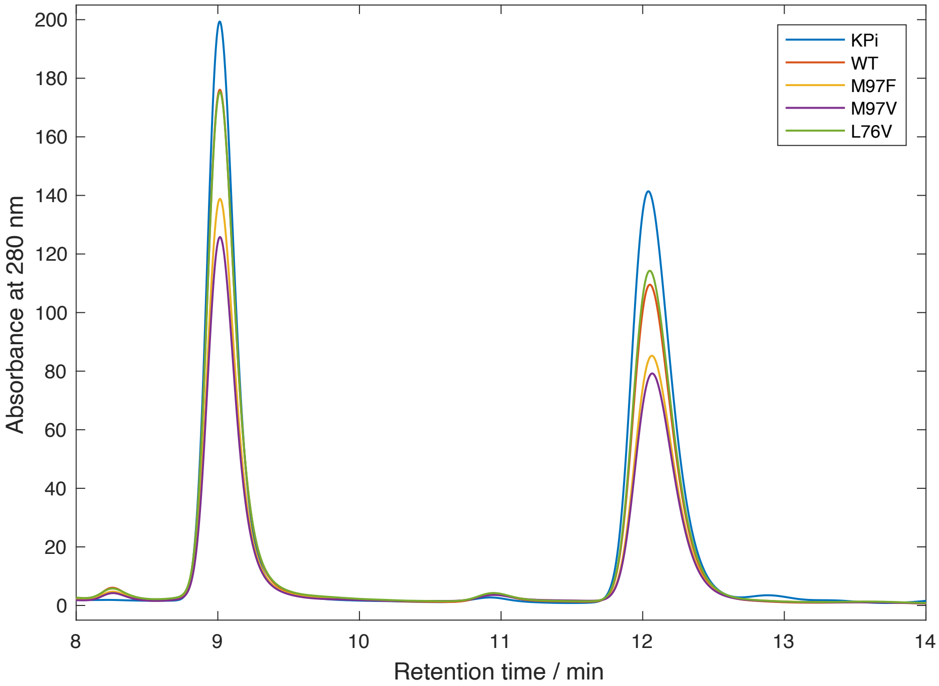

Supplementary Figure 18. Chiral HPLC analysis of **5i** by chiral HPLC method 2 using various different *Tf*NCS variants as reaction catalysts for the reaction between **1** and **4i**. t_R (major, *S* enantiomer) = 9 min, t_R (minor, *R* enantiomer) = 12 min.

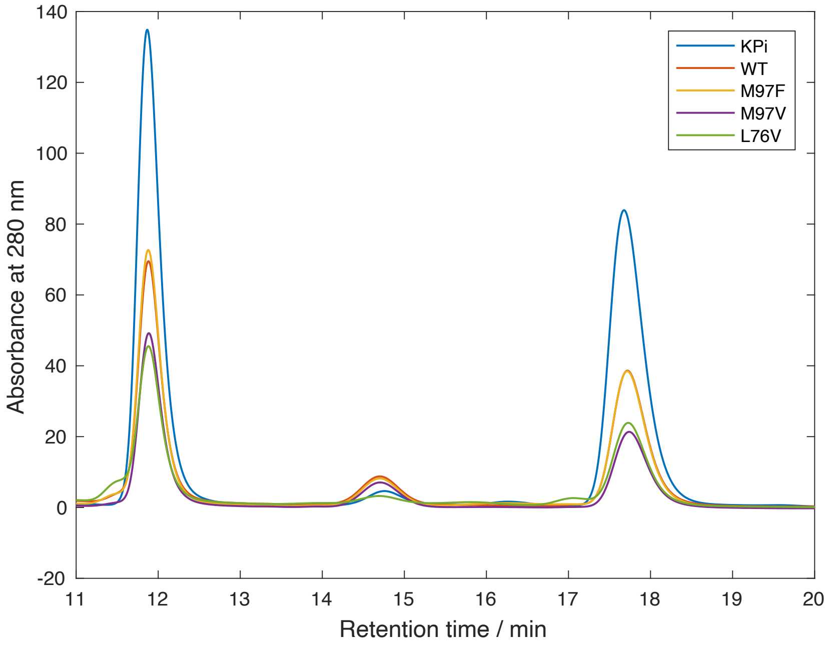

Supplementary Figure 19. Chiral HPLC analysis of **5j** by chiral HPLC method 2 using various different *Tf*NCS variants as reaction catalysts for the reaction between **1** and **4j**. t_R (major, *S* enantiomer) = 12 min, t_R (minor, *R* enantiomer) = 17-18 min.

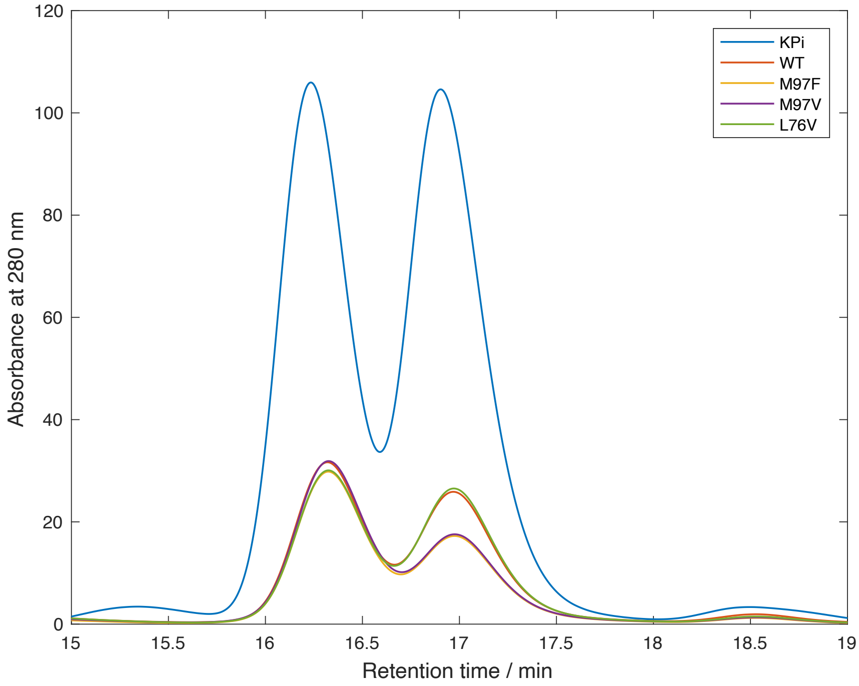

Supplementary Figure 20. Chiral HPLC analysis of **5k** by chiral HPLC method 2 using various different *Tf*NCS variants as reaction catalysts for the reaction between **1** and **4k**. t_R (major, *S* enantiomer) = 16 min, t_R (minor, *R* enantiomer) = 17 min.

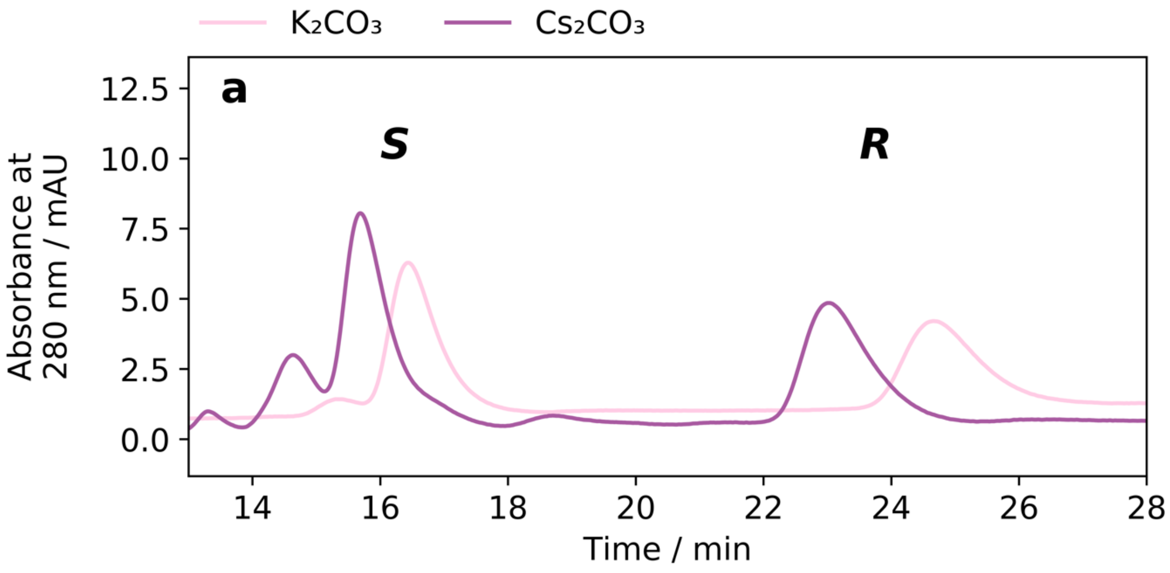

Supplementary Figure 21. Chiral HPLC analysis of (*S*)-6,7-dimethoxy-1-phenyl-1,2,3,4-tetrahydroisoquinoline by chiral HPLC method 3. t_R (*S* enantiomer) = 16.2 min, 564.2 mAU^2, t_R (*R* enantiomer) = 24.3 min, 499.6 mAU^2.

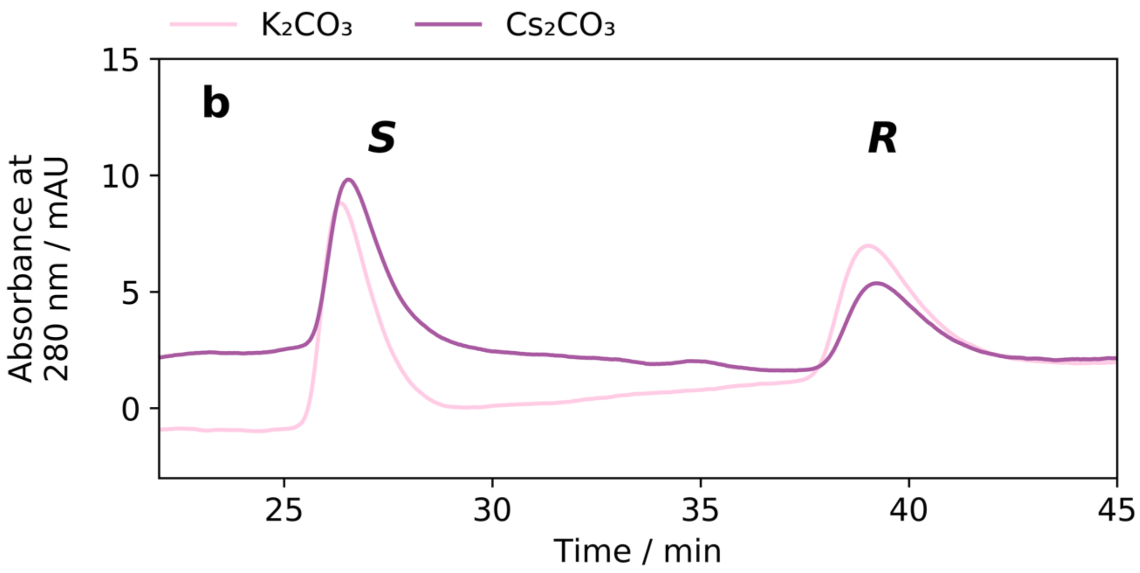

Supplementary Figure 22. Chiral HPLC analysis of (*S*)-6,7-dimethoxy-1-phenyl-1,2,3,4-tetrahydroisoquinoline by chiral HPLC method 4. t_R (*S* enantiomer) = 26.5 min, t_R (*R* enantiomer) = 39.2 min.

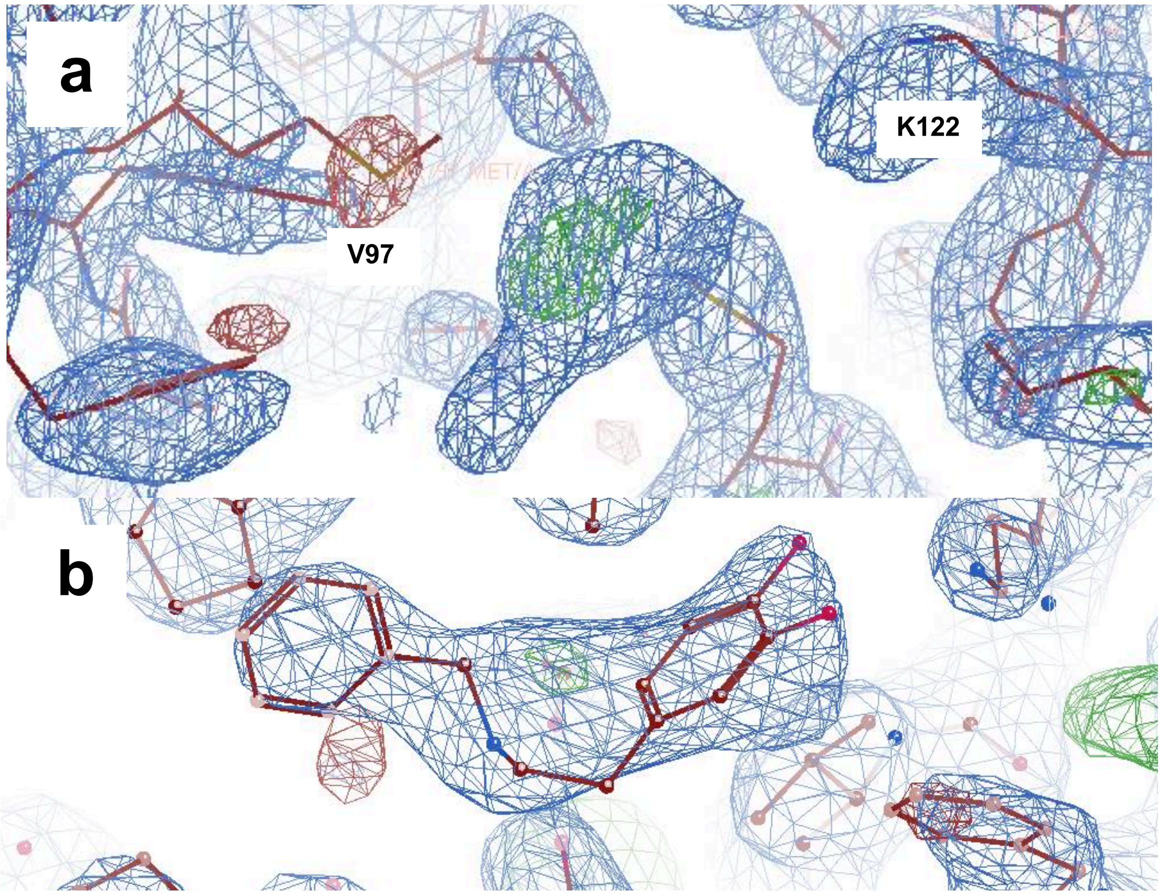

Supplementary Figure 23. a) Positive difference density in the enzyme active site (at 1.06 RMSD), close to K122, first observed in the active site directly after molecular replacement. Increased positive density was observed with further rounds of refinement. Negative difference density is observed at the side chain of residue 97, confirming the presence of the mutation M97V. b) Electron density of the bound mimic, **6** at 0.93 RMSD. Both images prepared in COOT.[2]

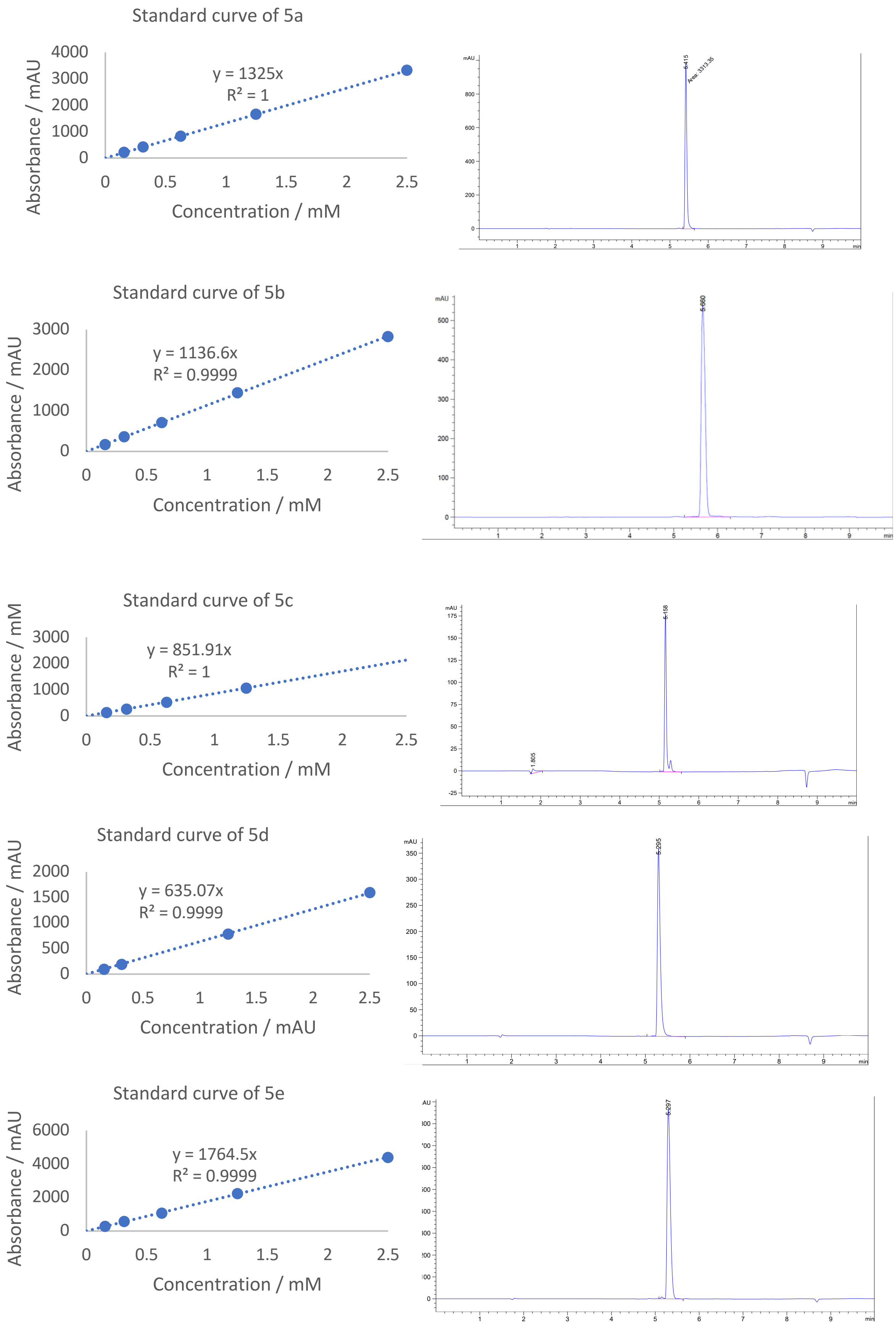

Supplementary Figure 24. Calibration curves and representative HPLC traces of compounds **5a-e**.

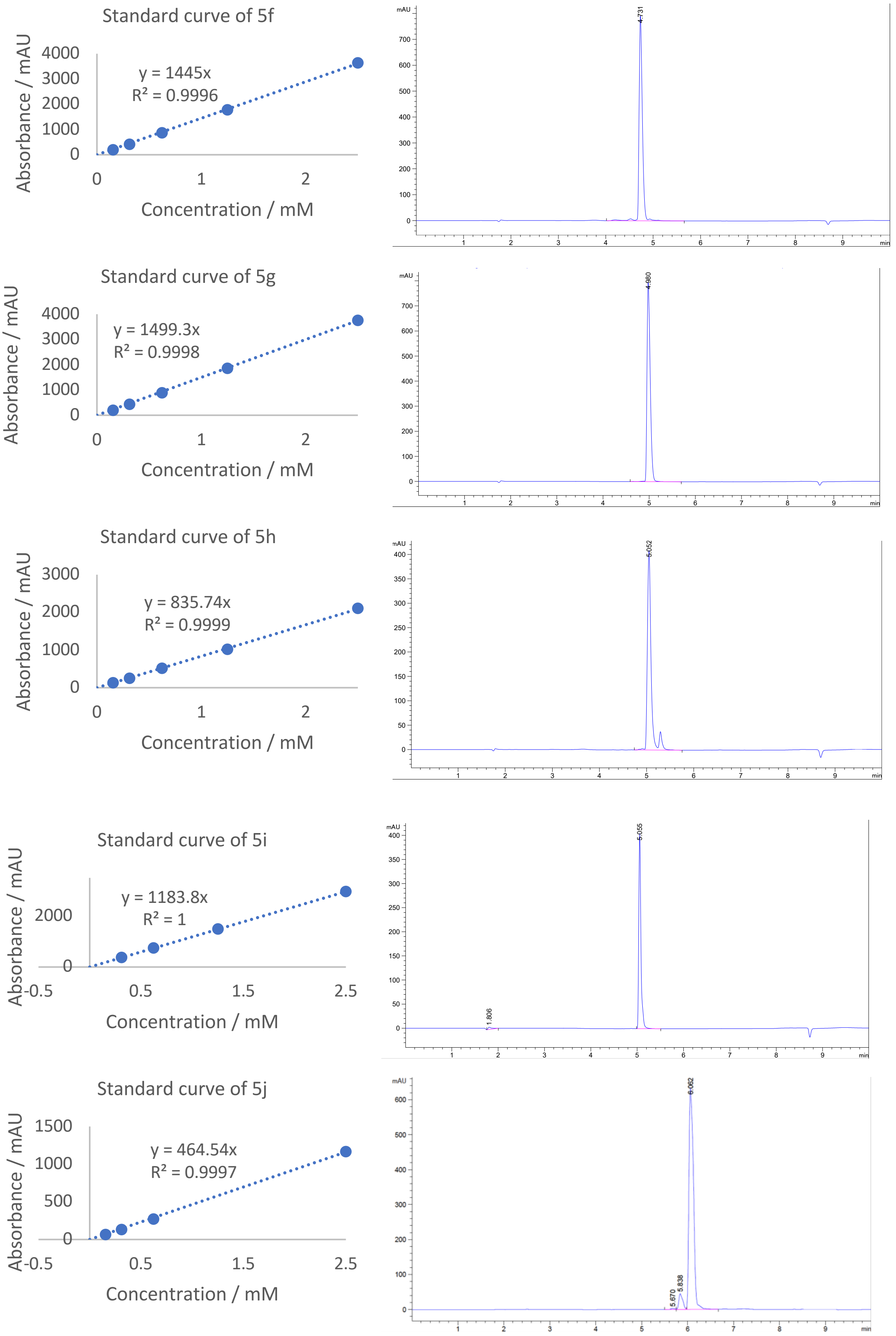

Supplementary Figure 25. Calibration curves and representative HPLC traces of compounds **5f-j**.

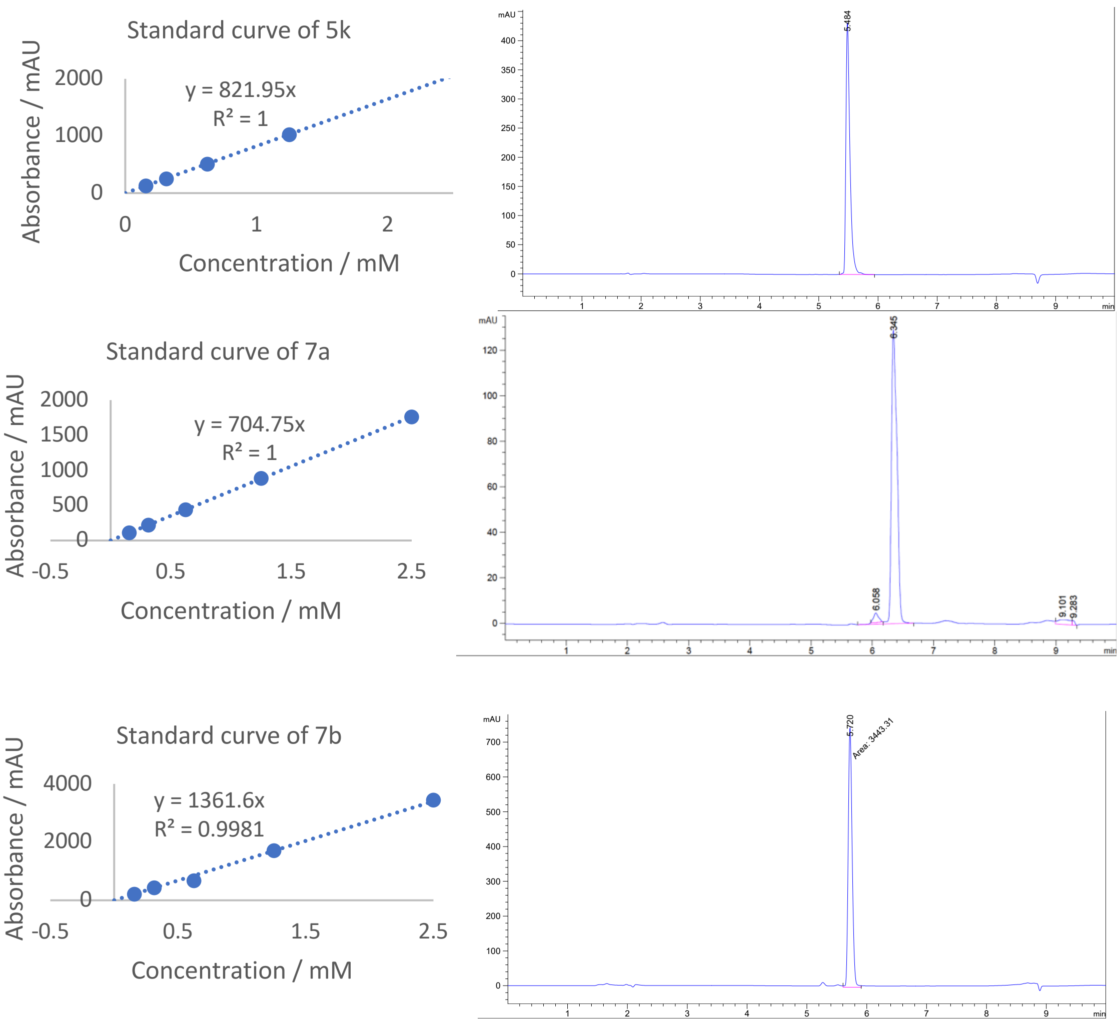

Supplementary Figure 26. Calibration curves and representative HPLC traces of compounds **5k, 7a** and **7b**.

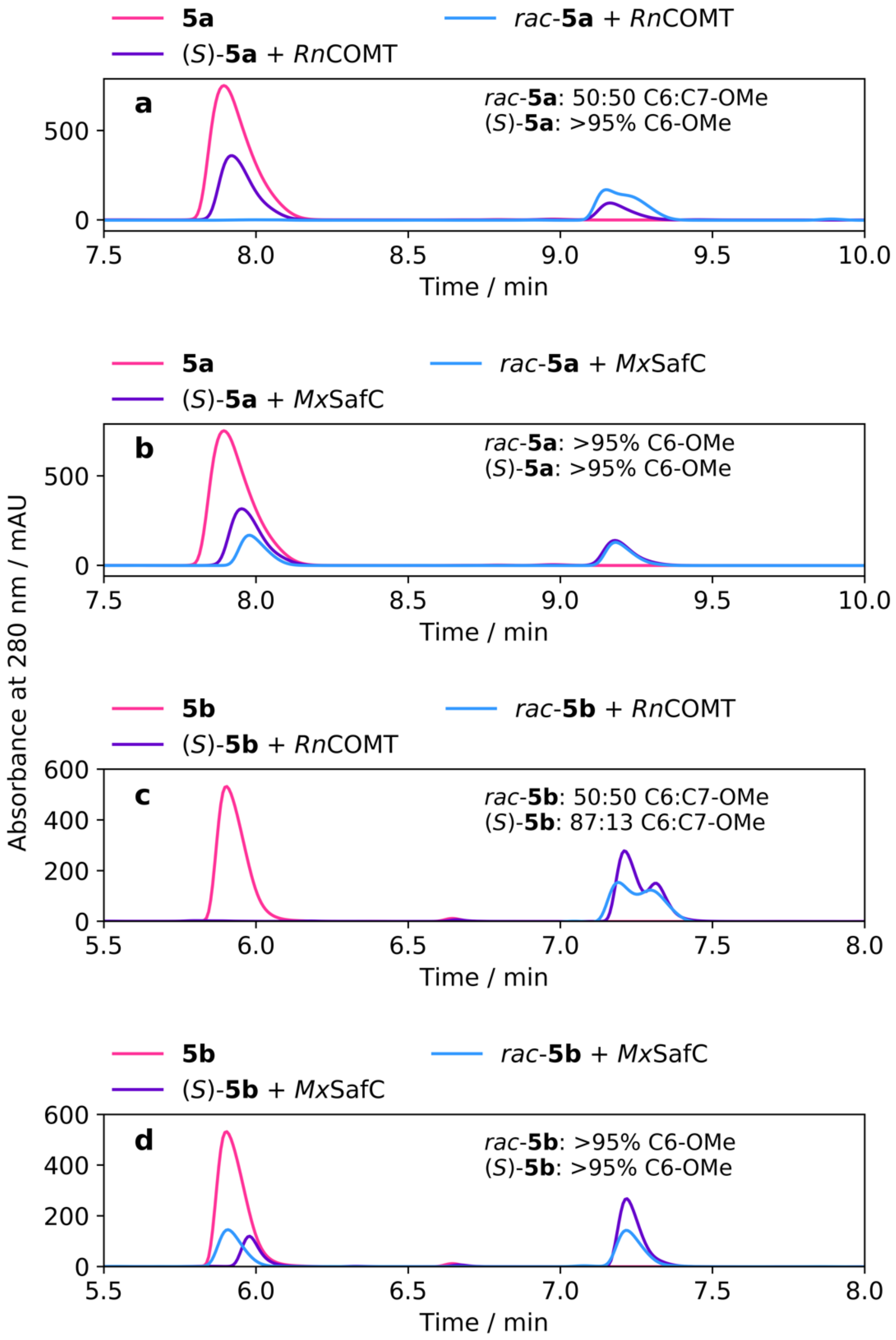

Supplementary Figure 27. RP-HPLC analysis of methyltransferase-catalysed reactions using **5a** or **5b** (either as the racemic or *S*-enantiomer, generated by a M97V-*Tf*NCS reaction) as the substrate. In all cases, traces **5a/b** correspond to the purified, isolated starting material. Reactions were then performed between *rac*/*S*-**5a/b** and *Mx*SafC or *Rn*COMT. RP-HPLC analysis of the final reaction mixture is shown, given with the ratios of *meta:para* methylated **7a/b** generated. a) Reactions between **5a** and *Rn*COMT. b) Reactions between **5a** and *Mx*SafC. c) Reactions between **5b** and *Rn*COMT. d) Reactions between **5b** and *MxSafC.*

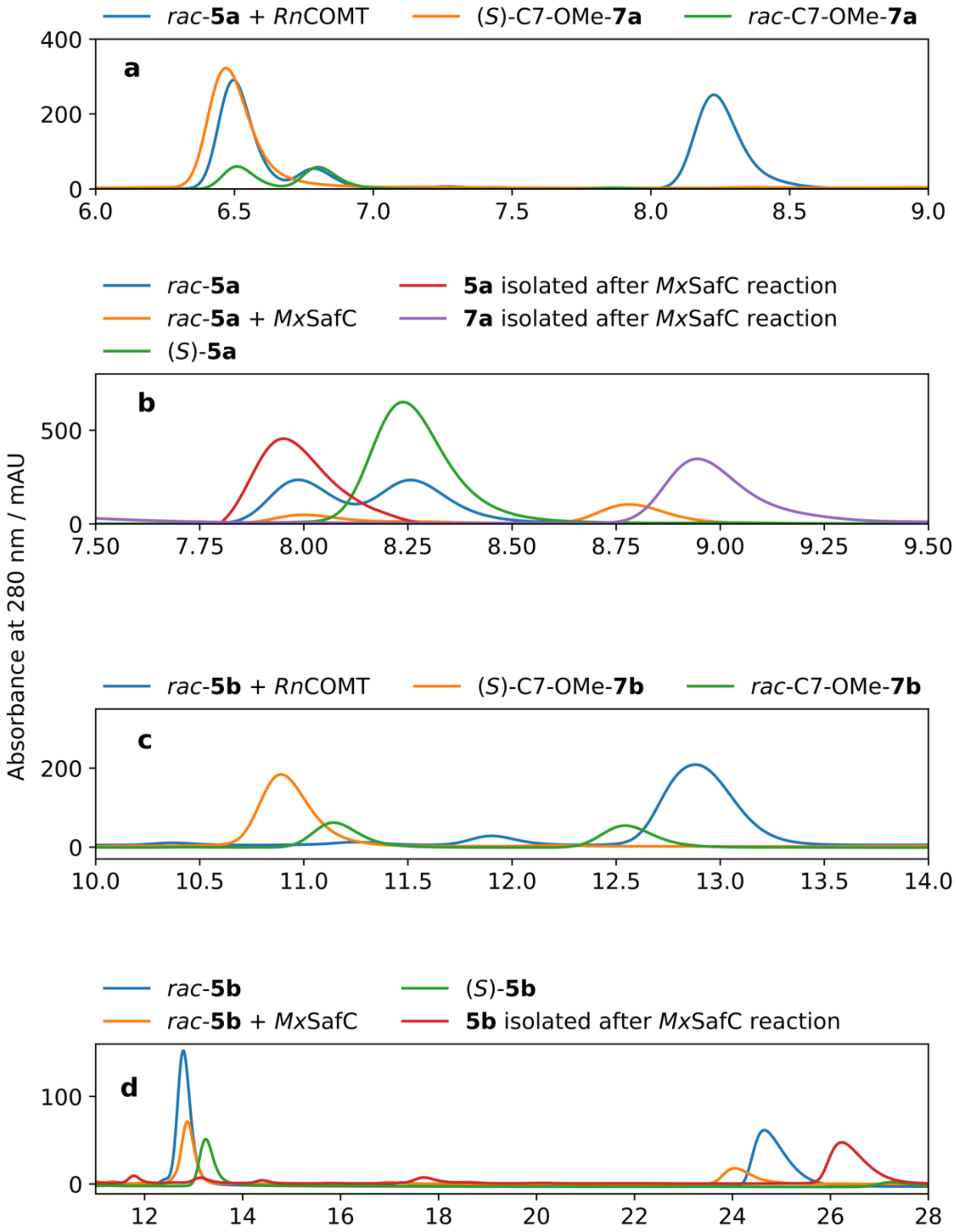

Supplementary Figure 28. Chiral HPLC analysis of methyltransferase reactions between **5a/b** (racemic) and *Rn*COMT and *Mx*SafC by chiral HPLC method 2. Products are compared to product standards. a) Isolated reaction product **7a** obtained from *Rn*COMT catalysed methylation of *rac*-**5a** compared with racemic and *S*-enantiomer standards of C7-OMe-**7a**. b) Analysis of the methylation of **5a** by *Mx*SafC; blue – standard of *rac*-**5a**, yellow – reaction mixture after methylation of *rac*-**5a**, green, standard of *S*-**5a**, red – isolation of **5a** after *Mx*SafC methylation, purple – isolation of **7a** generated by *Mx*SafC-catalysed methylation of **5a**. c) Isolated reaction product **7b** obtained from *Rn*COMT catalysed methylation of *rac*-**5a** compared with racemic and *S*-enantiomer standards of C7-OMe-**7b**. d) Analysis of the methylation of **5b** by *Mx*SafC; blue – standard of *rac*-**5b**, yellow – reaction mixture after methylation of *rac*-**5b**, green – standard of *S*-**5b**, red – **5b** isolated after *Mx*SafC reaction.

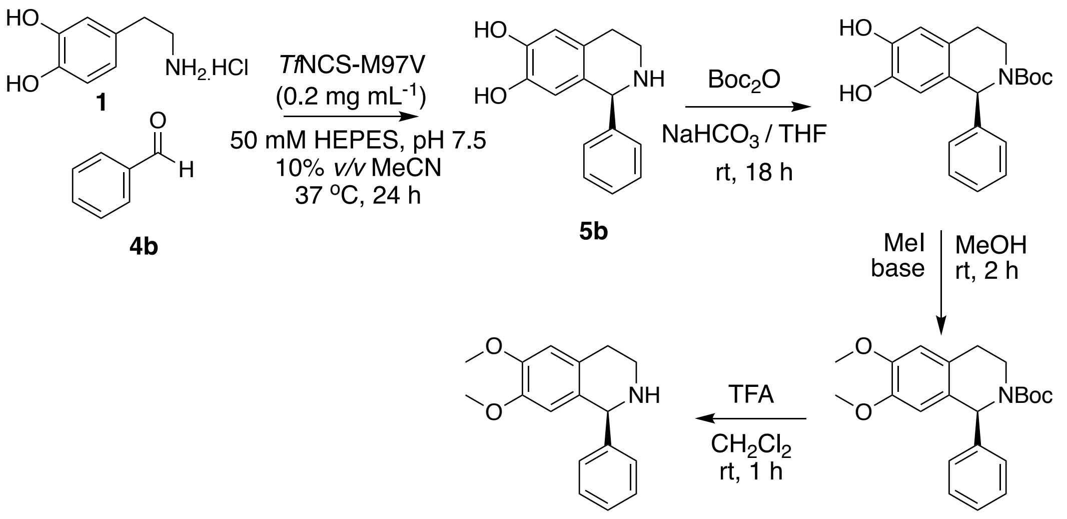

Supplementary Figure 29. Synthetic route to give (1*S*)-6,7-dimethoxy-1-phenyl-1,2,3,4-tetrahydroisoquinoline via a stereoselective NCS-mediated Pictet-Spengler reaction.

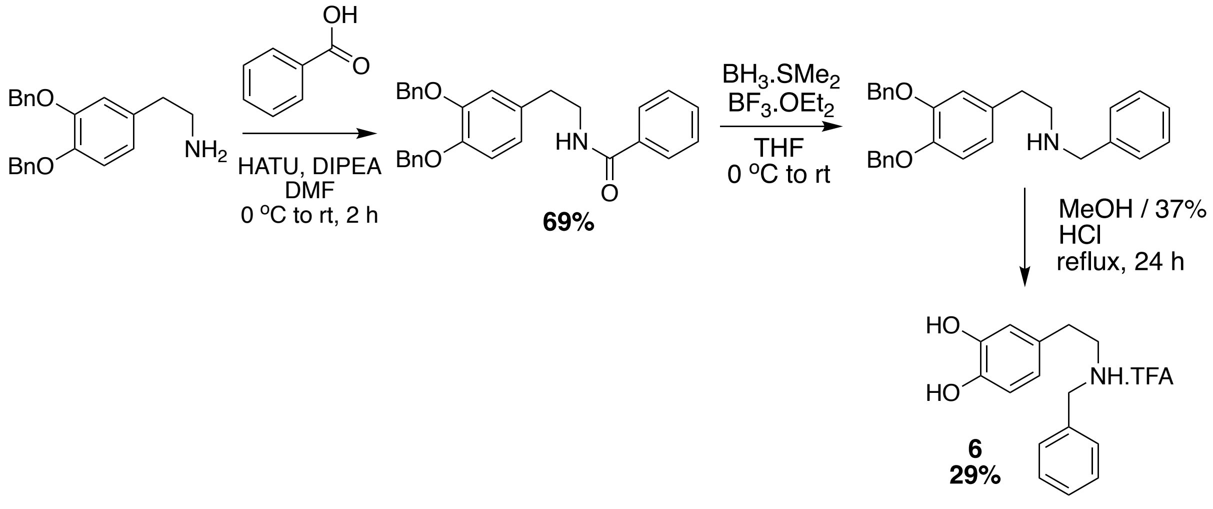

Supplementary Figure 30. Synthetic route towards reaction intermediate mimic, **6.**

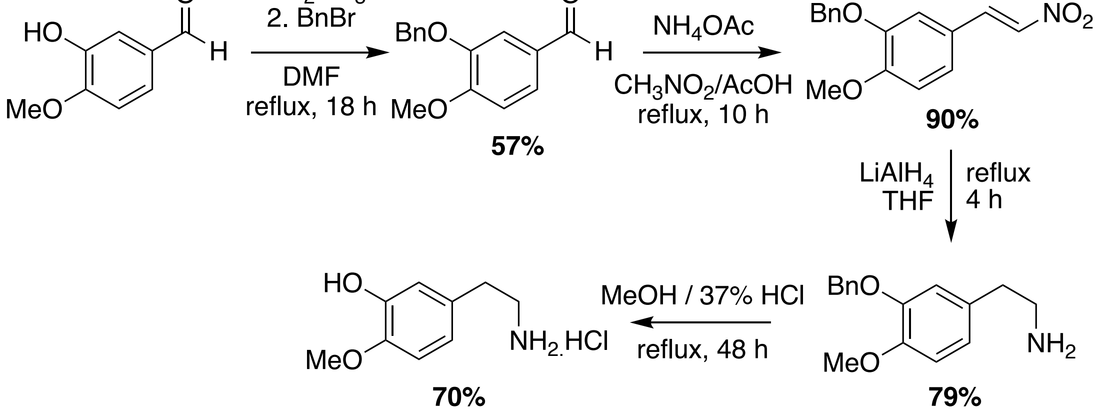

Supplementary Figure 31. Synthetic route towards 4-methoxytyramine hydrochloride.

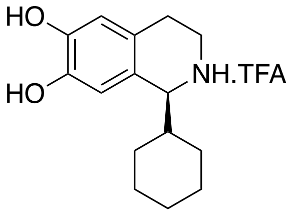

^{1}H-NMR (700 MHz)

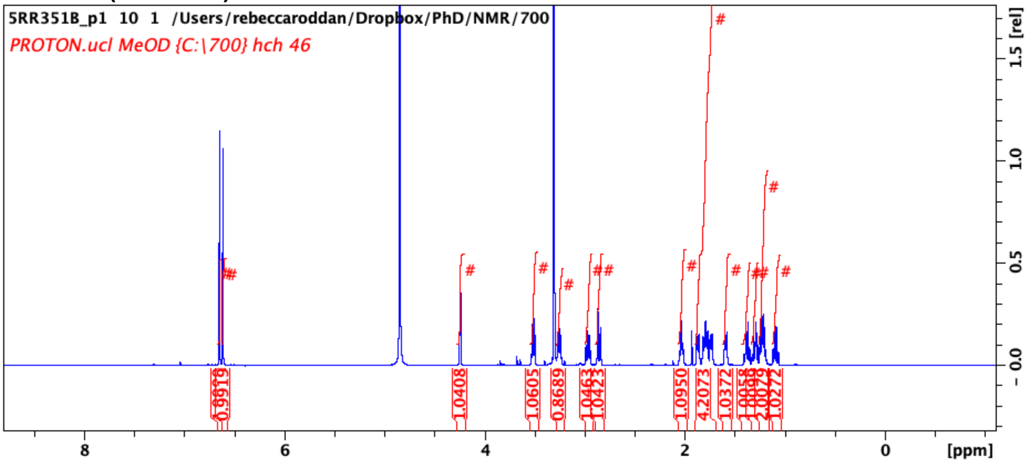

^{1}H-NMR (700 MHz): 5.0 – 2.5 ppm

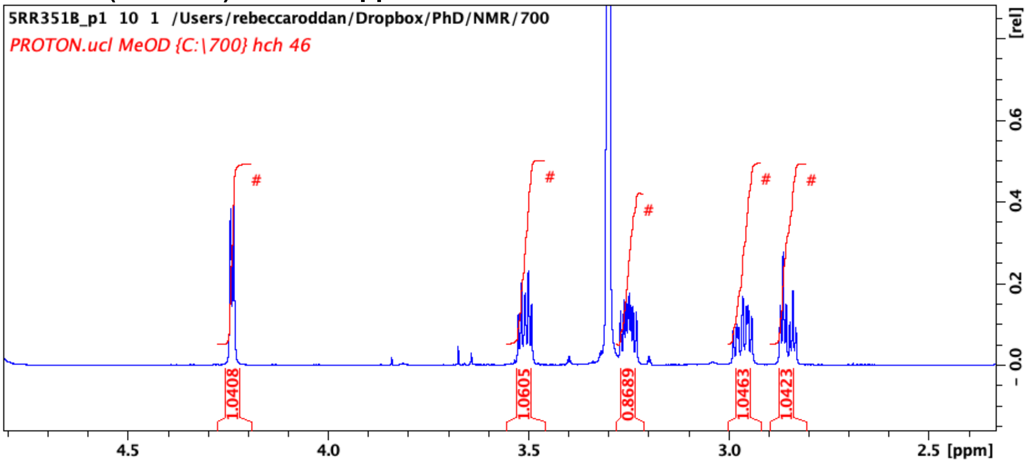

^{1}H-NMR (700 MHz): 3.0 – 0.5 ppm

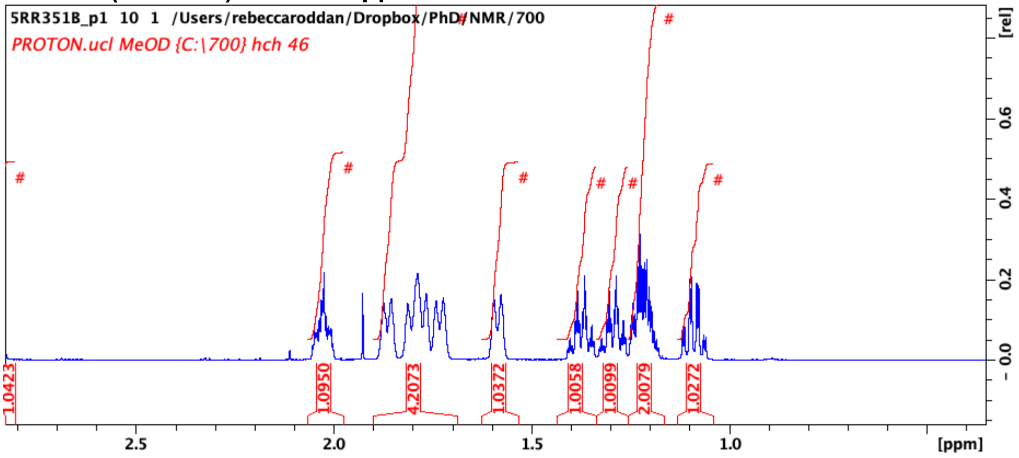

Supplementary Figure 32. ^{1}H-NMR spectra of (1*S*)-1-cyclohexyl-1,2,3,4-tetrahydroisoquinoline-6,7-diol (**5a**)

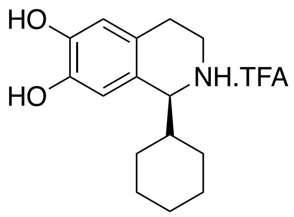

^{13}C-NMR (176 MHz)

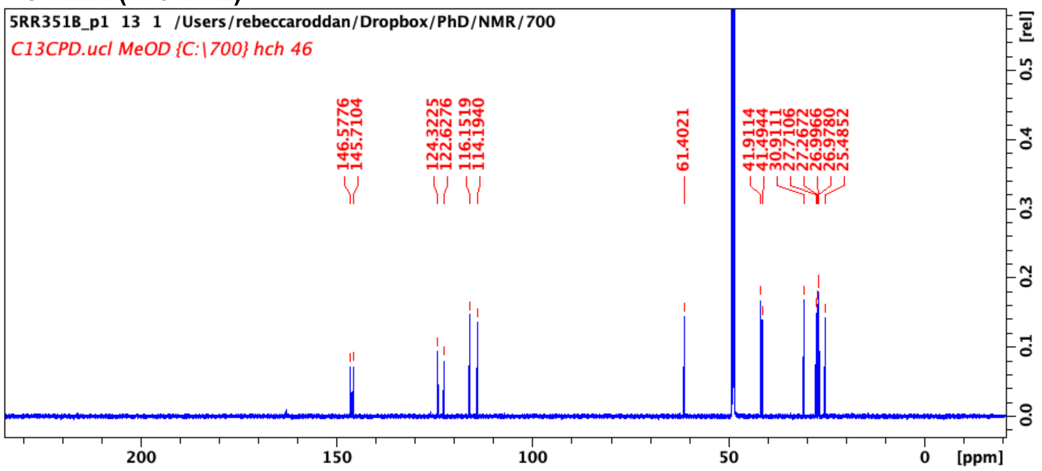

^{13}C-NMR (176 MHz): 25.3 – 28 ppm

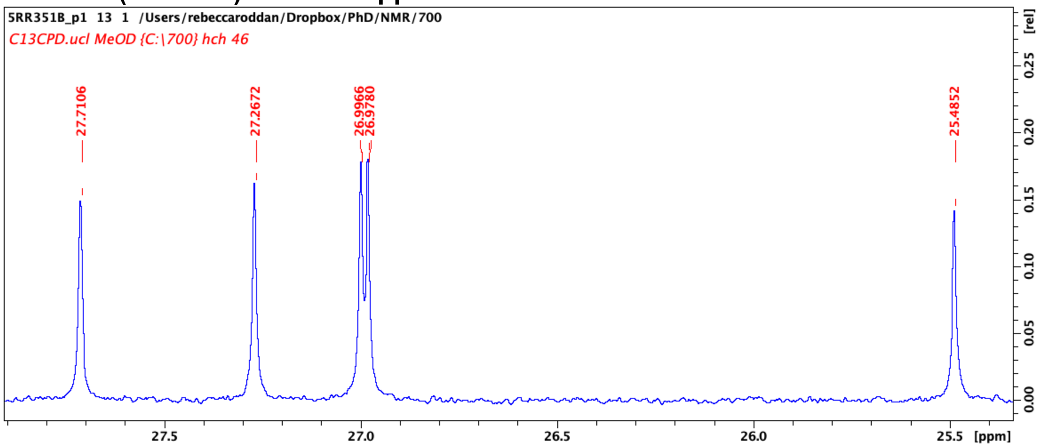

Supplementary Figure 33. ^{13}C-NMR spectra of (1*S*)-1-cyclohexyl-1,2,3,4-tetrahydroisoquinoline-6,7-diol (**5a**)

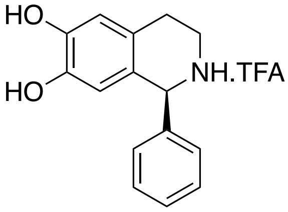

^{1}H-NMR (700 MHz)

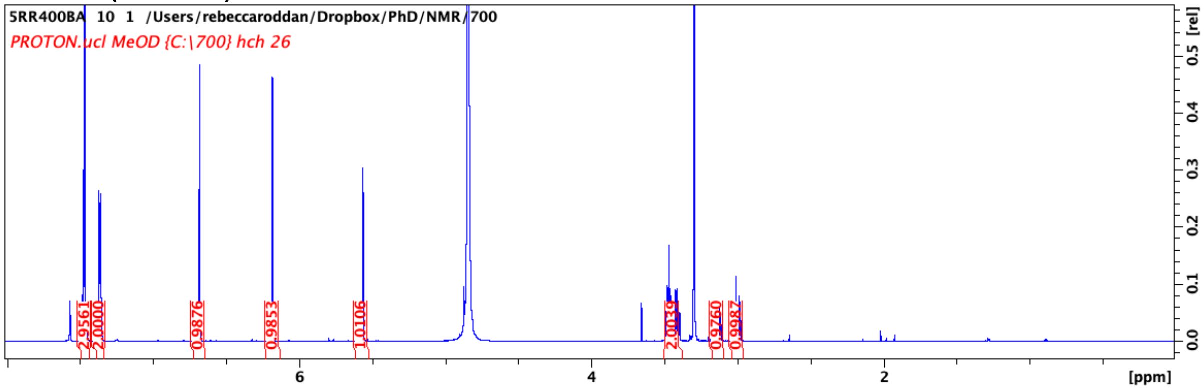

^{1}H-NMR (700 MHz): 7.6 – 5.3 ppm

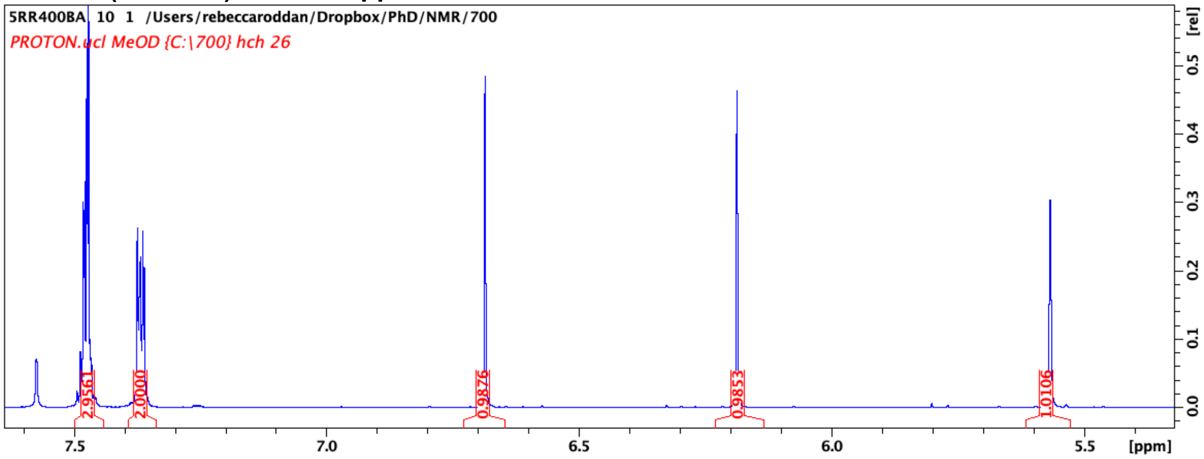

^{1}H-NMR (700 MHz): 3.6 – 2.9 ppm

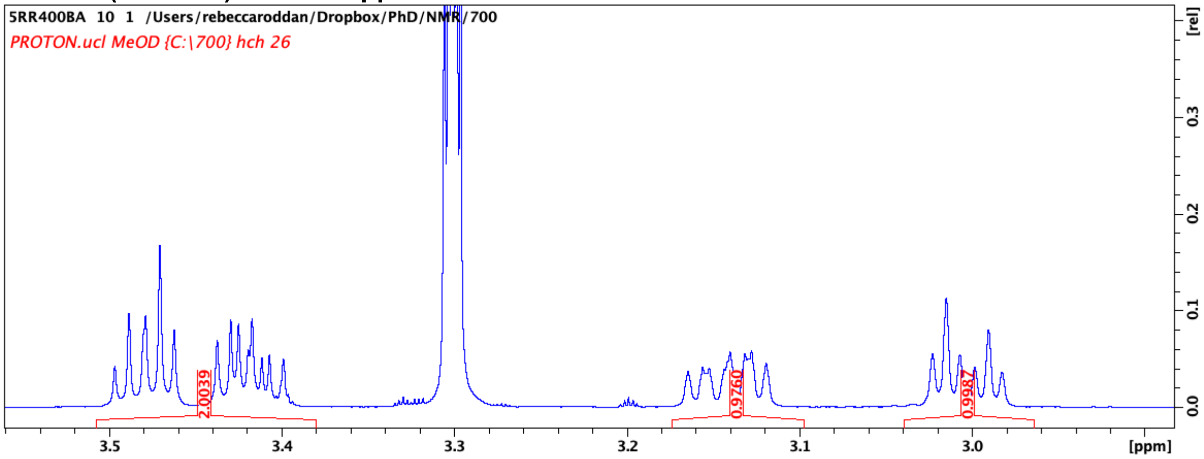

Supplementary Figure 34. ^{1}H-NMR spectra of (1*S*)-1-phenyl-1,2,3,4-tetrahydroisoquinoline-6,7-diol (**5b**)

^{13}C-NMR (176 MHz)

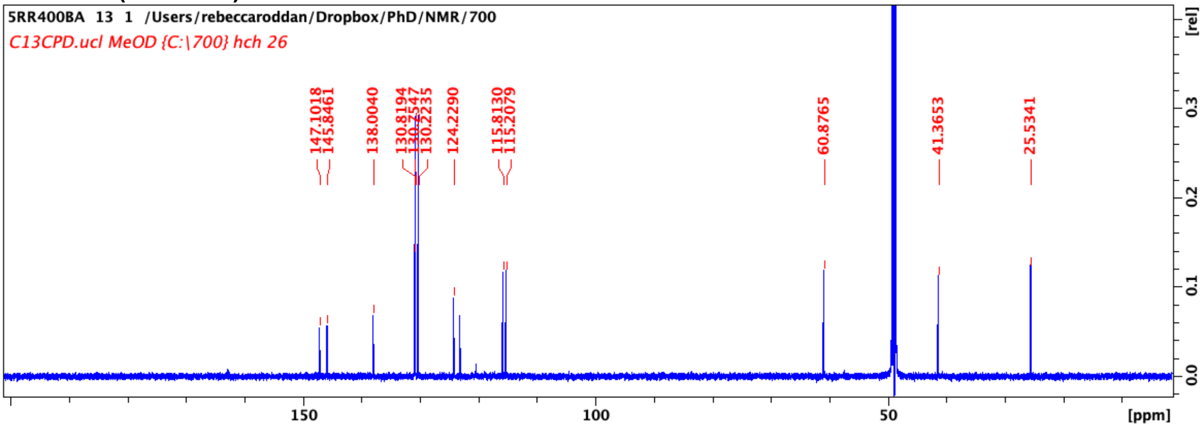

Supplementary Figure 35. ^{13}C-NMR spectrum of (1*S*)-1-phenyl-1,2,3,4-tetrahydroisoquinoline-6,7-diol (**5b**)

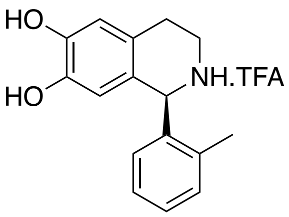

^{1}H-NMR (700 MHz)

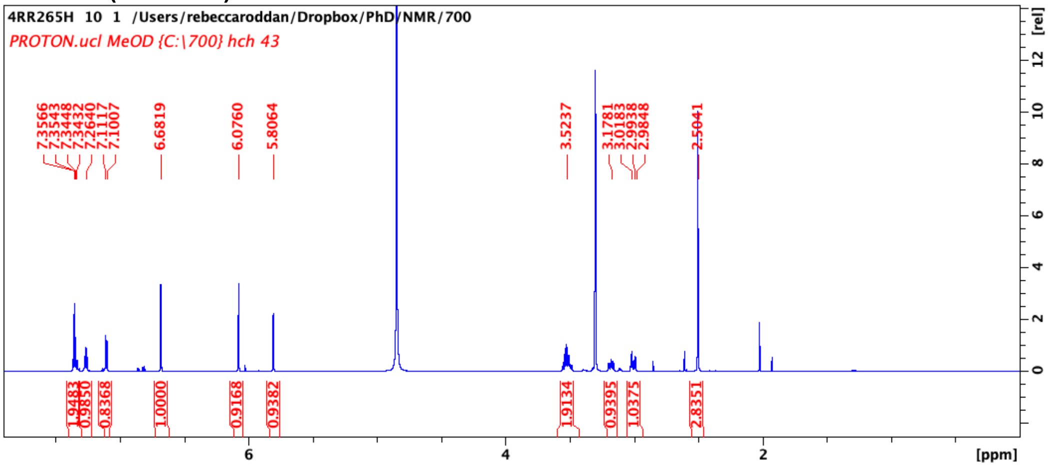

^{1}H-NMR (700 MHz): 7.5 – 5.5 ppm

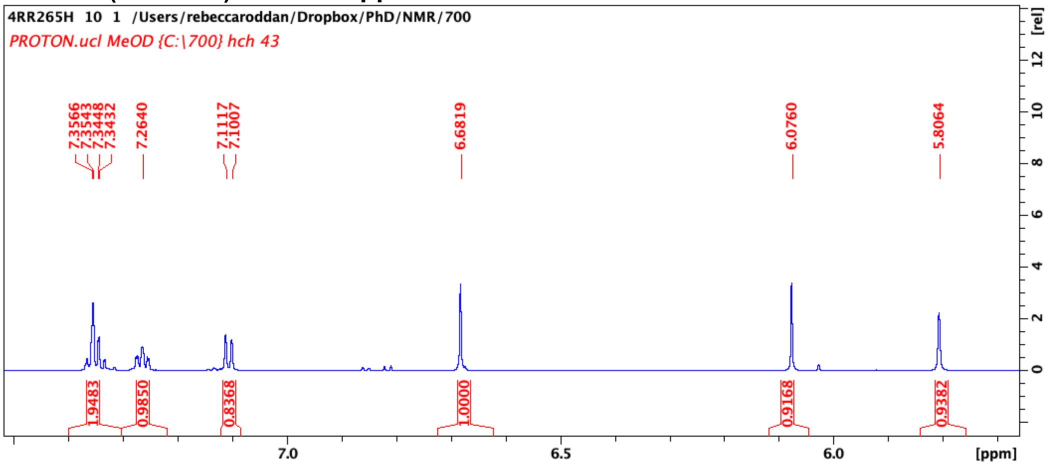

^{1}H-NMR (700 MHz): 3.6 – 2.4 ppm

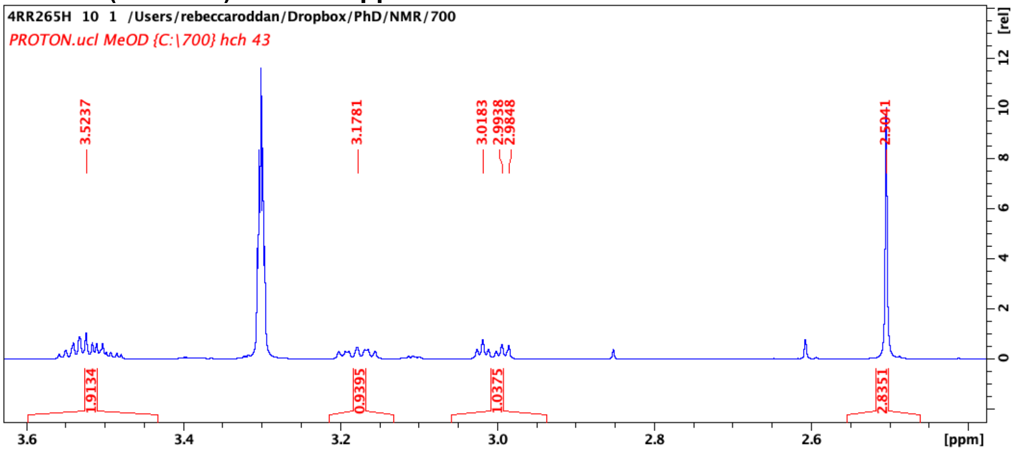

Supplementary Figure 36. ^{1}H-NMR spectra of (1*S*)-1-(*o*-tolyl)-1,2,3,4-tetrahydroisoquinoline-6,7-diol (**5c**)

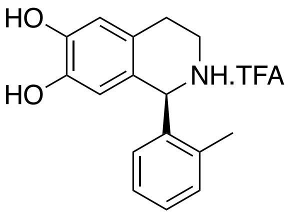

^{13}C-NMR (176 MHz)

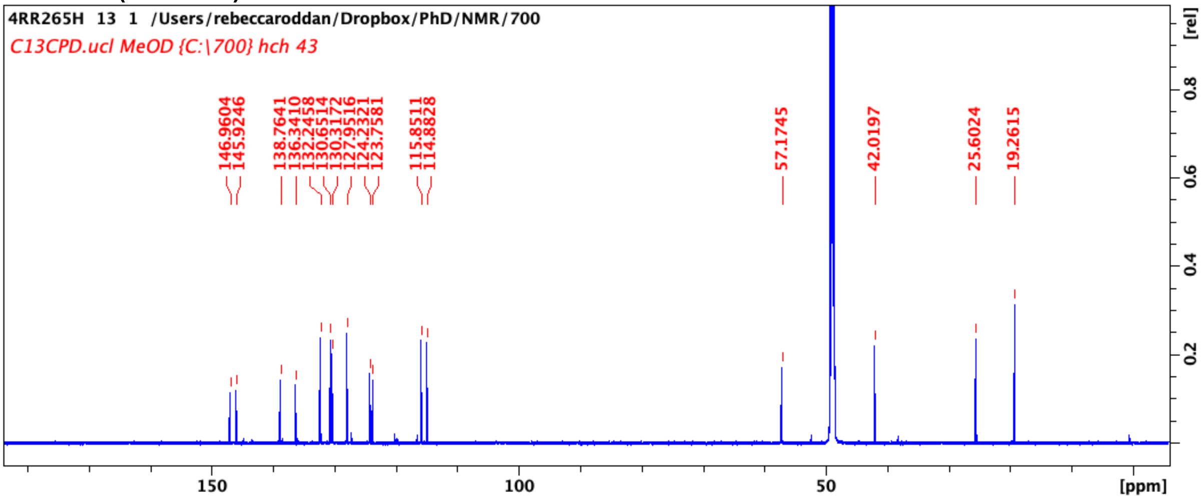

Supplementary Figure 37. ^{13}C-NMR spectrum of (1*S*)-1-(*o*-tolyl)-1,2,3,4-tetrahydroisoquinoline-6,7-diol (**5c**).

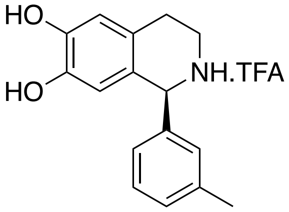

^{1}H-NMR (700 MHz)

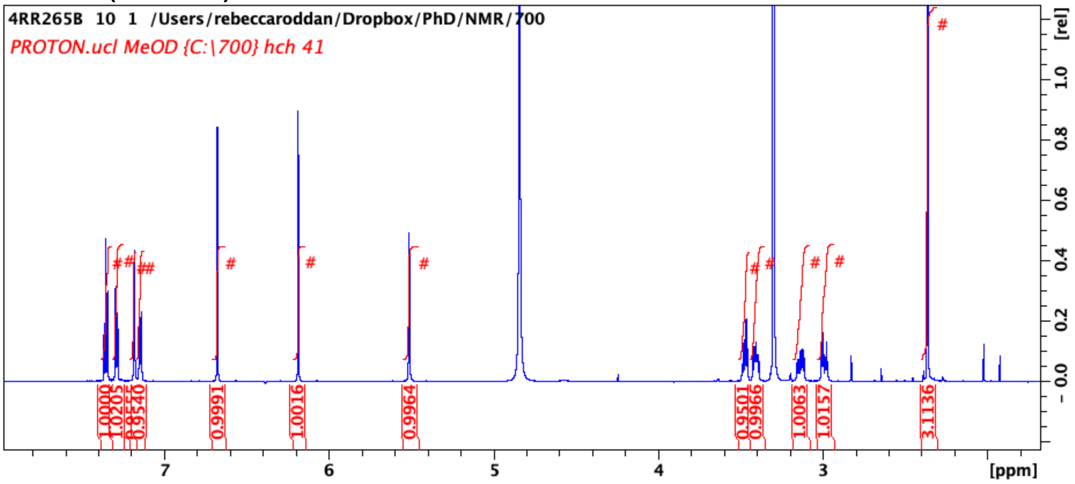

^{1}H-NMR (700 MHz): 8.0 – 5.0 ppm

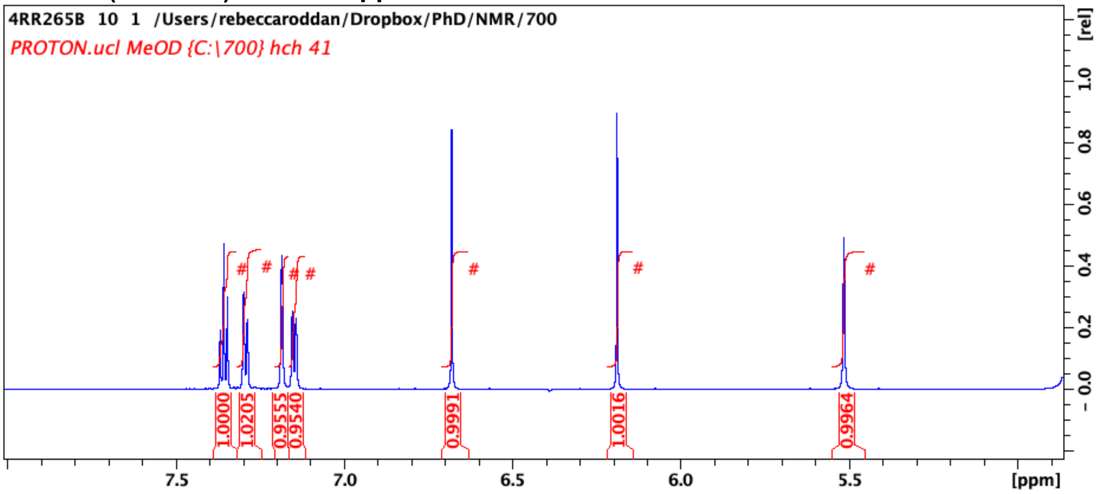

^{1}H-NMR (700 MHz): 4.5 – 1.4 ppm

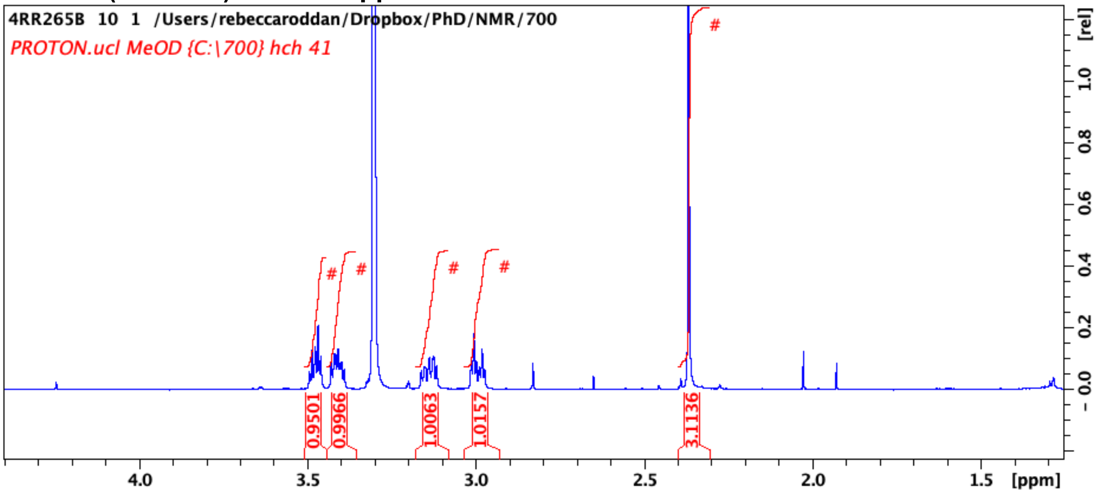

Supplementary Figure 38. ^{1}H-NMR spectra of (1*S*)-1-(*m*-tolyl)-1,2,3,4-tetrahydroisoquinoline-6,7-diol (**5d**)

^{13}C-NMR (176 MHz)

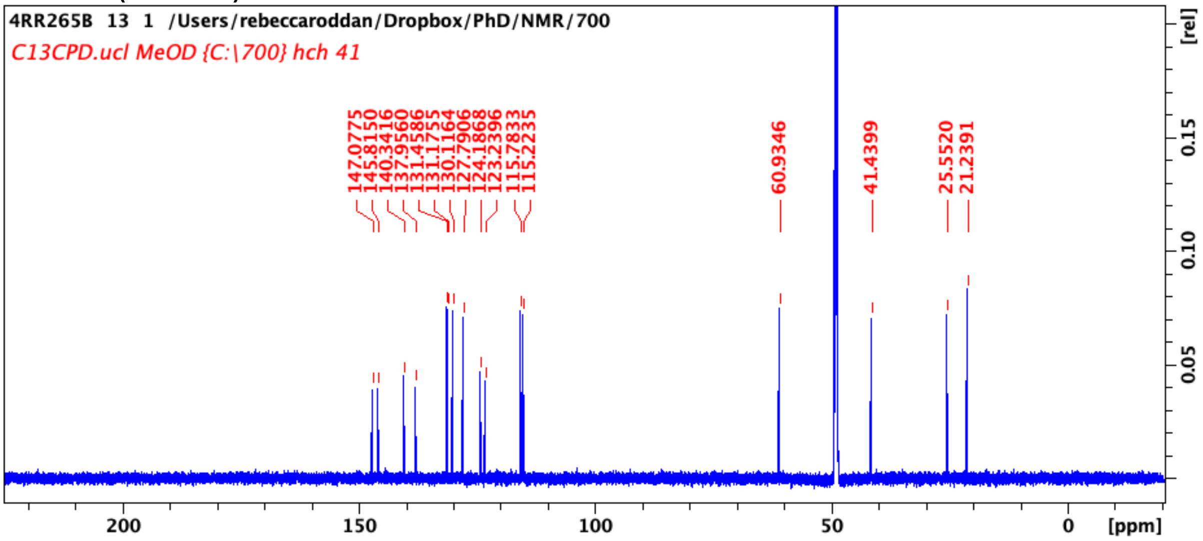

Supplementary Figure 39. ^{13}C-NMR spectra of (1*S*)-1-(*m*-tolyl)-1,2,3,4-tetrahydroisoquinoline-6,7-diol (**5d**).

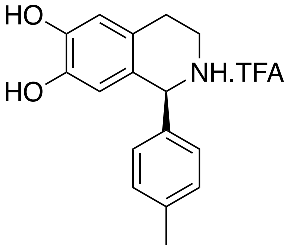

^{1}H-NMR (700 MHz)

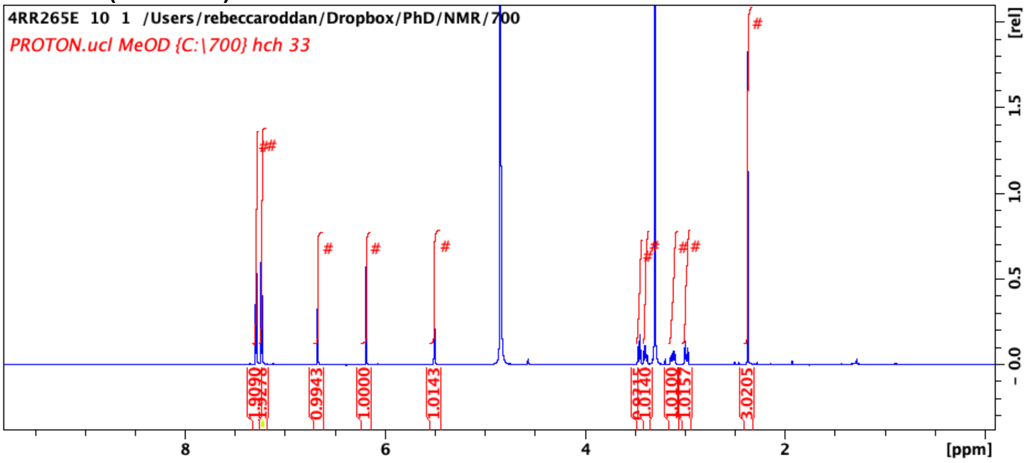

^{1}H-NMR (700 MHz): 7.5 – 5.3 ppm

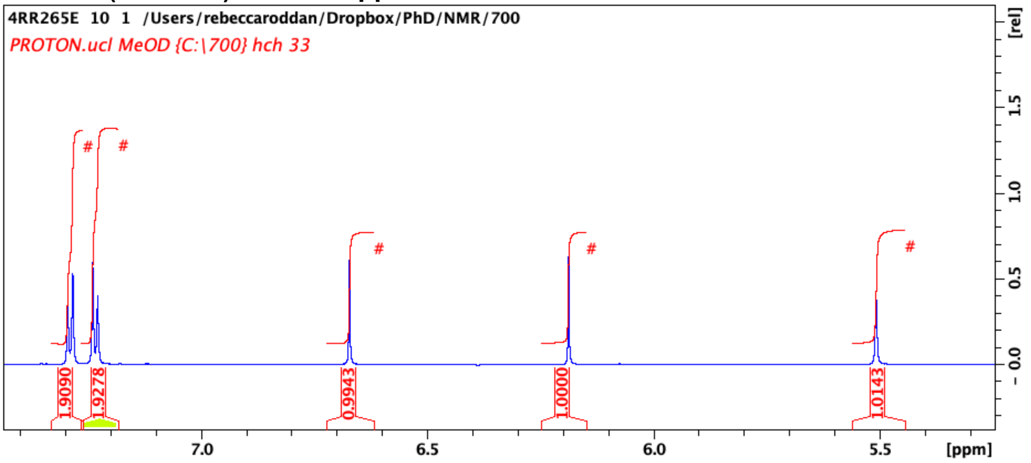

^{1}H-NMR (700 MHz): 3.6 – 2.2 ppm

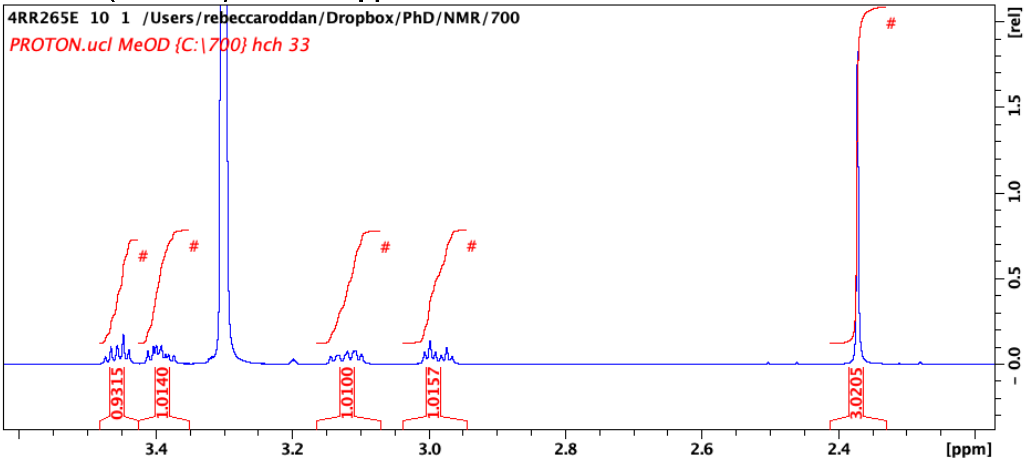

Supplementary Figure 40. ^{1}H-NMR spectra of (1*S*)-1-(*p*-tolyl)-1,2,3,4-tetrahydroisoquinoline-6,7-diol (**5e**)

^{13}C-NMR (176 MHz)

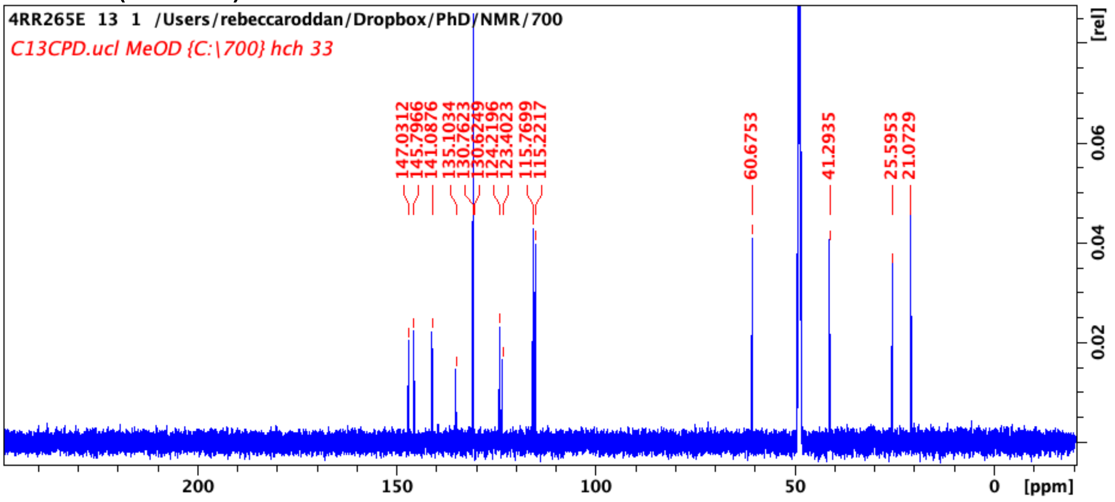

Supplementary Figure 41. ^{13}C-NMR spectrum of (1*S*)-1-(*p*-tolyl)-1,2,3,4-tetrahydroisoquinoline-6,7-diol (**5e**).

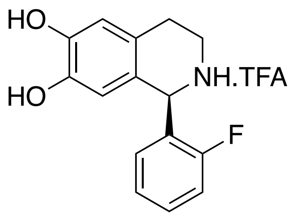

^{1}H-NMR (700 MHz)

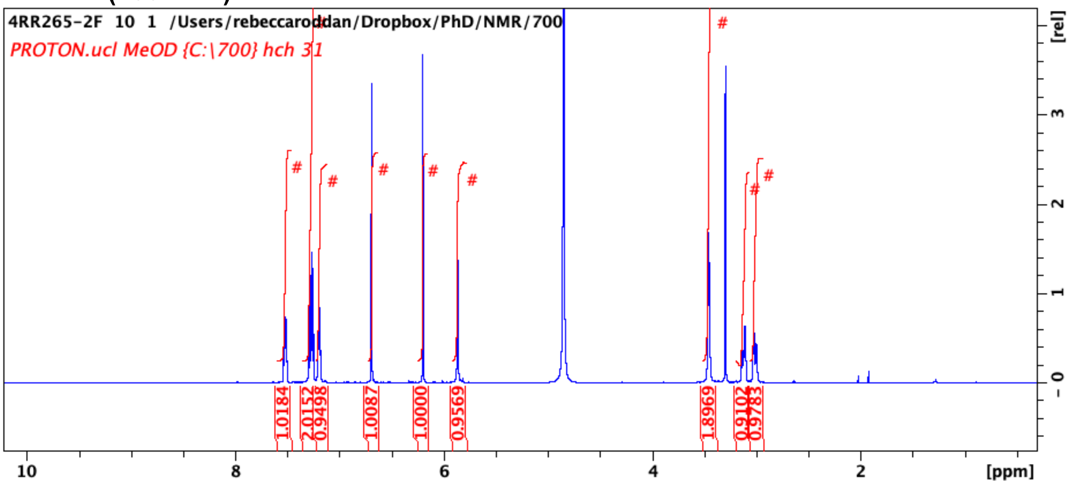

^{1}H-NMR (700 MHz): 8.0 – 5.5 ppm

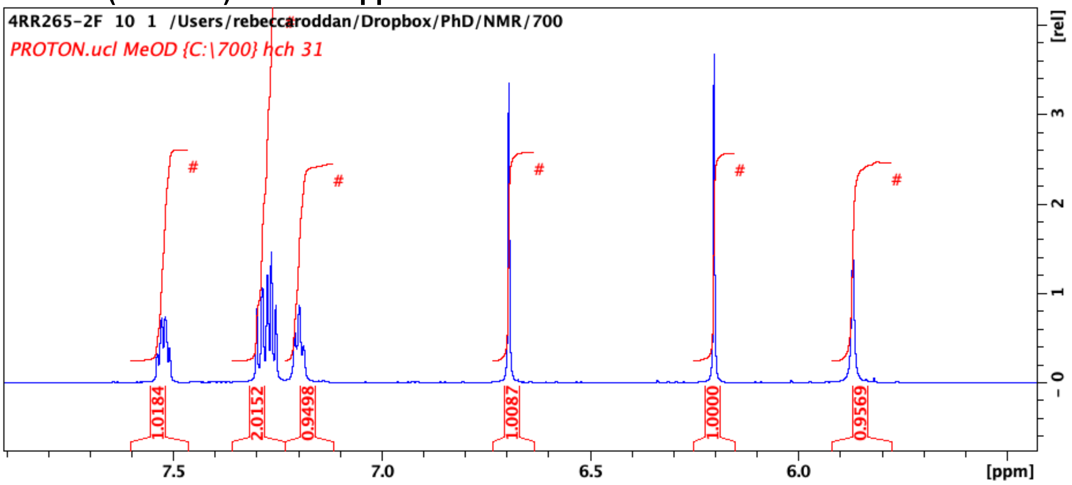

^{1}H-NMR (700 MHz): 3.8 – 2.6 ppm

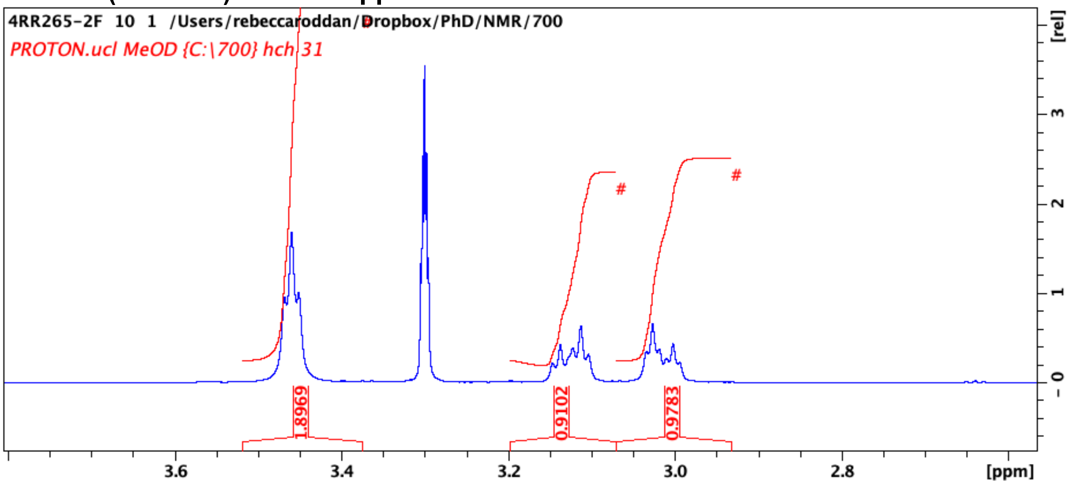

Supplementary Figure 42. ^{1}H-NMR spectra of (1*R*)-1-(2-fluorophenyl)-1,2,3,4-tetrahydroisoquinoline-6,7-diol (**5f**)

^{13}C-NMR (176 MHz)

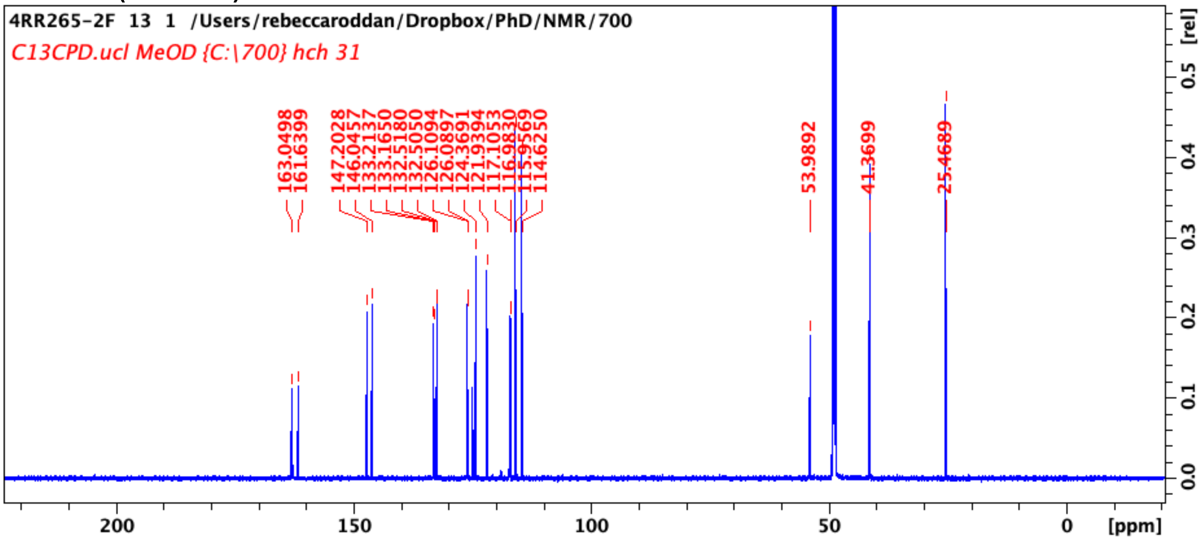

Supplementary Figure 43. ^{13}C-NMR spectrum of (1*R*)-1-(2-fluorophenyl)-1,2,3,4-tetrahydroisoquinoline-6,7-diol (**5f**)

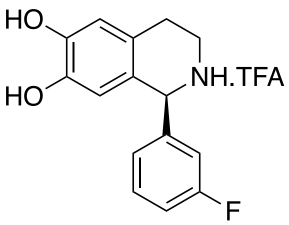

^{1}H-NMR (700 MHz)

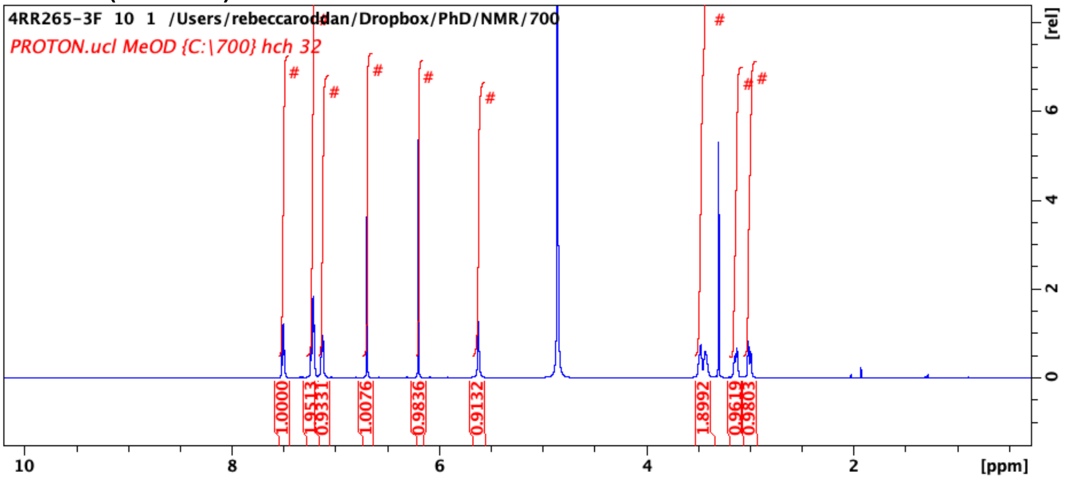

^{1}H-NMR (700 MHz): 8.0 – 5.5 ppm

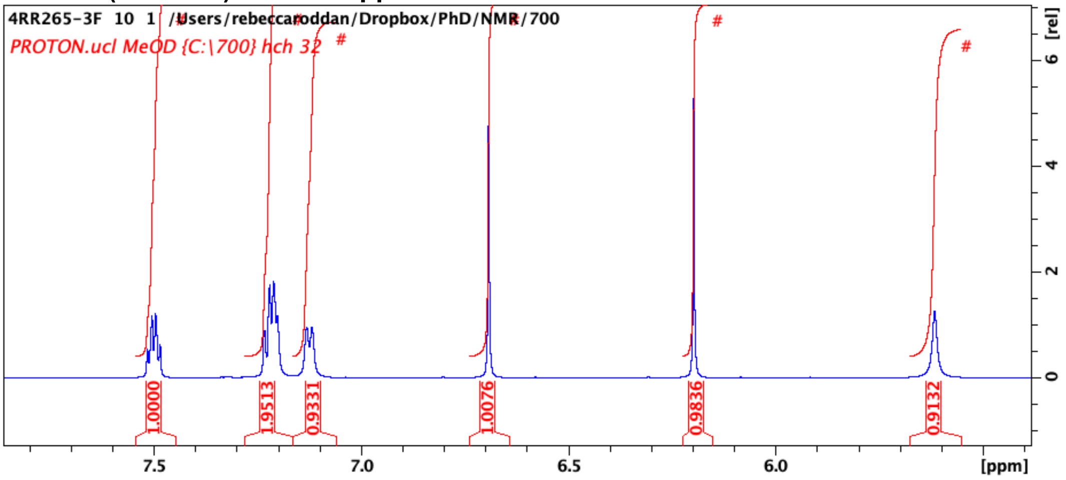

^{1}H-NMR (700 MHz): 3.9 – 2.6 ppm

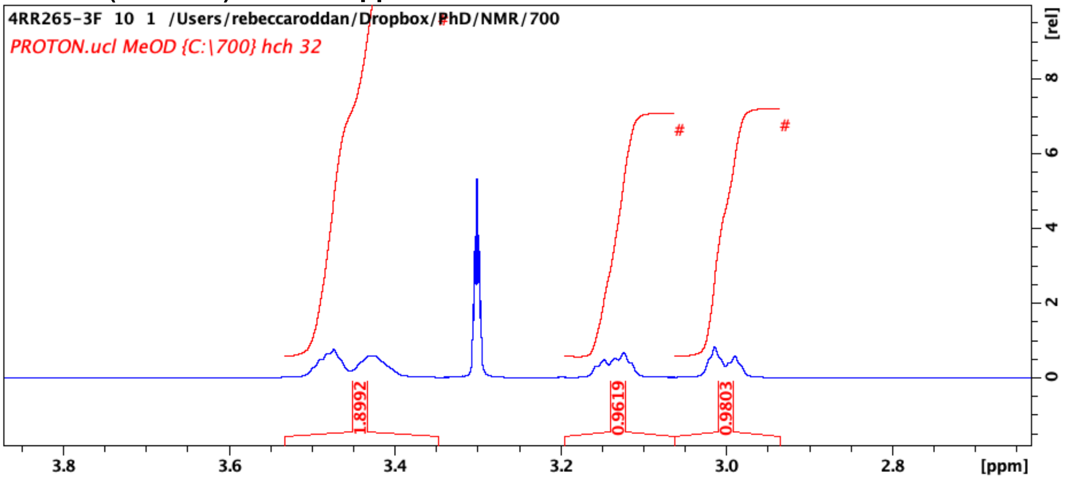

Supplementary Figure 44. ^{1}H-NMR spectra of (1*S*)-1-(3-fluorophenyl)-1,2,3,4-tetrahydroisoquinoline-6,7-diol (**5g**).

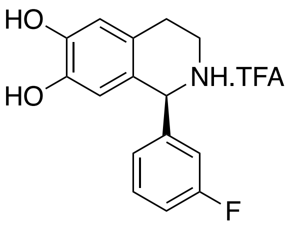

^{13}C-NMR (176 MHz)

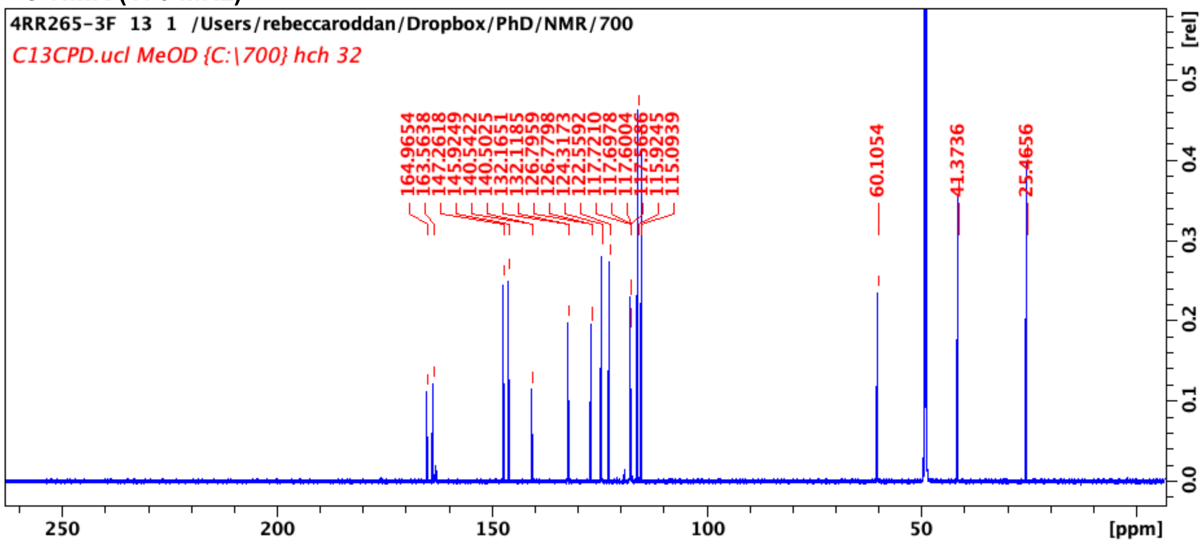

Supplementary Figure 45. ^{13}C-NMR spectrum of (1*S*)-1-(3-fluorophenyl)-1,2,3,4-tetrahydroisoquinoline-6,7-diol (**5g**).

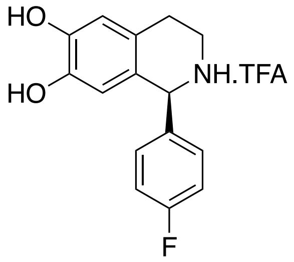

^{1}H-NMR (700 MHz)

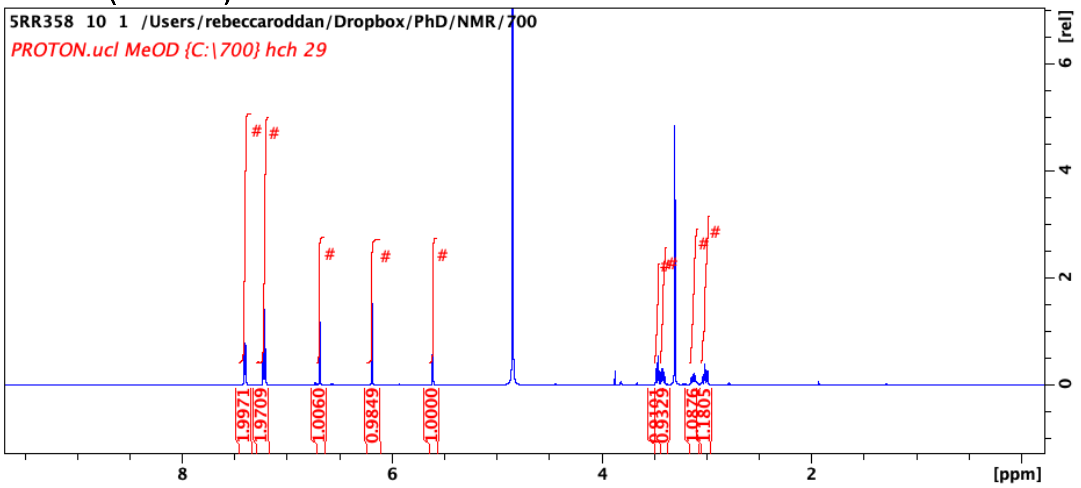

^{1}H-NMR (700 MHz): 7.5 – 5.5 ppm

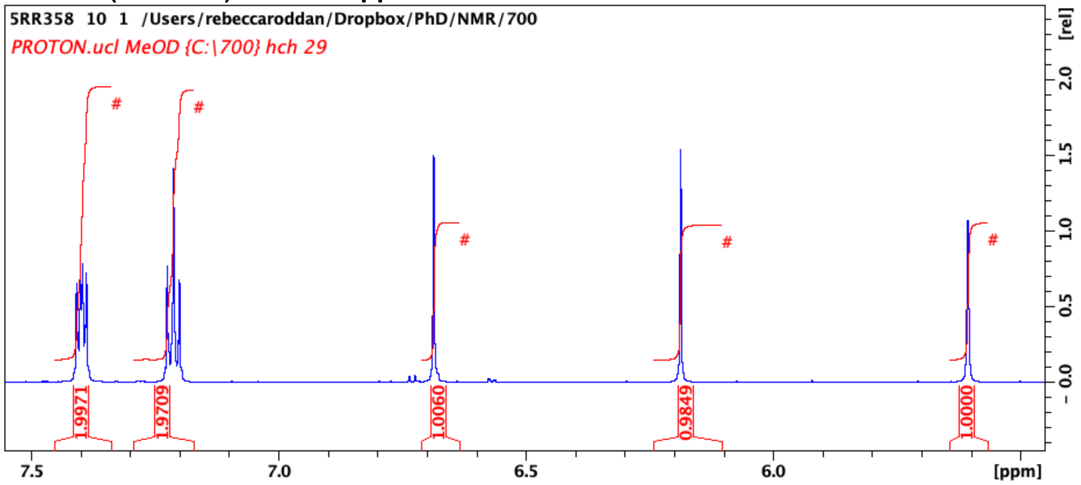

^{1}H-NMR (700 MHz): 3.6 – 2.9 ppm

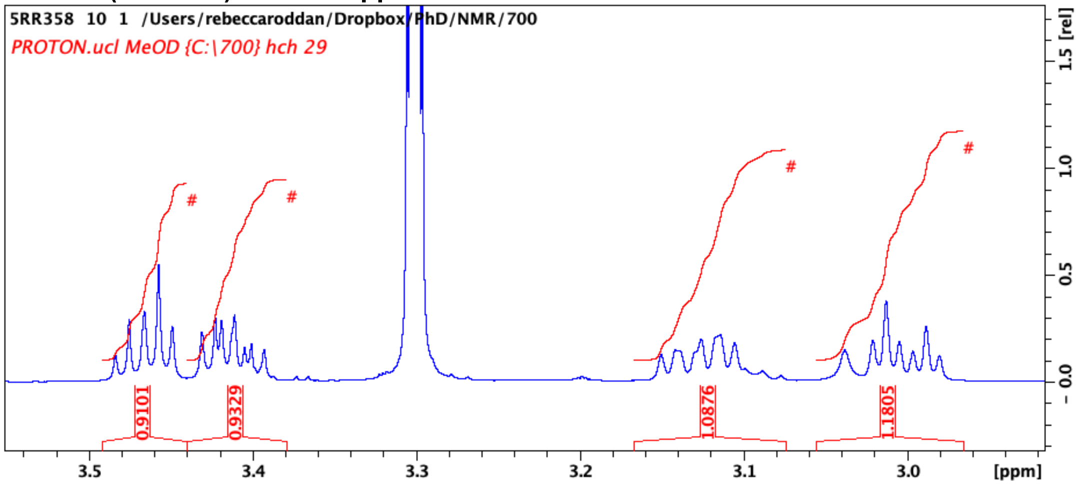

Supplementary Figure 46. ^{1}H-NMR spectra of (1*S*)-1-(4-fluorophenyl)-1,2,3,4-tetrahydroisoquinoline-6,7-diol (**5h**).

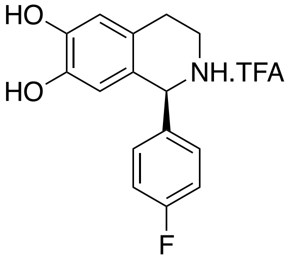

^{13}C-NMR (176 MHz)

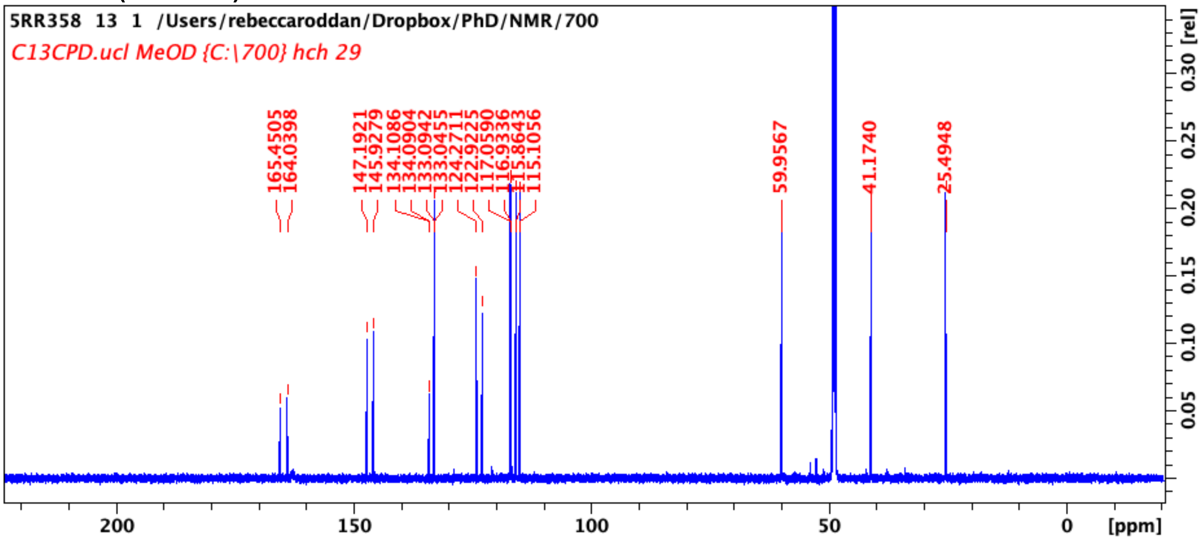

Supplementary Figure 47. ^{13}C-NMR spectrum of (1*S*)-1-(4-fluorophenyl)-1,2,3,4-tetrahydroisoquinoline-6,7-diol (**5h**).

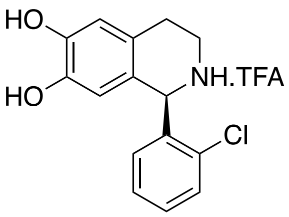

^{1}H-NMR (700 MHz)

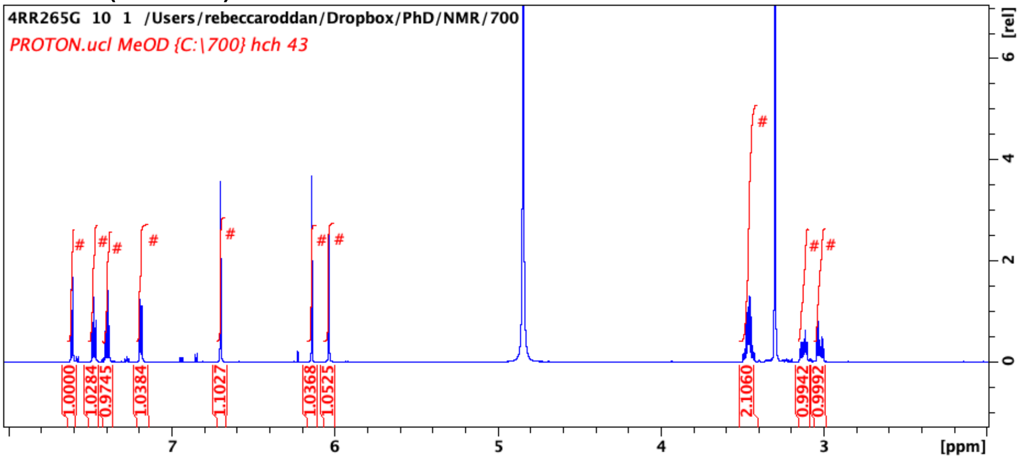

^{1}H-NMR (700 MHz): 7.7 – 5.9 ppm

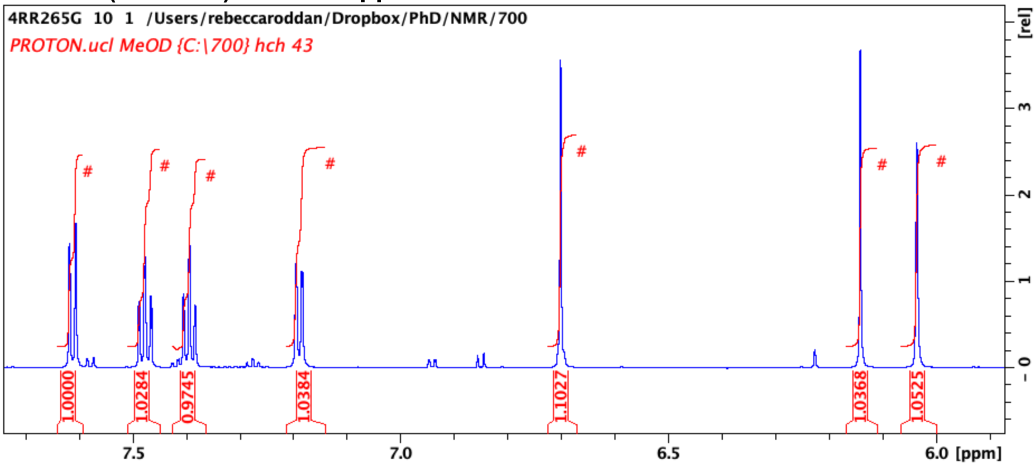

^{1}H-NMR (700 MHz): 3.8 – 2.8 ppm

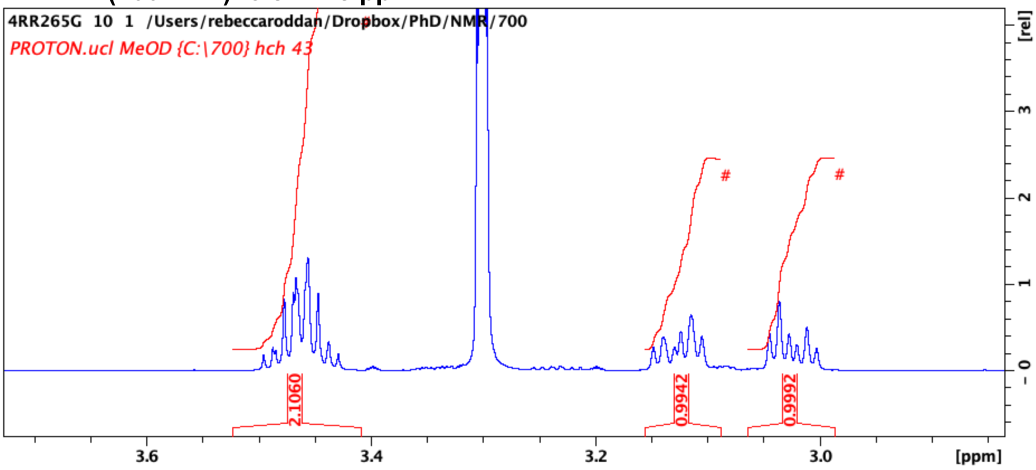

Supplementary Figure 48. ^{1}H-NMR spectra of (1*R*)-1-(2-chlorophenyl)-1,2,3,4-tetrahydroisoquinoline-6,7-diol (**5i**).

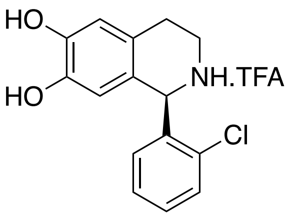

^{13}C-NMR (176 MHz)

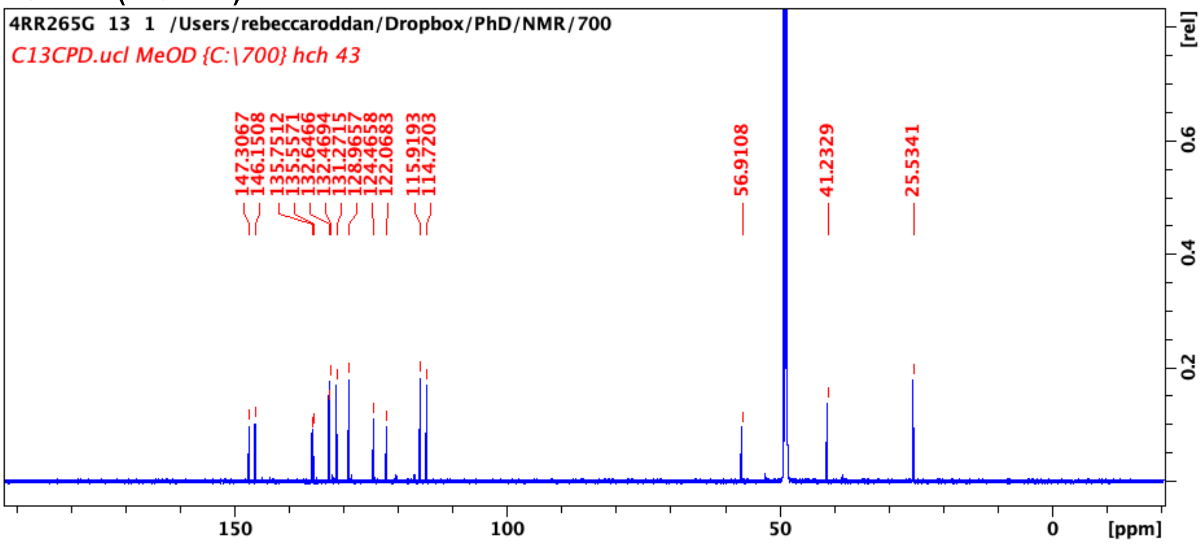

Supplementary Figure 49. ^{13}C-NMR spectrum of (1*R*)-1-(2-chlorophenyl)-1,2,3,4-tetrahydroisoquinoline-6,7-diol (**5i**).

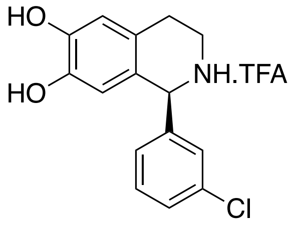

^{1}H-NMR (700 MHz)

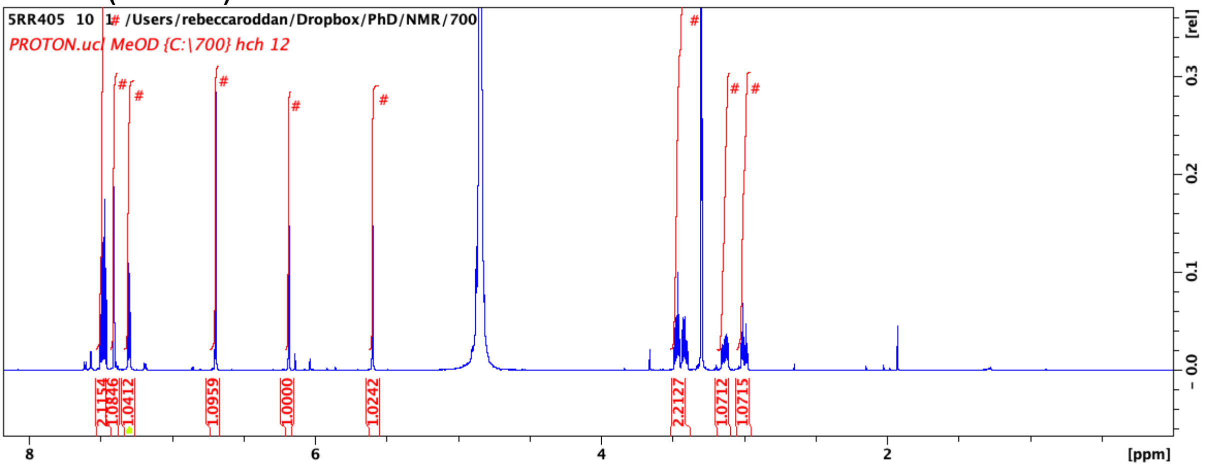

^{1}H-NMR (700 MHz): 7.5 – 5.5 ppm

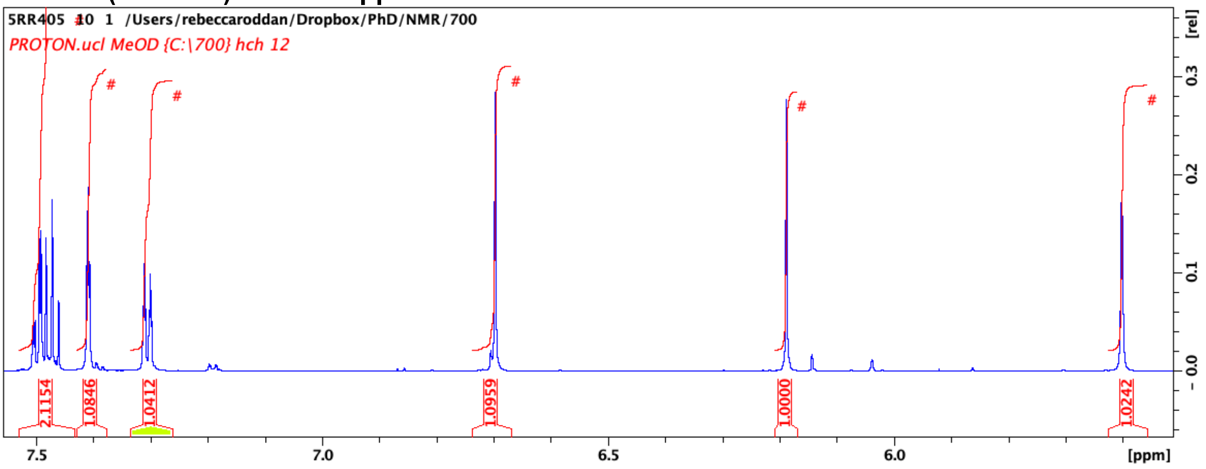

^{1}H-NMR (700 MHz): 3.6 – 2.8 ppm

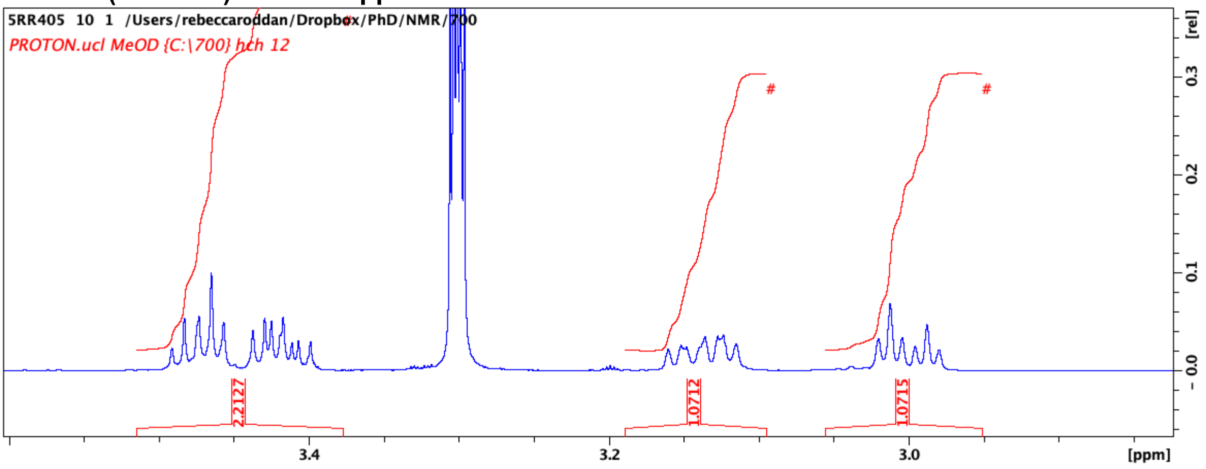

Supplementary Figure 50. ^{1}H-NMR spectra of (1*S*)-1-(3-Chlorophenyl)-1,2,3,4-tetrahydroisoquinoline-6,7-diol (**5j**).

^{13}C-NMR (176 MHz)

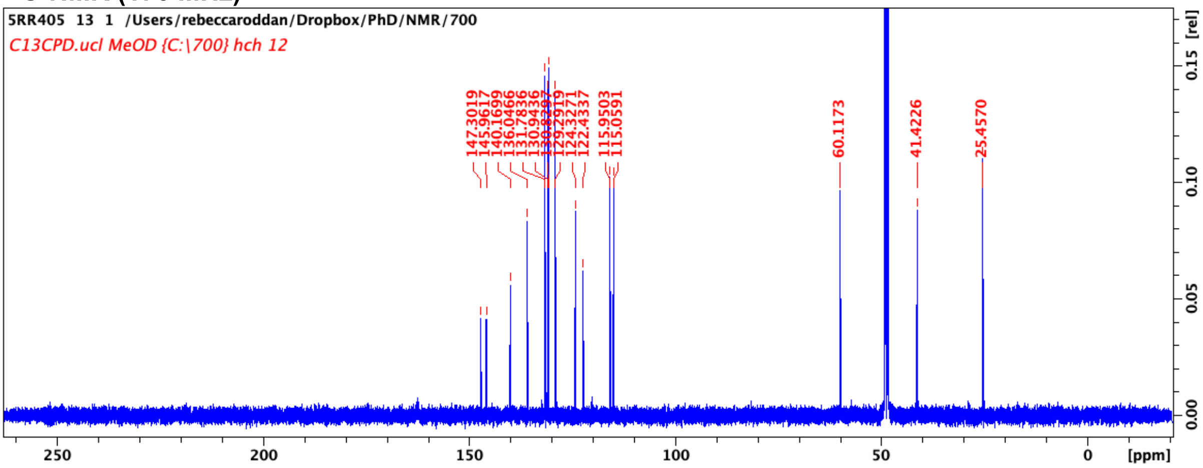

Supplementary Figure 51. ^{13}C-NMR spectrum of (1*S*)-1-(3-chlorophenyl)-1,2,3,4-tetrahydroisoquinoline-6,7-diol (**5j**).

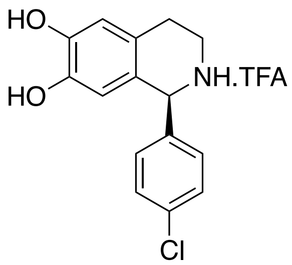

^{1}H-NMR (700 MHz)

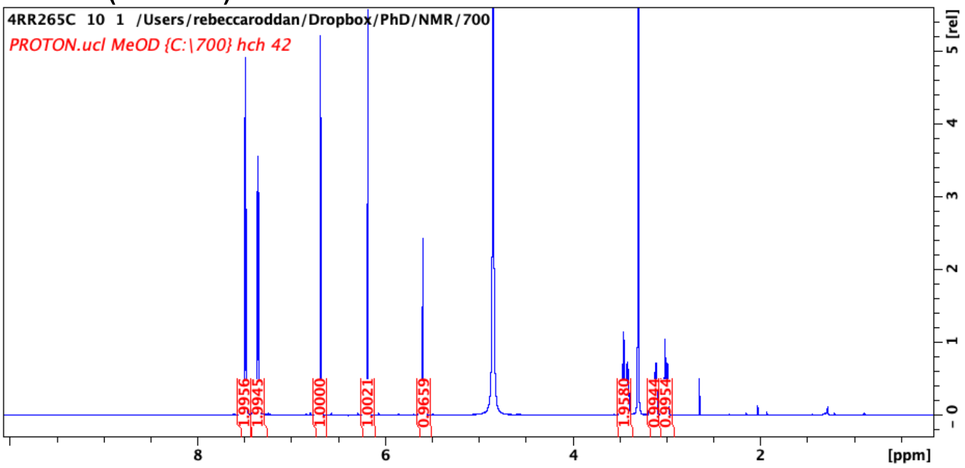

^{1}H-NMR (700 MHz): 7.7 – 5.5 ppm

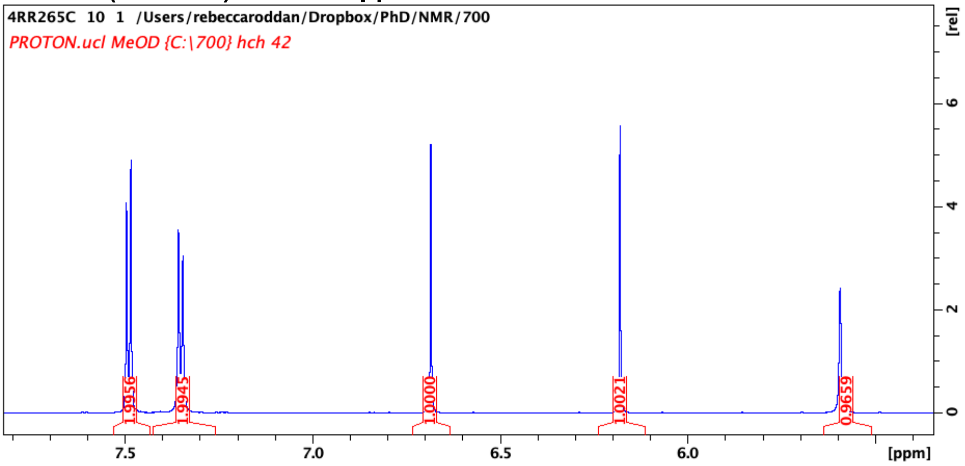

^{1}H-NMR (700 MHz): 3.8 – 2.6 ppm

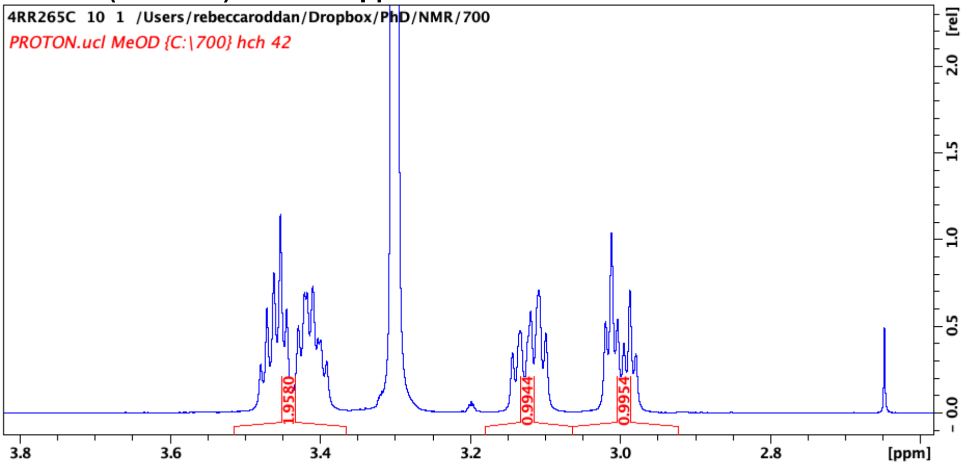

Supplementary Figure 52. ^{1}H-NMR spectra of (1*S*)-1-(4-chlorophenyl)-1,2,3,4-tetrahydroisoquinoline-6,7-diol (**5k**).

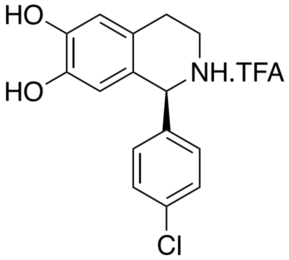

^{13}C-NMR (176 MHz)

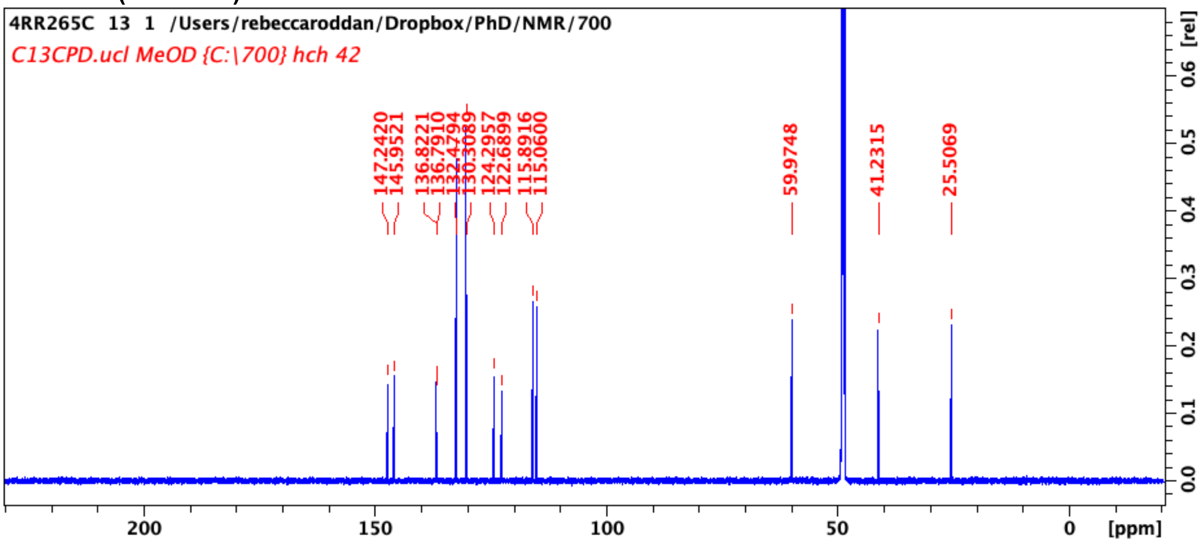

Supplementary Figure 53. ^{13}C-NMR spectrum of (1*S*)-1-(4-chlorophenyl)-1,2,3,4-tetrahydroisoquinoline-6,7-diol (**5k**).

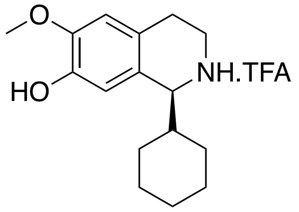

^{1}H-NMR (700 MHz)

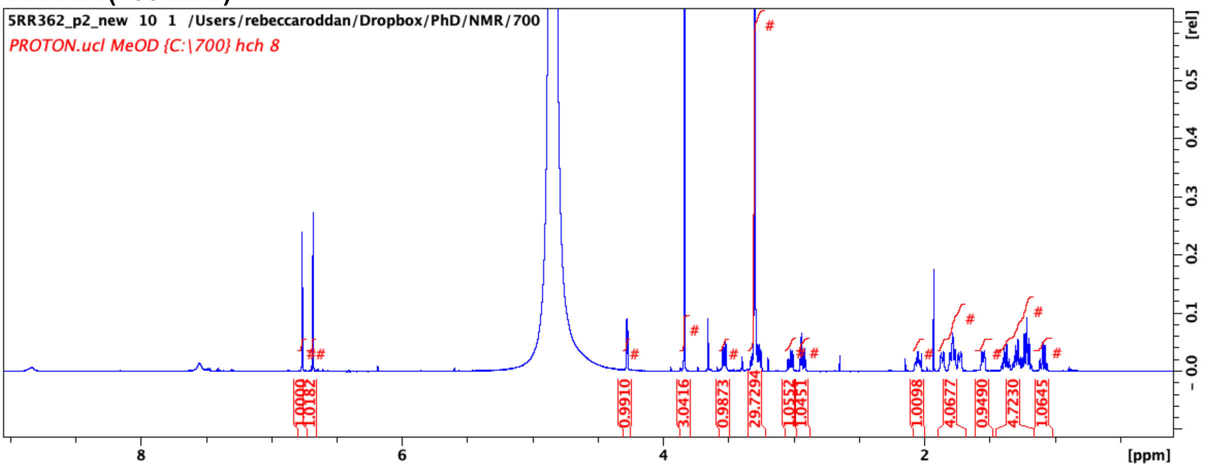

^{1}H-NMR (700 MHz): 4.4 – 2.9 ppm

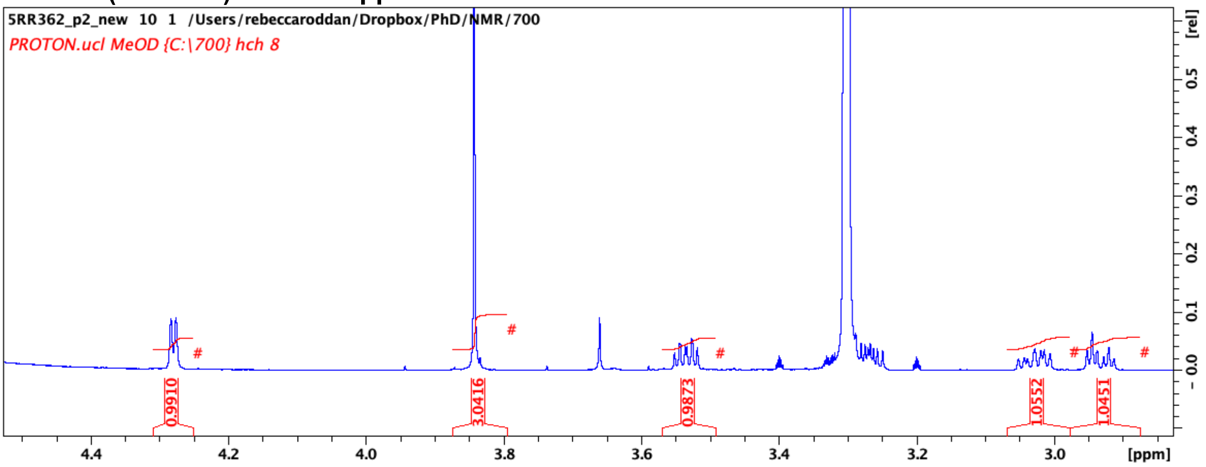

^{1}H-NMR (700 MHz): 2.1 – 1.0 ppm

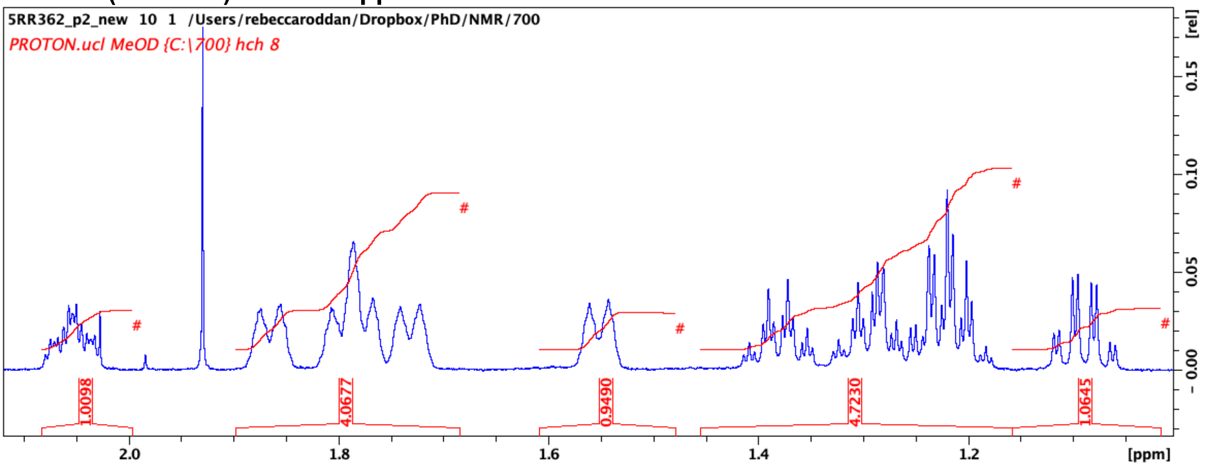

Supplementary Figure 54. ^{1}H-NMR spectra of (1*S*)-1-cyclohexyl-6-methoxy-1,2,3,4-tetrahydroisoquinolin-7-ol (C6-OMe-**7a**).

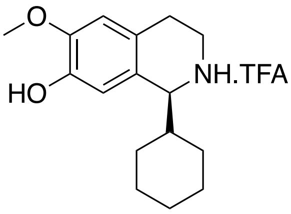

^{13}C-NMR (176 MHz)

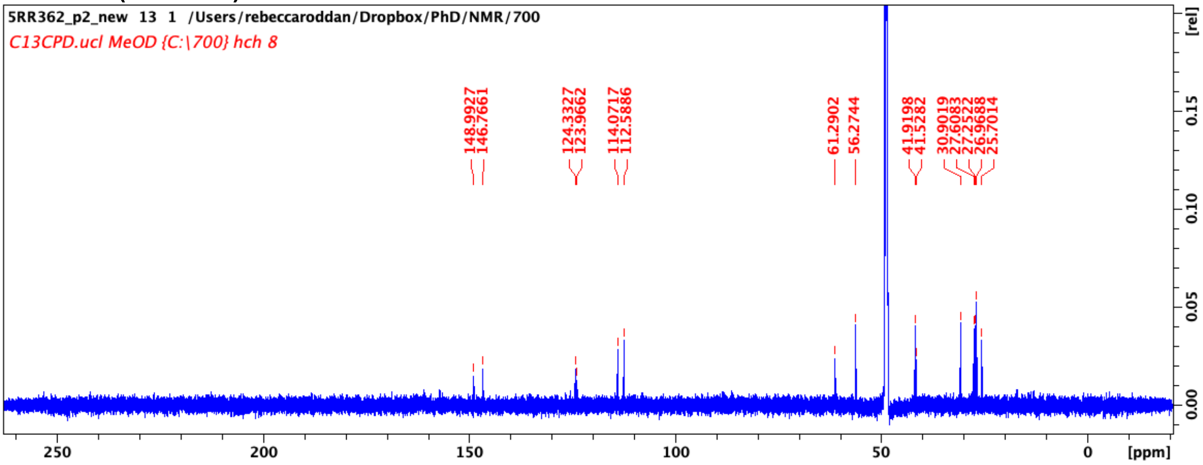

Supplementary Figure 55. ^{13}C-NMR spectrum of (1*S*)-1-cyclohexyl-6-methoxy-1,2,3,4-tetrahydroisoquinolin-7-ol (C6-OMe-**7a**).

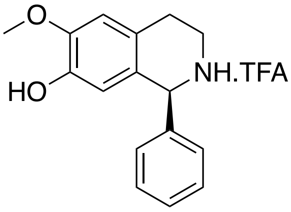

^{1}H-NMR (700 MHz)

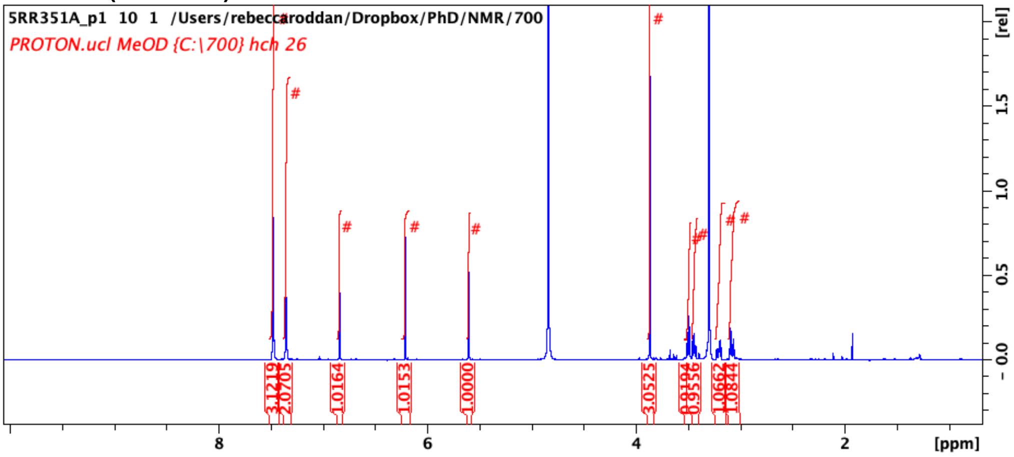

^{1}H-NMR (700 MHz): 7.7 – 5.5 ppm

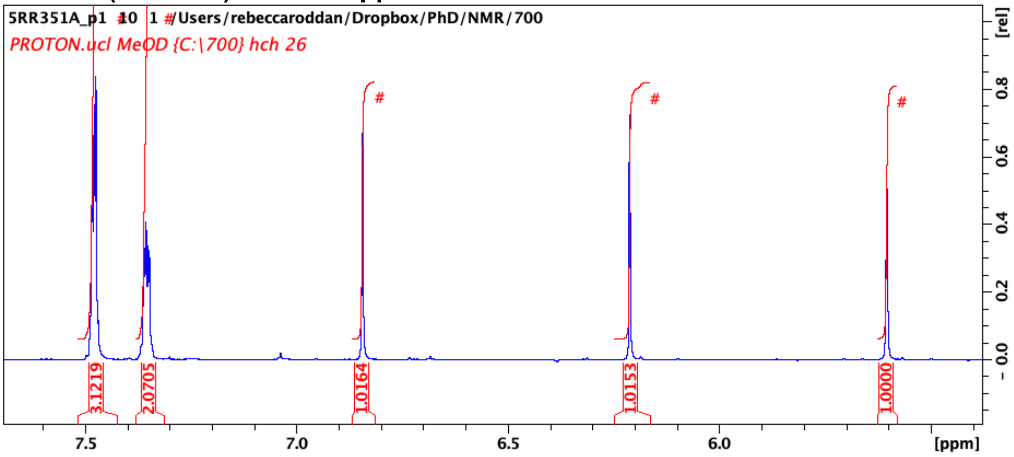

^{1}H-NMR (700 MHz): 3.9 – 3.0 ppm

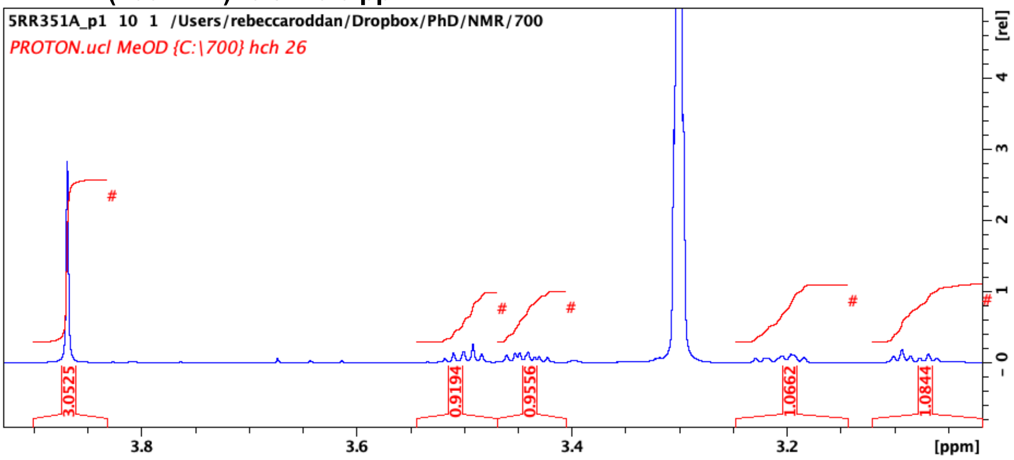

Supplementary Figure 56. ^{1}H-NMR spectra of (1*S*)-6-methoxy-1-phenyl-1,2,3,4-tetrahydroisoquinolin-7-ol (C6-OMe-**7b**).

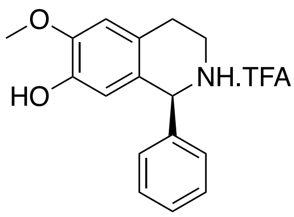

^{13}C-NMR (176 MHz)

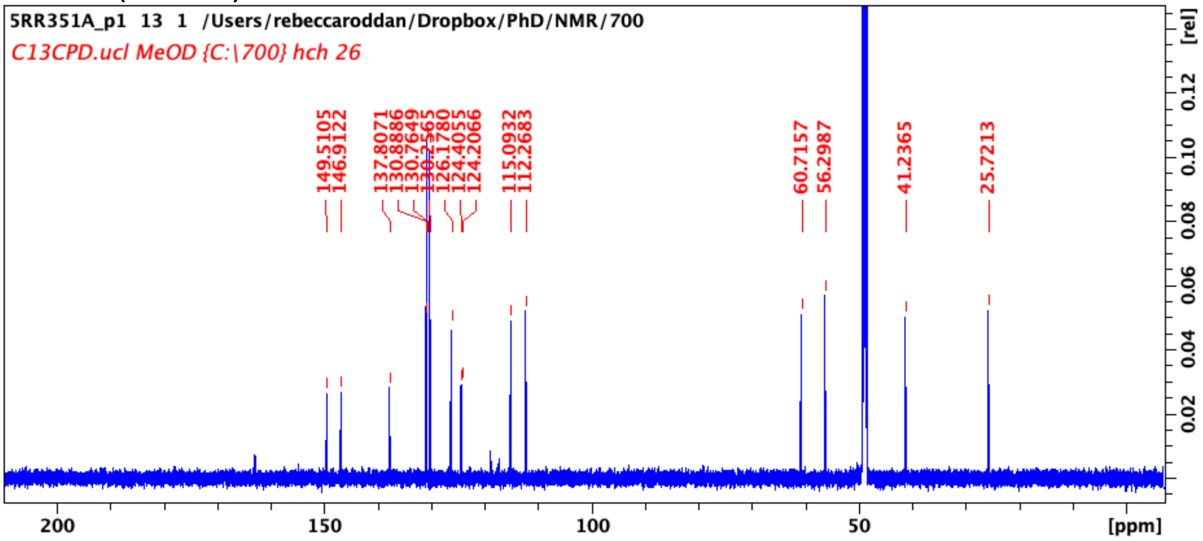

Supplementary Figure 57. ^{13}C-NMR spectrum of (1*S*)-6-methoxy-1-phenyl-1,2,3,4-tetrahydroisoquinolin-7-ol (C6-OMe-**7b**).

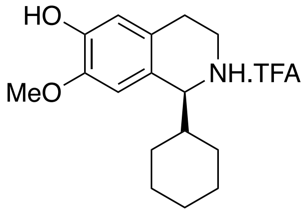

^{1}H-NMR (700 MHz)

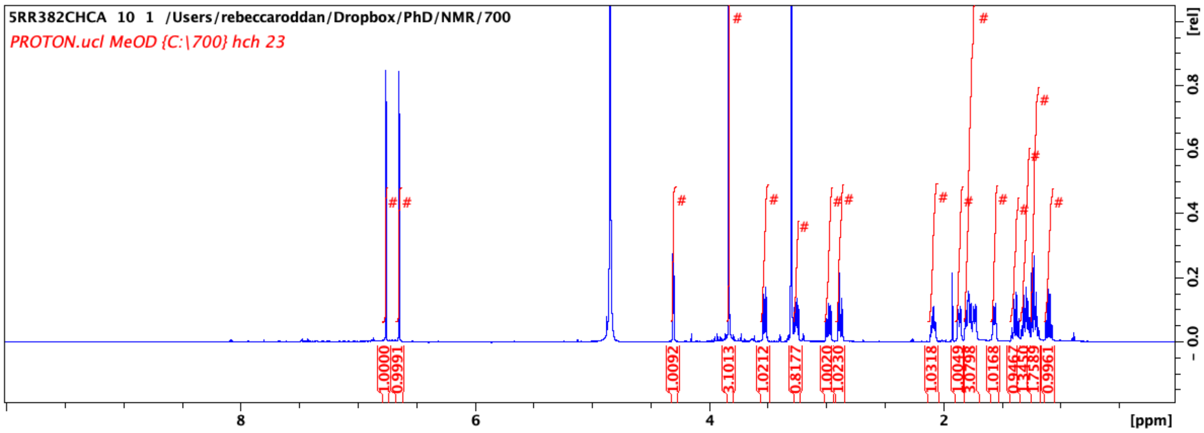

^{1}H-NMR (700 MHz): 4.4 – 2.8 ppm

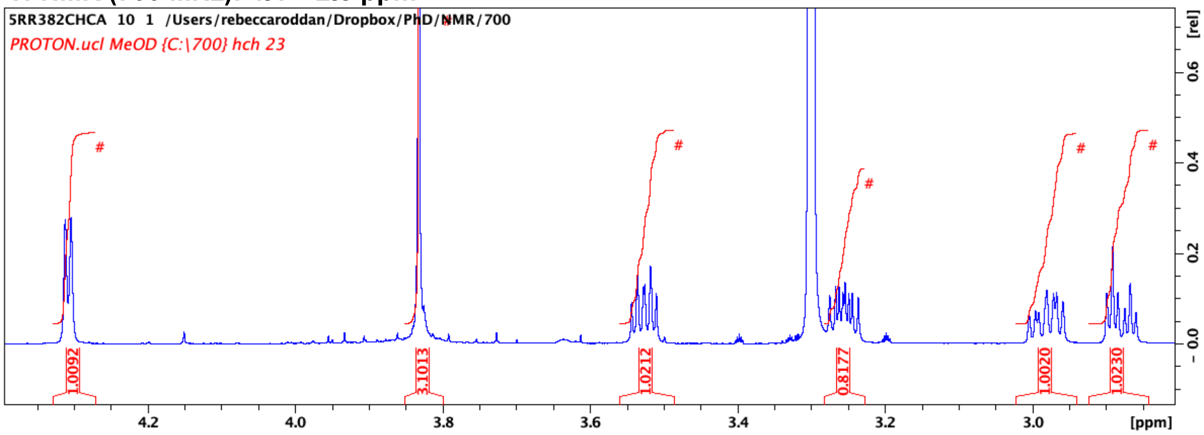

^{1}H-NMR (700 MHz): 2.2 – 1.0 ppm

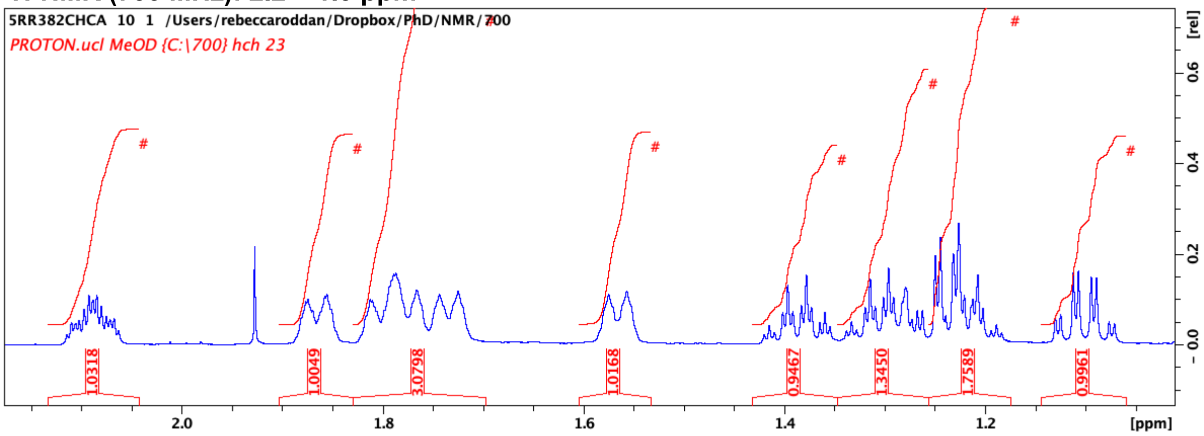

Supplementary Figure 58. ^{1}H-NMR spectra of (1*S*)-1-cyclohexyl-7-methoxy-1,2,3,4-tetrahydroisoquinolin-6-ol (C7-OMe-**7a**).

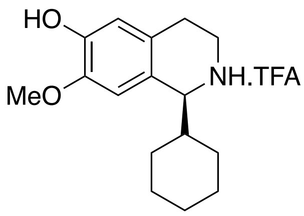

^{13}C-NMR (176 MHz)

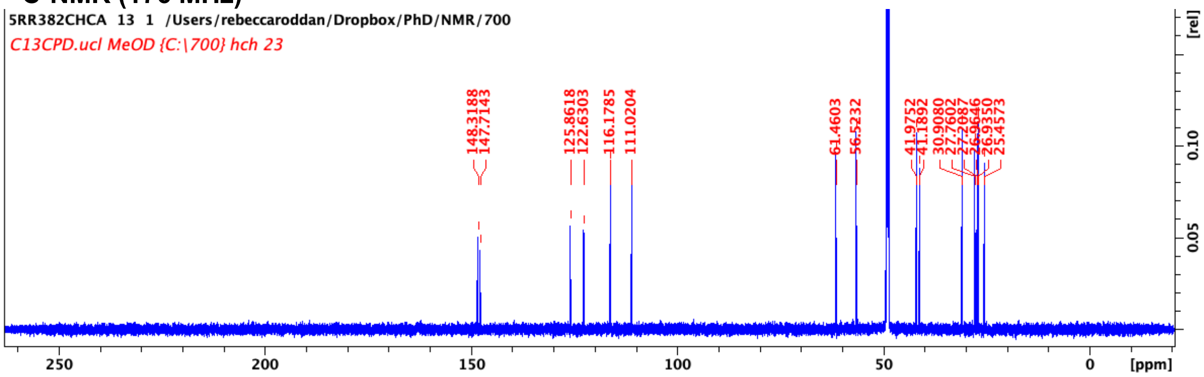

Supplementary Figure 59. ^{13}C-NMR spectrum of (1*S*)-1-cyclohexyl-7-methoxy-1,2,3,4-tetrahydroisoquinolin-6-ol (C7-OMe-**7a**).

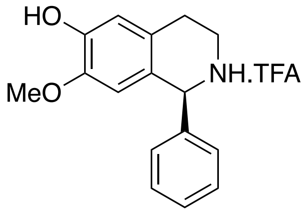

^{1}H-NMR (700 MHz)

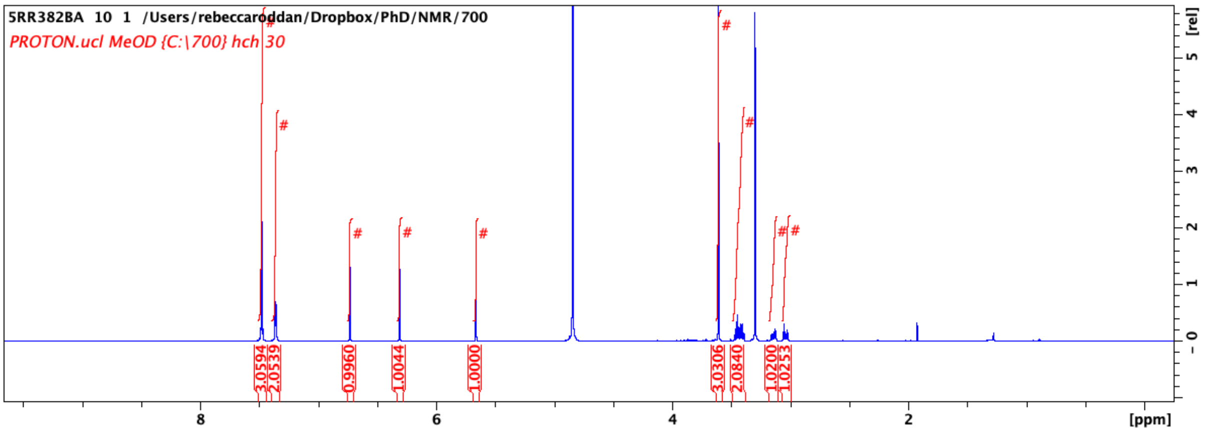

^{1}H-NMR (700 MHz): 7.6 – 5.5 ppm

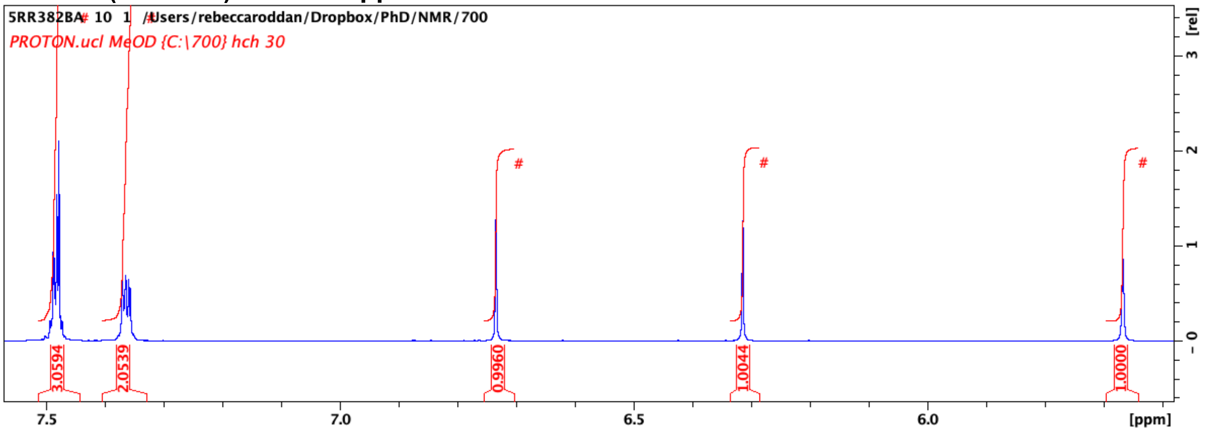

^{1}H-NMR (700 MHz): 3.7 – 2.9 ppm

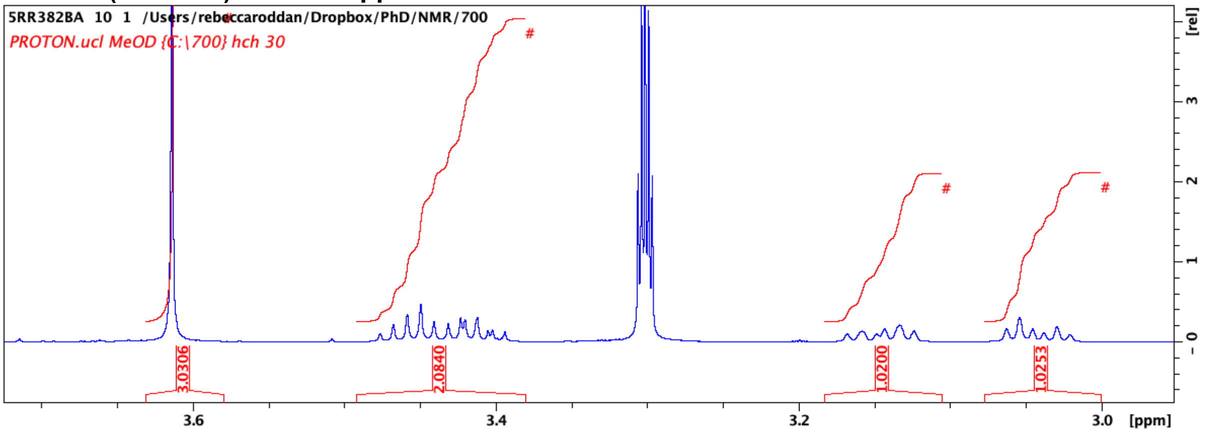

Supplementary Figure 60. ^{1}H-NMR spectra of (1*S*)-7-methoxy-1-phenyl-1,2,3,4-tetrahydroisoquinolin-6-ol (C7-OMe-**7b**).

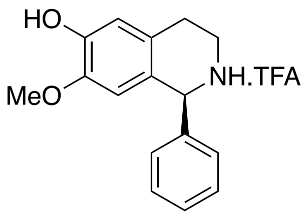

^{13}C-NMR (176 MHz)

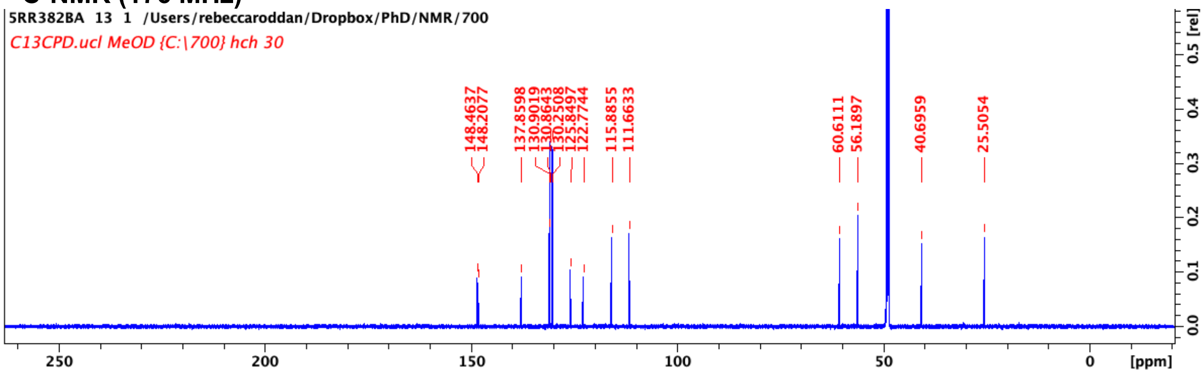

Supplementary Figure 61. ^{13}C-NMR spectrum of (1*S*)-7-methoxy-1-phenyl-1,2,3,4-tetrahydroisoquinolin-6-ol (C7-OMe-**7b**).

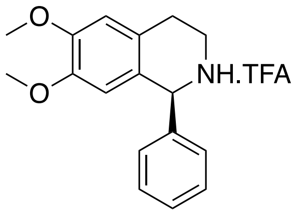

^{1}H-NMR (700 MHz)

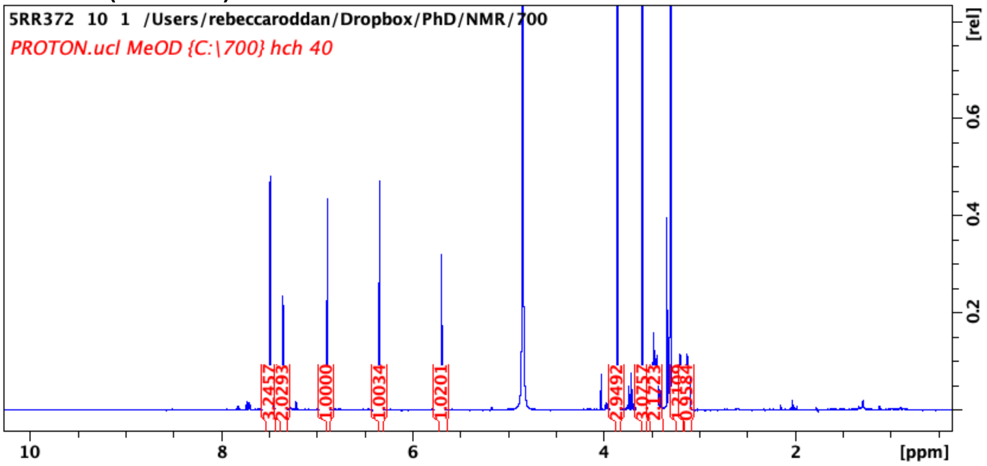

^{1}H-NMR (700 MHz): 7.6 – 5.5 ppm

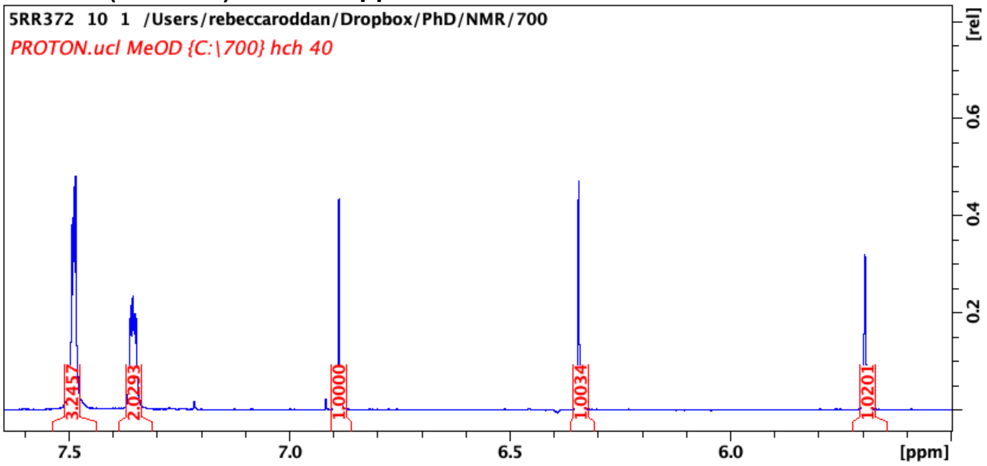

^{1}H-NMR (700 MHz): 4.0 – 3.0 ppm

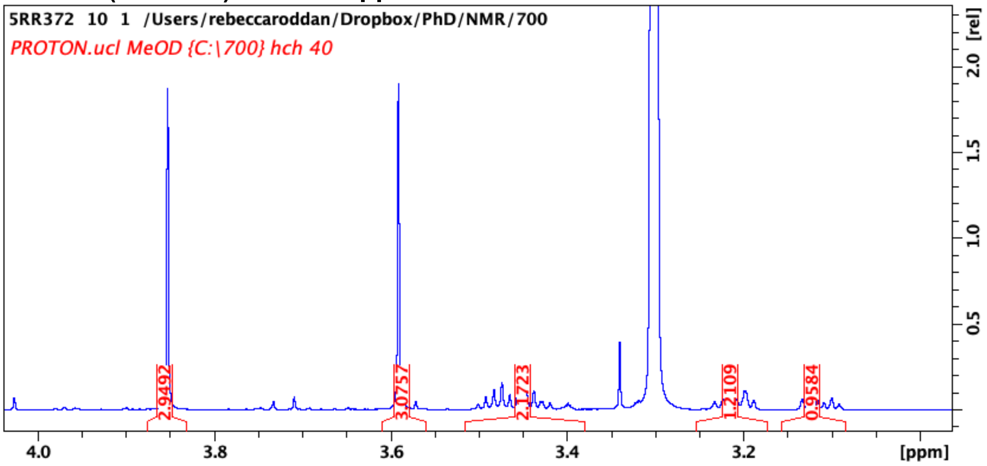

Supplementary Figure 62. ^{1}H-NMR spectra of (1*S*)-6,7-dimethoxy-1-phenyl-1,2,3,4-tetrahydroisoquinoline.

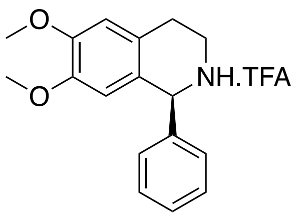

^{13}C-NMR (176 MHz)

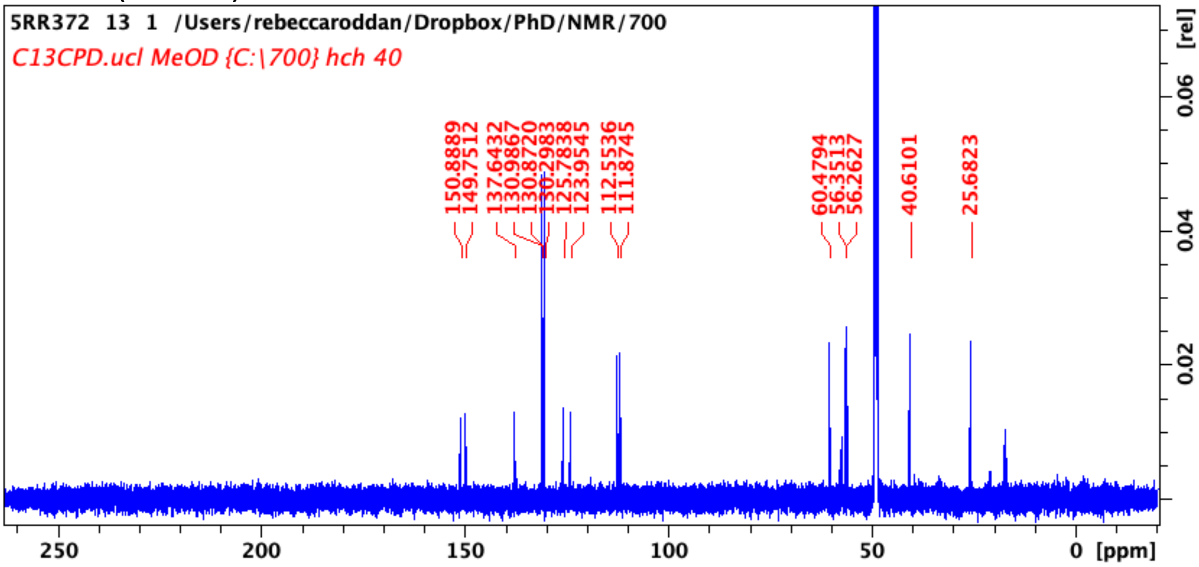

Supplementary Figure 63. ^{13}C-NMR spectrum of (1*S*)-6,7-dimethoxy-1-phenyl-1,2,3,4-tetrahydroisoquinoline.

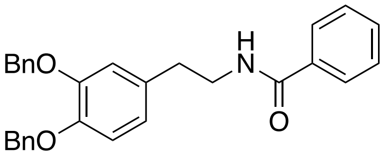

^{1}H-NMR (700 MHz)

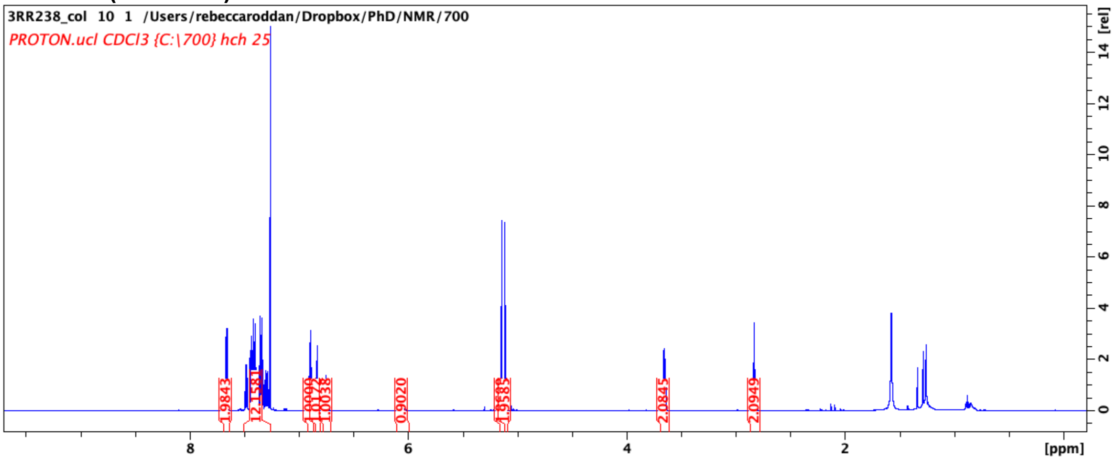

^{1}H-NMR (700 MHz): 7.7 – 5.0 ppm

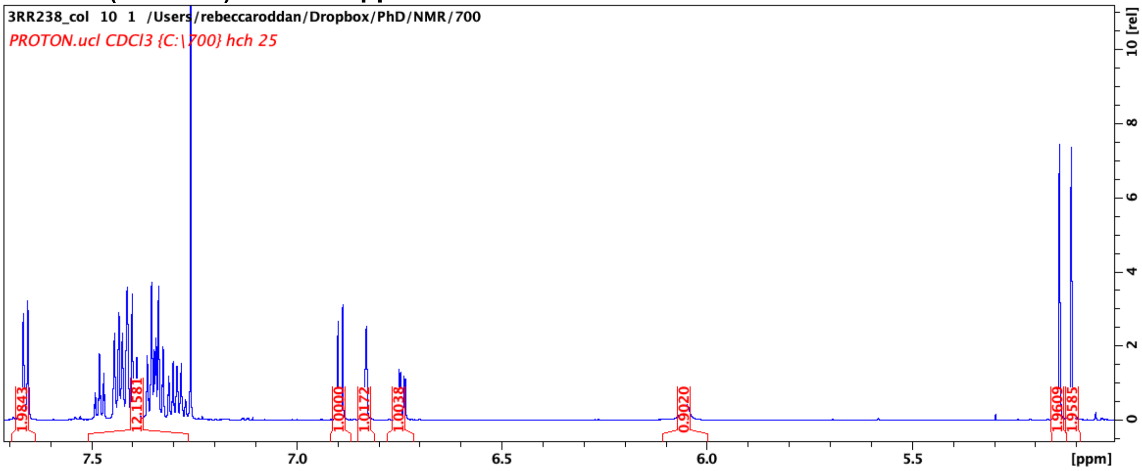

^{1}H-NMR (700 MHz): 3.7 – 2.7 ppm

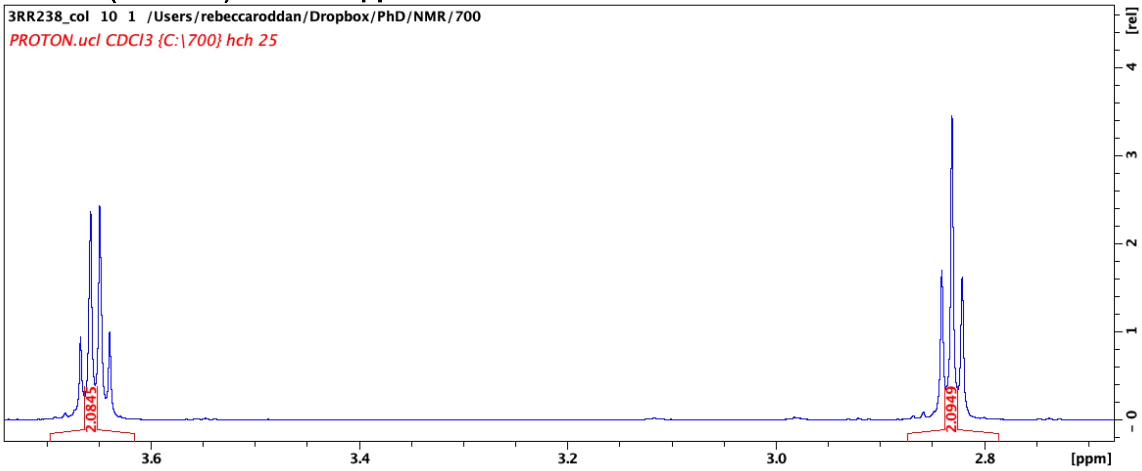

Supplementary Figure 64. ^{1}H-NMR spectra of *N*-(3,4-bis(benzyloxy)phenethyl)benzamide.

^{13}C-NMR (176 MHz)

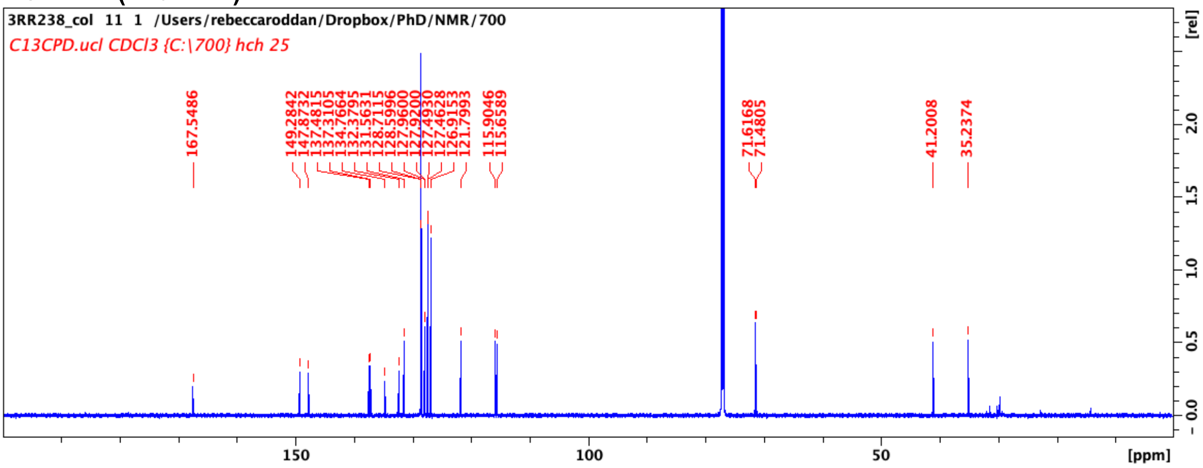

Supplementary Figure 65. ^{13}C-NMR spectrum of *N*-(3,4-bis(benzyloxy)phenethyl)benzamide.

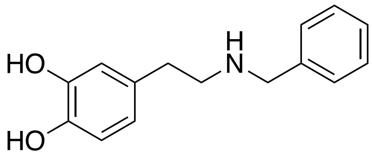

^{1}H-NMR (700 MHz

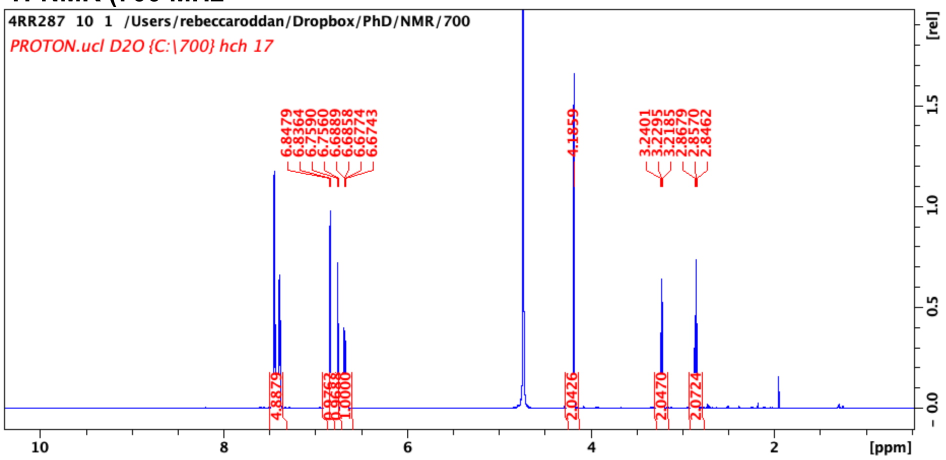

^{1}H-NMR (700 MHz): 7.5 – 6.6 ppm

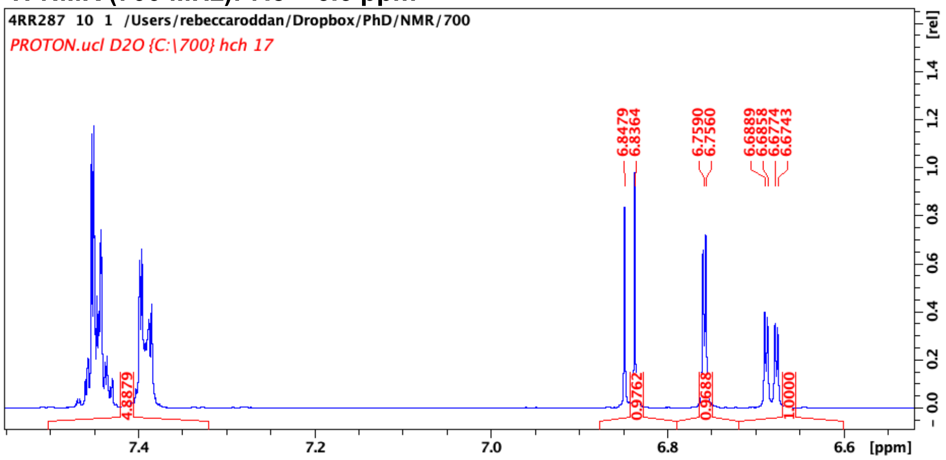

^{1}H-NMR (700 MHz): 4.5 – 2.6 ppm

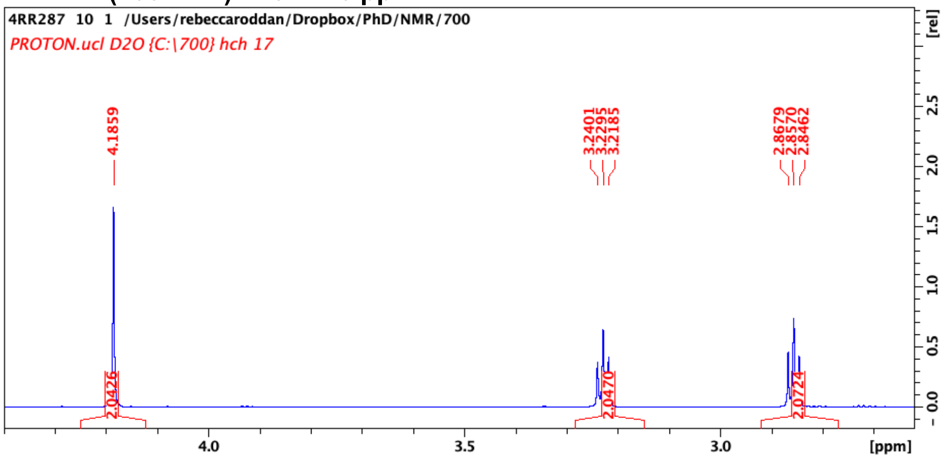

Supplementary Figure 66. ^{1}H-NMR spectra of 4-(2-(benzylamino)ethyl)benzene-1,2-diol (**6**).

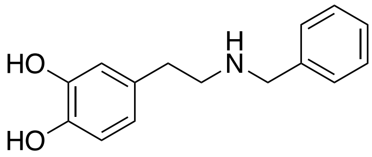

^{13}C-NMR (176 MHz)

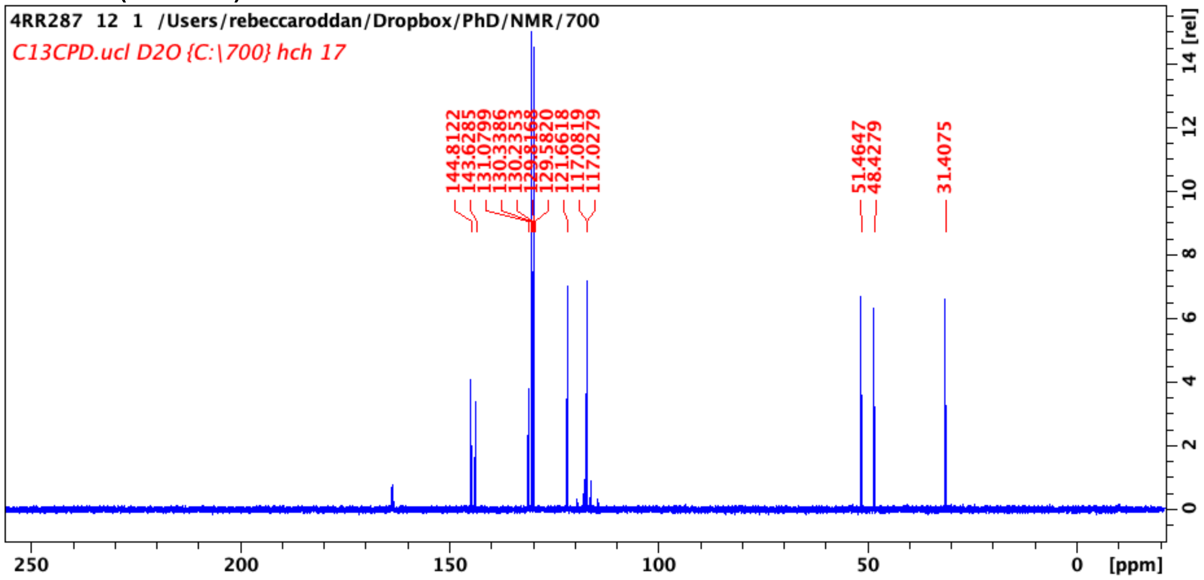

Supplementary Figure 67. ^{13}C-NMR spectrum of 4-(2-(benzylamino)ethyl)benzene-1,2-diol (**6**).

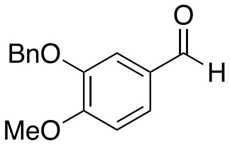

^{1}H-NMR (600 MHz)

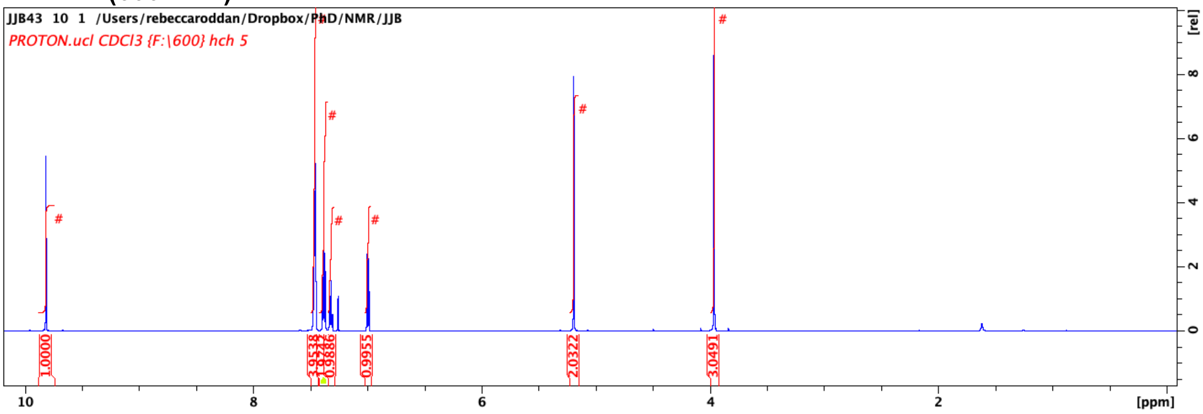

^{1}H-NMR (600 MHz): 7.6 – 6.9 ppm

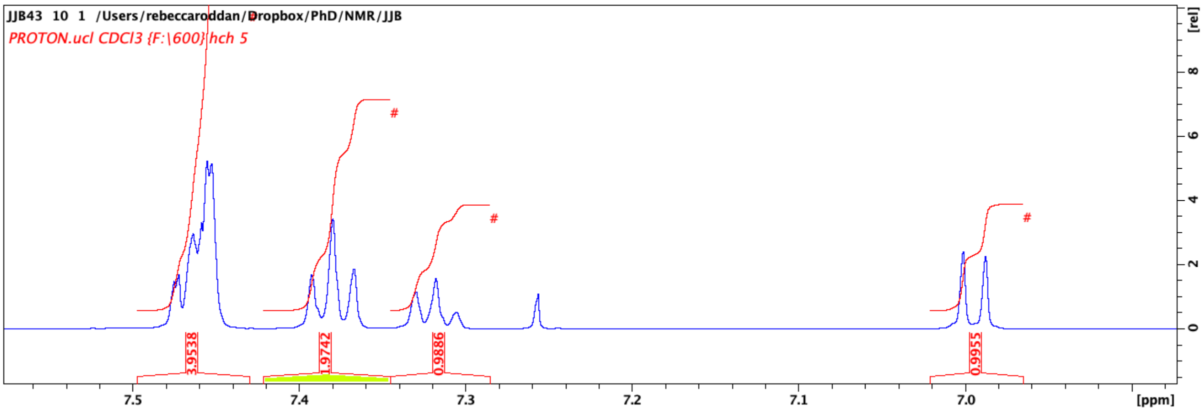

^{1}H-NMR (600 MHz): 5.2 – 3.9 ppm

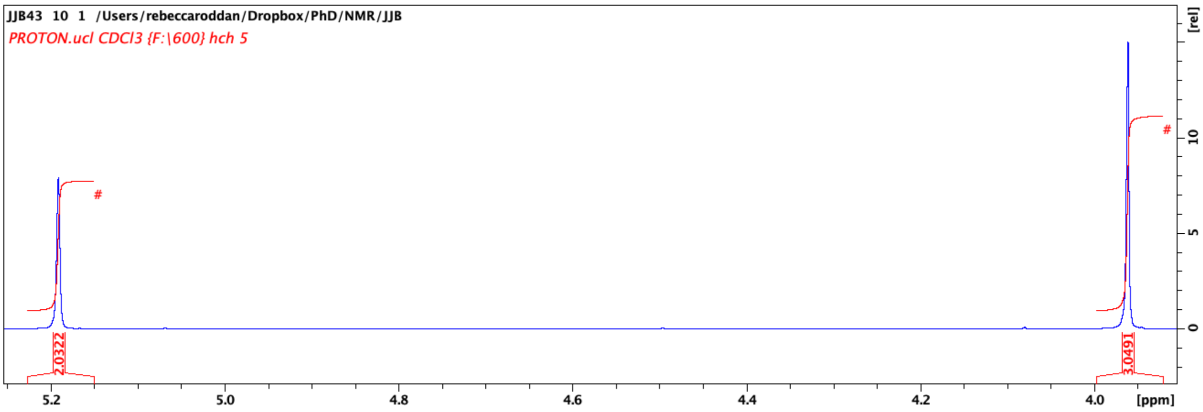

Supplementary Figure 68. ^{1}H-NMR spectra of *O*-benzylvanillin.

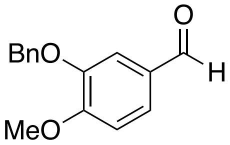

^{13}C-NMR (151 MHz)

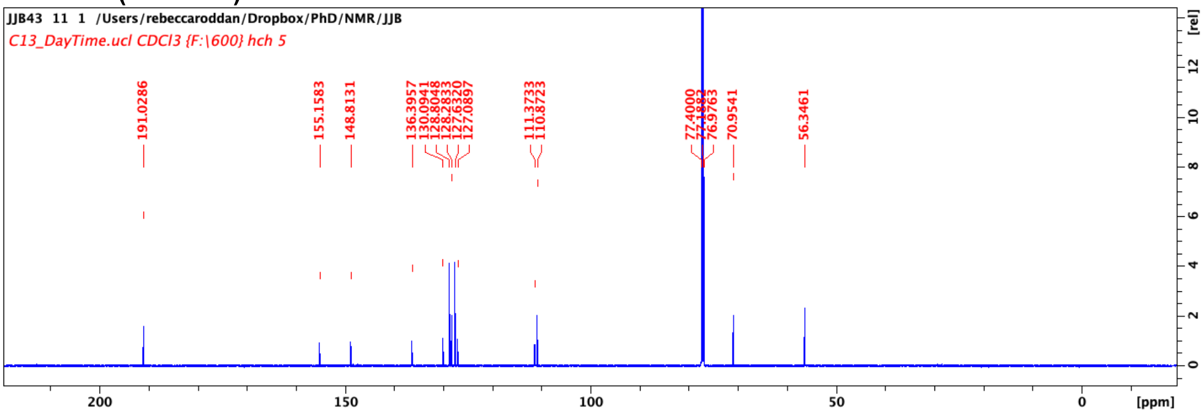

Supplementary Figure 69. ^{13}C-NMR spectrum of *O*-benzylvanillin.

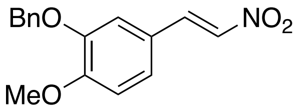

^{1}H-NMR (600 MHz)

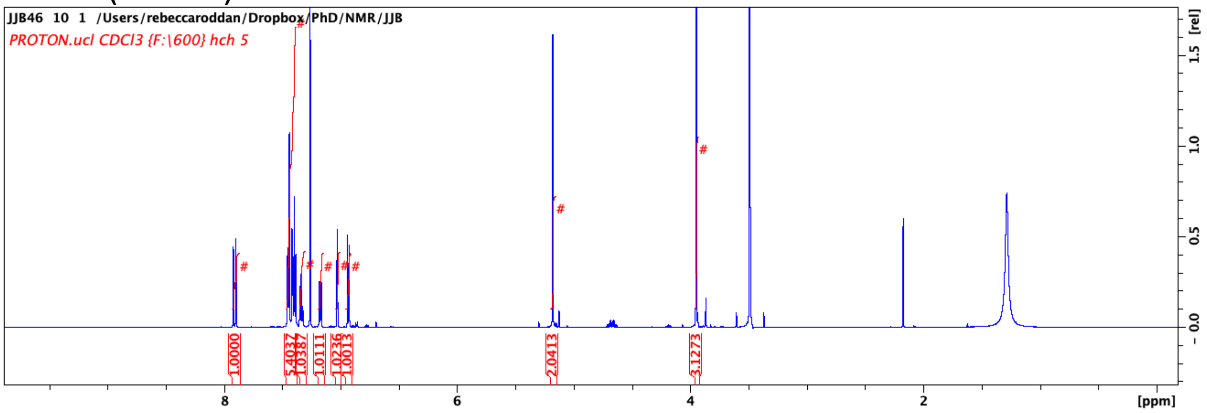

^{1}H-NMR (600 MHz): 8.0 – 6.8 ppm

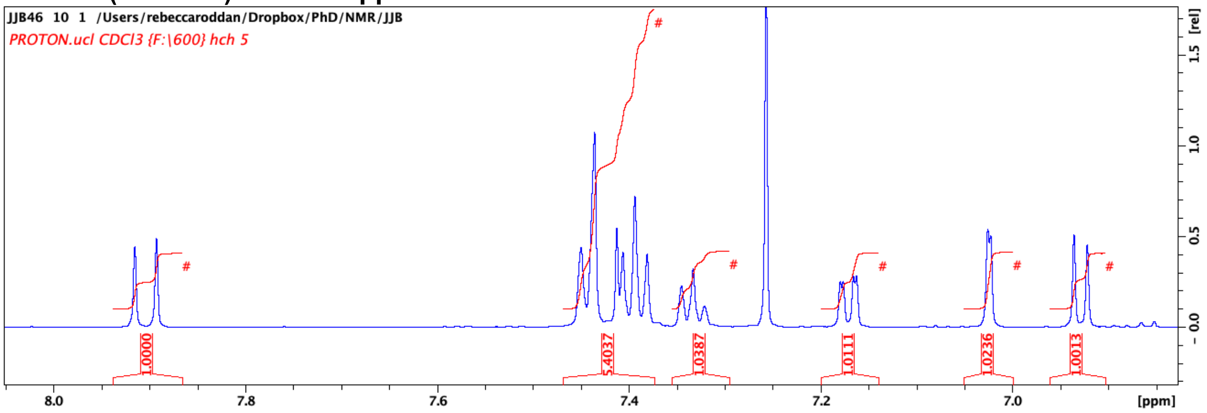

^{13}C-NMR (151 MHz)

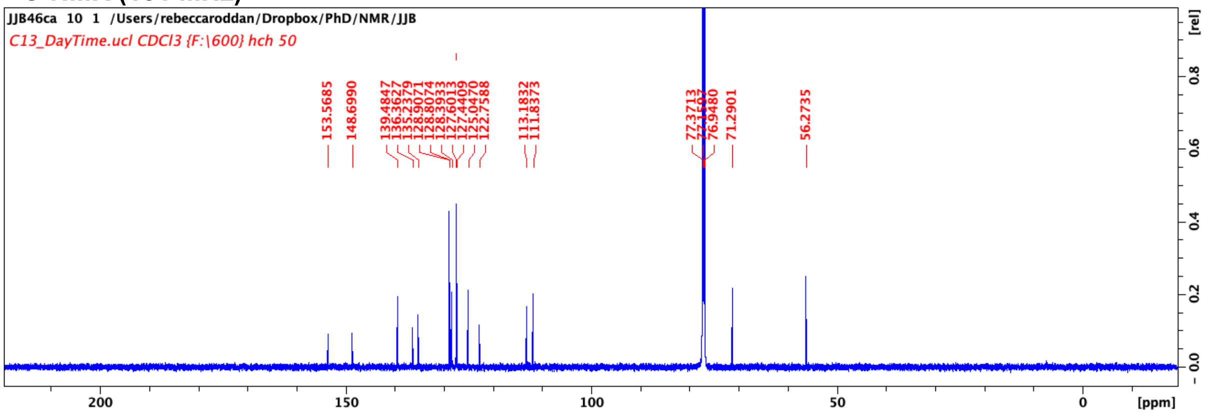

Supplementary Figure 70. NMR spectra of *trans*-3-benzyloxy-4-methoxy-β-nitrostyrene

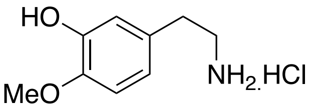

^{1}H-NMR (600 MHz)

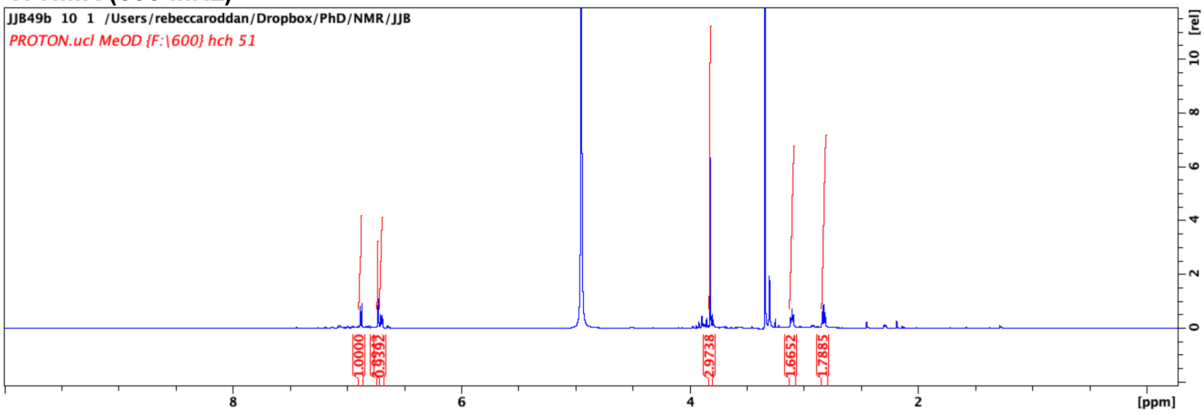

^{1}H-NMR (600 MHz): 7.0 – 6.5 ppm

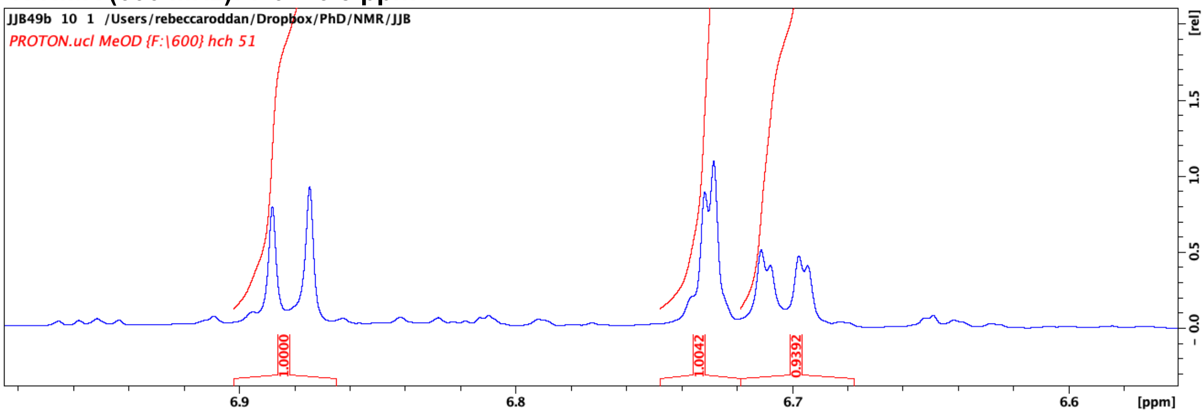

^{1}H-NMR (600 MHz): 4.0 – 2.6 ppm

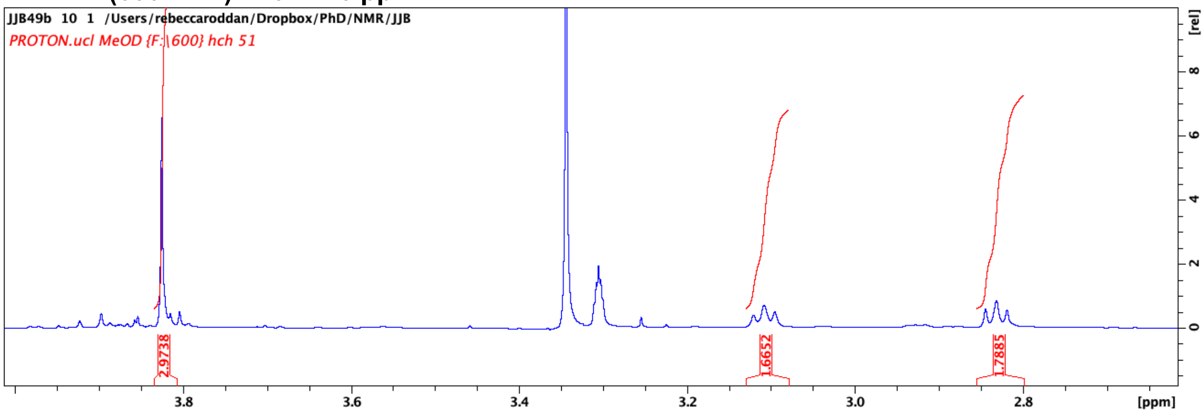

Supplementary Figure 71. ^{1}H-NMR spectra of 4-methoxytyramine hydrochloride.

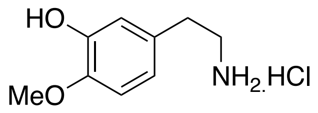

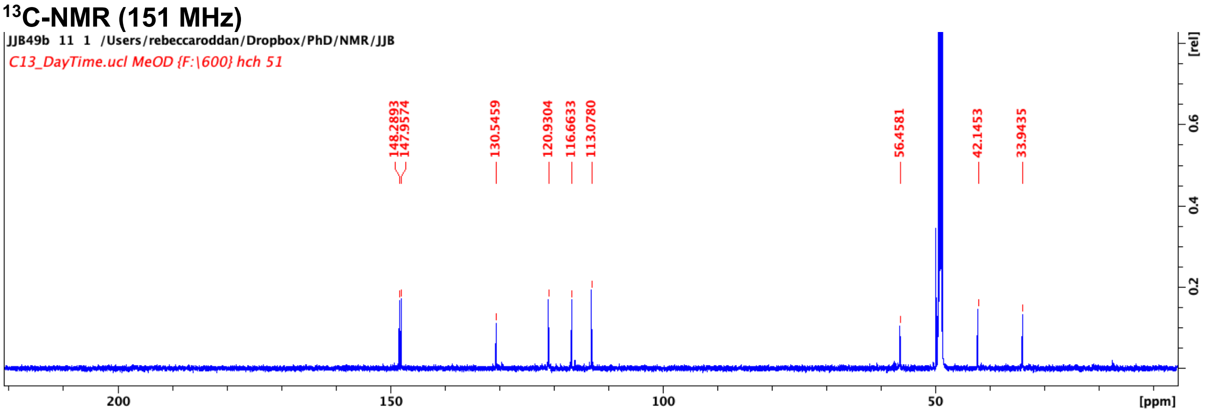

Supplementary Figure 72. ^{13}C-NMR spectrum of 4-methoxytyramine hydrochloride.

Supplementary Tables

Supplementary Table 1. X-ray data collection and refinement statistics for PDB: 6Z82

	**PDB: 6Z82**
**Wavelength (Å)**	0.976254
**Space group**	$P3_221$
**Unit cell parameters**	
**a, b, c (Å)**	62.66, 62.66, 72.65
**α, β, γ (°)**	90.00, 90.00, 120.00
**Resolution range (Å)**	54.27 – 2.30 (2.30-2.38)
**Total number of observations**	78587 (6945)
**Total number unique**	7696 (719)
**Completeness**	99.7 (97.9)
**Multiplicity**	10.2 (9.7)
**$\langle I/\sigma(I) \rangle$**	26.9 (3.8)
**$CC_{1/2}$**	1.000 (0.975)
**R_{merge}**	0.035 (0.353)
**Solvent content (%)**	46
**Molecule per ASU**	1
**Wilson B factor (Å^2)**	69.83
**Refinement**	
**Resolution Range (Å)**	54.27 – 2.30 (2.296 – 2.356)
**R_{work}**	0.197 (0.242)
**R_{free}**	0.246 (0.302)
**Reflection, working**	7285
**Reflection, free**	389
**Average B factor**	79.715
**RMSD bond angle**	1.728
**RMSD bond length (Å)**	0.0128
**Preferred region (%)**	94.87
**Allowed region (%)**	4.49
**Outliers (%)**	0.64

Supplementary Methods

Enzyme sequences

M97V with TEV cleavage site:

MHHHHHHSSGVDLGTE*NLYFQS*MGIINQVSTVTKVIHHELEVAASADDIWTVYSWPGLAKHLPDLLG
AFEKLEIIGDGGVGTILDVTFVPGEFPHEYKEKFILVDNEHRLKKVQMIEGGYLDLGVTYYMDTIHVVPT
GKDSCVIKSSTEYHVKPEFVKIVEPLITTGPLAAMADAISKLVLEHKS

The TEV cleavage site is given in italics.

***Mx*SafC:**

MIHHVELTQSVLQYIRDSSVRDNDILRDLREETSKLPLRTMQIPPEQGQLLSLLVRLIGARKTLEVGVFT
GYSTLCAALALPADGRVIACDLSEEWVSIARRYWQRAGVADRIEVRLGDAHHSLEALVGSEHRGTFD
LAFIDADKESYDFYYEHALRLVRPGGLIILDNTLWSGKVADPSVVGDPETDSLRRINAKLLTDERVDLS
MLPIADGLTLARKRKLAAALEHHHHHH

RnCOMT:

MGSSHHHHHHSSGLVPRGSHMGDTKEQRILRYVQQNAKPGDPQSVLEAIDTYCTQKEWAMNVGDA
KGQIMDAVIREYSPSLVLELGAYCGYSAVRMARLLQPGARLLTMEMNPDYAAITQQMLNFAGLQDKV
TILNGASQDLIPQLKKKYDVDTLDMVFLDHWKDRYLPDTLLLEKCGLLRKGTVLLADNVIVPGTPDFLA
YVRGSSSFECTHYSSYLEYMKVVDGLEKAIYQGPSSPDKS

***Ec*MAT:**

MGSSHHHHHHSSGLVPRGSHMAKHLFTSESVSEGHPDKIADQISDAVLDAILEQDPKARVACETYVK
TGMVLVGGEITTSAWVDIEEITRNTVREIGYVHSDMGFDANSCAVLSAIGKQSPDINQGVDRADPLEQ
GAGDQGLMFGYATNETDVLMPAPITYAHRLVQRQAEVRKNGTLPWLRPDAKSQVTFQYDDGKIVGI
DAVVLSTQHSEEIDQKSLQEAVMEEIIKPILPAEWLTSATKFFINPTGRFVIGGPMGDCGLTGRKIIVDT
YGGMARHGGGAFSGKDPSKVDRSAAYAARYVAKNIVAAGLADRCEIQVSYAIGVAEPTSIMVETFGT
EKVPSEQLTLLVREFFDLRPYGLIQMLDLLHPIYKETAAYGHFGREHFPWEKTDKAQLLRDAAGLK

***Ec*MTAN:**

MGSSHHHHHHSSGLVPRGSHMKIGIIGAMEEEVTLLRDKIENRQTISLGGCEIYTGQLNGTEVALLKS
GIGKVAAALGATLLLEHCKPDVIINTGSAGGLAPTLKVGDIVVSDEARYHDADVTAFGYEYGQLPGCP
AGFKADDKLIAAAEACIAELNLNAVRGLIVSGDAFINGSVGLAKIRHNFPQAIAVEMEATAIAHVCHNFN
VPFVVVRAISDVADQQSHLSFDEFLAVAAKQSSLMVESLVQKLAHG

Sequences, expression and purification of M97F, M97V,L76V-Δ29*Tf*NCS have been reported previously.[3]

Plasmid details

For the ∆29*Tf*NCS wild-type and mutants, the vector used was pJ411, obtained from DNA 2.0. The constructs contain a C-terminal hexahistidine tag and were prepared via site-directed mutagenesis, performed using a QuikChange® Lightening mutagenesis kit, as previously reported.[4] Primers were purchased from Eurofins and sequences were confirmed by Sanger sequencing by Source BioSciences. A glycerol stock of *E. coli* BL21 (DE3) containing the expression vector for TEV Protease (pRK793) was obtained from Carolyn Moores (Department of Biological Sciences, Birkbeck College, London).

Plasmid details for *Ec*MAT, *Ec*MTAN, *Mx*SafC and *Rn*COMT have been previously reported.[5,6]

Protein crystallization and data processing

The positive difference density observed in the active site of the enzyme of the unrefined structure was significantly larger than what would be expected for a water molecule. Subsequent rounds of structure refinement led to a larger volume of difference density into which the ligand was built. It was only possible to model the ligand in one orientation to be consistent with the 'dopamine-first' mechanism. The NCS mechanism is known to be highly dynamic, so it is likely that other minor, pseudo-conformations of the ligand are also present.

Phosphate-mediated reactions

A solution of the amine (10 mM), sodium ascorbate (10 mM) and aldehyde (20 mM) were prepared in 1:1 MeCN:KPi buffer (300 mM, pH 6). Reactions were stirred at 60 °C for 18 h and HPLC samples prepared using workup methods 1 or 2.

To generate racemic standards of the 1-phenyl THIQs for chiral HPLC analysis, the phosphate-mediated Pictet-Spengler reaction between dopamine and a variety of aldehyde derivatives (**4a-k**, Supplementary Figure 7) was investigated.[7] Reactions were performed using previously reported conditions.[7] Conversions were in the range (14 - 86%), as shown in Figure S8. Significant precipitation was observed in the reaction samples and additional peaks were also observed during RP-HPLC analysis that did not correspond to the THIQ products isolated from enzymatic reactions. This suggests that side reactions (e.g. oxidations) occur due to the highly reactivity of the benzaldehydes. To minimise this, reactions were performed under an argon atmosphere and it is likely that some of the side-products adhered to the plastic Eppendorf tubes that the reactions were performed in.

For reactions between dopamine and linear or benzylic aldehydes, a ratio of the *ortho*:*para* isomers of product of 1:3 has been observed, so single regioisomer products were typically isolated by preparative-HPLC. For reactions between dopamine and α-methyl substituted aldehydes, only the *para* regioisomer was generated, likely due to steric hinderance. The conversions and regioisomer ratios of phosphate mediated reactions between dopamine and a variety of benzaldehyde analogues are given in Supplementary Figure 7. For the majority of substrates, a ratio of the two regioisomers, *ortho:para* of 8-10:1 was observed.

Chiral HPLC analyses

Chiral HPLC analysis (method indicated for each compound) was performed for the 1-aryl-THIQs generated by the NCS-mediated synthesis to determine the enantiomeric excess. Racemic standards (denoted by 'KPi') were prepared using a biomimetic phosphate mediated Pictet-Spengler reaction.[7] Retention times (t_R) varied slightly depending on minor discrepancies in mobile phases and temperatures.

Reaction conditions for enzymatic reactions: 10 mM dopamine·HCl, 20 mM aldehyde, 10 mM sodium ascorbate, 0.2 mg mL^{-1} purified Δ29*Tf*NCS (wild-type = WT, M97F-Δ29*Tf*NCS = M97F, M97V-Δ29*Tf*NCS = M97V, L76V-Δ29*Tf*NCS = L76V) in HEPES buffer (100 mM, pH 7.5) with 10% v/v MeCN at 37 ^{o}C for 3 h, 100 µL scale reactions.

Reaction conditions used for generation of the racemic product: 10 mM dopamine·HCl, 20 mM aldehyde, 10 mM sodium ascorbate in phosphate buffer (0.3 M, pH 6) with 50% *v/v* MeCN at 60 ^{o}C, for 18h. Reactions were performed on a 500 µL scale.

Sample preparation for chiral HPLC injection: If chiral HPLC method 1 was used, samples were prepared using workup method 1 (Methods). Samples were prepared using workup method 2 (Methods), if chiral HPLC method 2 was used. An injection volume of 10 µL was used for all samples.

Enzymatic scale-ups and characterisation of resultant products

General method for enzymatic reactions: A solution of amine (final reaction concentration of 10 mM) and sodium ascorbate (final reaction concentration of 10 mM) was prepared in HEPES buffer (50 mM, pH 7.5 (except Figure 1c where pH 6 was used)). A solution of aldehyde (200 mM in MeCN) was prepared and the two solutions mixed in a 9:1 ratio. *Tf*NCS (at approximately 10 mg mL^{-1} in 20 mM Tris, 50 mM NaCl, pH 7.5) was added and the reactions stirred at 37 °C. Control (no enzyme) reactions were performed using the same conditions but the *Tf*NCS sample was substituted for enzyme buffer (20 mM Tris, 50 mM NaCl, pH 7.5).

Enzymatic reactions towards products for isolation and characterisation purposes were performed on a 10 mL scale using a 0.2 mg mL^{-1} final concentration of Δ29*Tf*NCS-M97V (unless specified) for 3 h at 37 °C. Once the reaction was complete, workup method 3 was performed and the sample purified by preparative-HPLC (method indicated for each product). Methyltransferase reactions to give **7a** and **7b** were performed as described in the methods section and also prepared by workup method 3 then purified by preparative-HPLC (HPLC method indicated for each product). Isolated yields are given after purification by preparative-HPLC.

(1*S*)-1-Cyclohexyl-1,2,3,4-tetrahydroisoquinoline-6,7-diol (5a)

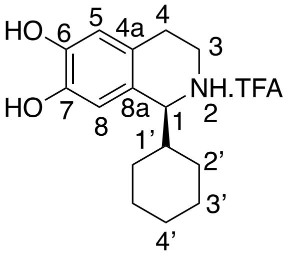

Purified by preparative HPLC method 1 and isolated as a white solid (18 mg, 52%). mp > 250 °C (H_2O); $[\alpha]_D^{25}$ 8.2 (*c* 0.38, MeOH); ν_{max}/ cm^{-1} (thin film): 3354, 2933, 2835, 1622, 1530; ^{1}H-NMR (700 MHz; D_2O) δ 6.73 (1H, s, 5-H or 8-H), 6.71 (1H, s, 5-H or 8-H), 4.26 (1H, d, *J* = 5.0 Hz, 1-H), 3.54 – 3.48 (1H, dt, *J* = 12.3, 5.3 Hz, 3-*H*H), 3.25 – 3.19 (1H, m, 3-H*H*), 2.97 – 2.90 (1H, m, 4-*H*H), 2.84 (1H, dt, *J* = 16.8, 5.3 Hz, 4-H*H*), 2.01 (1H, m, 1'-H), 1.76 (1H, m), 1.68 (2H, m), 1.63 (1H, m), 1.45 (1H, m), 1.29 – 1.05 (4H, m), 0.97 (1H, qd, *J* = 12.6, 3.6 Hz); ^{13}C-NMR (176 MHz; D_2O) δ 146.6, 145.7, 124.3, 122.6, 116.1, 114.2, 61.4, 41.9, 41.5, 30.9, 27.7, 27.3, 27.0, 27.0, 25.5; *m/z* [ES+] 248 ($[M+H]^+$, 100%); *m/z* [HRMS ES+] found $[M+H]^+$ 248.1645; $C_{15}H_{21}NO_2$ requires 248.1645; Retention time (achiral analytical HPLC method 1): 5.4 min.

(1*S*)-1-Phenyl-1,2,3,4-tetrahydroisoquinoline-6,7-diol (5b)

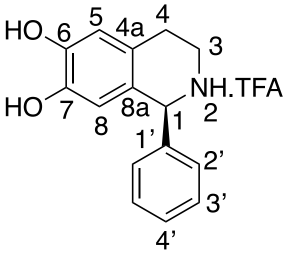

Purified by preparative HPLC method 1 and isolated as a white solid (17 mg, 51%). mp > 250 °C (H_2O); $[\alpha]_D^{25}$ 6.5 (*c* 0.37, MeOH); ν_{max}/ cm^{-1} (thin film): 3033, 2852, 1668, 1612, 1527; ^{1}H-NMR (700 MHz; D_2O) δ 7.49 – 7.43 (3H, m, 3'-H and 4'-H), 7.34 – 7.29 (2H, m, 2'-H), 6.81 (1H, s, 8-H), 6.34 (1H, s, 5-H), 5.63 (1H, s, 1-H), 3.50 – 3.39 (2H, m, 3-H), 3.07 (2H, dt, *J* = 17.2, 5.5 Hz, 4-H); ^{13}C-NMR (176 MHz; D_2O) δ 147.1, 145.9, 138.0, 130.8, 130.8, 130.2, 124.2, 123.1, 115.8, 115.2, 60.9, 41.4, 25.5; *m/z* [MS ES+] 242 ([M+H]$^+$, 100%); *m/z* [HRMS ES+] found [M+H]$^+$ 242.1176; $C_{15}H_{15}NO_2$ requires 242.1176; Retention time (achiral analytical HPLC method 1): 4.9 min.

(1*S*)-1-(*o*-Tolyl)-1,2,3,4-tetrahydroisoquinoline-6,7-diol (5c)

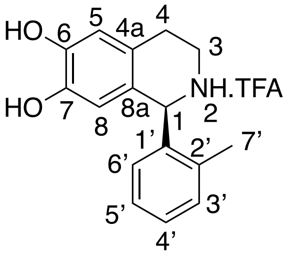

Purified by preparative HPLC method 1 and isolated as a white solid (8.7 mg, 25%); $[\alpha]_D^{25}$ 8.8 (*c* 0.43, MeOH); ν_{max}/ cm^{-1} (thin film): 3024, 2818, 1663, 1609, 1526; ^{1}H-NMR (700 MHz; CD_3OD) δ 7.38 – 7.30 (2H, m, 4'-H and 5'-H), 7.29 – 7.24 (1H, m, 3'-H), 7.10 (1H, d, *J* = 7.8 Hz, 2'-H), 6.68 (1H, s, 5-H), 6.08 (1H, s, 8-H), 5.80 (1H, s, 1'-H), 3.56 – 3.47 (2H, m, 3-H), 3.21 – 3.15 (1H, m, 4-*H*H), 3.04 – 2.97 (1H, dt, *J* = 17.2, 5.3 Hz, 4-*H*H), 2.51 (3H, s, 7'-H); ^{13}C-NMR (176 MHz; CD_3OD) δ 147.0, 145.9, 138.8, 136.3, 132.2, 130.7, 130.3, 128.0, 124.2, 123.8, 115.9, 114.9, 57.2, 42.0, 25.6, 19.3; *m/z* [ES+] 256 ([M+H]$^+$, 100%); *m/z* [HRMS ES+] found [M+H]$^+$ 256.1331; $C_{16}H_{18}NO_2$ requires 256.1332; Retention time (achiral analytical HPLC method 1): 5.2 min.

(1*S*)-1-(*m*-Tolyl)-1,2,3,4-tetrahydroisoquinoline-6,7-diol (5d)

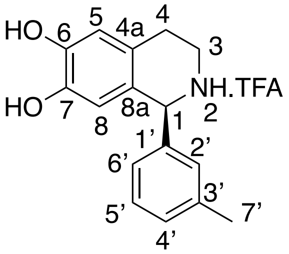

Purified by preparative HPLC method 1 and isolated as a white solid (4.3 mg, 12%). $[\alpha]_D^{25}$ 6.1 (*c* 0.18, MeOH); ν_{max}/ cm^{-1} (thin film): 3436, 2977, 2809, 1665, 1607, 1527; ^{1}H-NMR (700 MHz; CD_3OD) δ 7.36 (1H, t, *J* = 7.7 Hz, 5'-H), 7.29 (1H, d, *J* = 7.7 Hz, 4'-H), 7.18 (1H, s, 2'-H), 7.15 (1H, d, *J* = 7.7 Hz, 6'-H), 6.68 (1H, s, 5-H), 6.19 (1H, s, 8-H), 5.51 (1H, s, 1-H), 3.50 – 3.45 (1H, m, 3-H), 3.43 – 3.38 (1H, m, 3-H), 3.17 – 3.11 (1H, m, 4-*H*H), 3.00 (1H, dt, *J* = 17.2, 5.3 Hz, 4-*H*H), 2.37 (3H, s, 7'-H); ^{13}C-NMR (176 MHz; MeOD) δ 147.1, 145.8, 140.3, 138.0, 131.5, 131.2, 130.1, 127.8, 124.2, 123.2, 115.8, 115.2, 60.9, 41.4, 25.6, 21.2; *m/z* [ES+] 256 ($[M+H]^+$, 100%); *m/z* [HRMS ES+] found $[M+H]^+$ 256.1332; $C_{16}H_{18}NO_2$ requires 256.1332; Retention time (achiral analytical HPLC method 1): 5.3 min.

(1*S*)-1-(*p*-Tolyl)-1,2,3,4-tetrahydroisoquinoline-6,7-diol (5e)

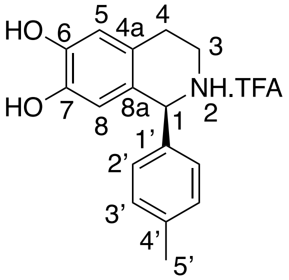

Purified by preparative HPLC method 1 and isolated as a white solid (3.8 mg, 11%). $[\alpha]_D^{25}$ 1.3 (*c* 0.091, MeOH); ^{1}H-NMR (700 MHz; CD_3OD) δ 7.29 (2H, d, *J* = 8.0 Hz, 3'-H), 7.23 (2H, d, *J* = 8.0 Hz, 2'-H), 6.67 (1H, s, 5-H), 6.19 (1H, s, 8-H), 5.51 (1H, s, 1'-H), 3.48 – 3.43 (1H, m, 3-H), 3.42 – 3.37 (1H, m, 3-H), 3.16 – 3.09 (1H, m, 4-*H*H), 3.99 (1H, dt, *J* = 17.2, 5.5 Hz, 4-*H*H), 2.37 (3H, s, 5'-H); ^{13}C-NMR (176 MHz; CD_3OD) δ 147.0, 145.8, 141.1, 135.1, 130.8, 130.6, 124.2, 123.4, 115.8, 115.2, 60.7, 41.3, 25.6, 21.1; *m/z* [MS ES+] 256 ($[M+H]^+$, 100%); *m/z* [HRMS ES+] found $[M+H]^+$ 256.1331; $C_{16}H_{18}NO_2$ requires 256.1332; Retention time (achiral analytical HPLC method 1): 5.3 min.

(1*S*)-1-(2-Fluorophenyl)-1,2,3,4-tetrahydroisoquinoline-6,7-diol (5f)

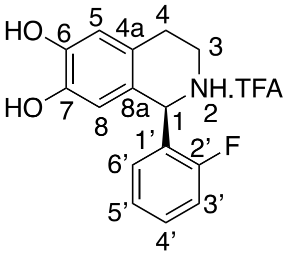

Purified by preparative HPLC method 1 and isolated as a white solid (11 mg, 31%). $[\alpha]_D^{25}$ -5.2 (*c* 0.23, MeOH); ν_{max}/ cm^{-1} (thin film): 3116, 2317, 1670, 1611, 1529; ^{1}H-NMR (700 MHz; CD_3OD) δ 7.52 (1H, dd, *J* = 5.3 Hz, 3'-H), 7.31 – 7.24 (2H, m, 3'-H and 4'-H), 7.19 (1H, d, *J* = 7.3 Hz, 6'-H), 6.70 (1H, s, 5-H), 6.20 (1H, s, 8-H), 5.87 (1H, s, 1-H), 3.46 (2H, t, *J* = 6.1 Hz, 3-H), 3.16 – 3.09 (1H, m, 4-*H*H), 3.02 (1H, dt, *J* = 17.2, 5.5 Hz, 4-H*H*); ^{13}C-NMR (176 MHz; CD_3OD) δ 162.4 (d, $^1J_{CF}$ = 248 Hz), 147.2, 146.0, 133.2 (d, J_{CF} = 8.4 Hz), 132.5 (d, J_{CF} = 2.1 Hz), 126.1 (d, J_{CF} = 3.5 Hz), 125.0 (d, $^2J_{CF}$ = 12 Hz), 124.4, 121.9, 117.1 (d, $^2J_{CF}$ = 21 Hz), 116.0, 114.6, 54.0, 41.4, 25.5; *m/z* [MS ES+] 260 ([M+H]$^+$, 100%); *m/z* [HRMS ES+] found [M+H]$^+$ 260.1081; $C_{15}H_{15}FNO_2$ requires 260.1081; Retention time (achiral analytical HPLC method 1): 4.7 min.

(1*S*)-1-(3-Fluorophenyl)-1,2,3,4-tetrahydroisoquinoline-6,7-diol (5g)

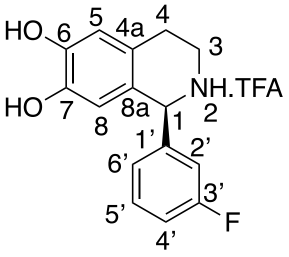

Purified by preparative HPLC method 1 and isolated as a white solid (11.0 mg, 31%); $[\alpha]_D^{25}$ -2.9 (*c* 0.41, MeOH); ν_{max}/ cm^{-1} (thin film): 3072, 2558, 2324, 1667, 1612, 1592, 1519; ^{1}H-NMR (700 MHz; CD_3OD) δ 7.51 (1H, dd, *J* = 5.8 Hz, 5'-H), 7.25 – 7.18 (2H, m, 4'-H and 6'-H), 7.13 (1H, d, *J* = 9.2 Hz, 2'-H), 6.69 (1H, s, 5-H), 6.20 (1H, s, 8-H), 5.62 (1H, s, 1-H), 3.52 – 3.37 (2H, m, 3-H), 3.18 – 3.09 (1H, m, 4-*H*H), 3.04 – 2.96 (1H, m, 4-H*H*).; ^{13}C-NMR (176 MHz; CD_3OD) δ 165.0 (d, $^1J_{CF}$ = 245 Hz), 147.3, 145.9, 140.5 (d, J_{CF} = 7.0 Hz), 132.1 (d, J_{CF} = 8.3 Hz), 126.8 (d, J_{CF} = 2.8 Hz), 124.3, 122.6, 117.7 (dd, $^2J_{CF}$ = 21.9, 4.8 Hz), 115.9, 115.1, 60.1, 41.4, 25.5; *m/z* [MS ES+] 260 ([M+H]$^+$, 100%); *m/z* [HRMS ES+] found [M+H]$^+$ 260.1081; $C_{15}H_{15}FNO_2$ requires 260.1081; Retention time (achiral analytical HPLC method 1): 5.0 min.

(1*S*)-1-(4-Fluorophenyl)-1,2,3,4-tetrahydroisoquinoline-6,7-diol (5h)

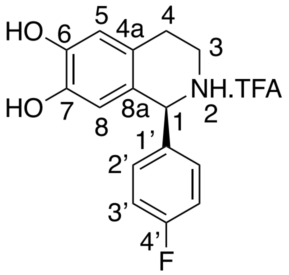

Purified by preparative HPLC method 1 and isolated as a white solid (10 mg, 29%). $[\alpha]_D^{25}$ -5.4 (*c* 0.46, MeOH); ν_{max}/ cm^{-1} (thin film): 3112, 2819, 1668, 1606, 1560, 1532, 1508; ^{1}H-NMR (700 MHz; CD_3OD) δ 7.43 – 7.37 (2H, m, 2'-H), 7.25 – 7.18 (2H, m, 3'-H), 6.69 (1H, s, 5-H), 6.19 (1H, s, 8-H), 5.61 (1H, s, 1-H), 3.49 – 3.44 (1H, m, 3-*H*H), 3.44 – 3.28 (1H, m, 3-H*H*), 3.16 – 3.08 (1H, m, 4-*H*H), 3.02 (1H, dt, *J* = 17.2, 5.7 Hz, 4-H*H*); ^{13}C-NMR (176 MHz; CD_3OD) δ 164.7 (d, $^1J_{CF}$ = 251 Hz), 147.2, 145.9, 134.1 (d, J_{CF} = 3.1 Hz), 133.1 (d, J_{CF} = 8.5 Hz), 124.3, 122.9, 117.0 (d, $^2J_{CF}$ = 23 Hz), 115.7, 115.1, 60.0, 41.2, 25.5; *m/z* [MS ES+] 260 ([M+H]$^+$, 100%); *m/z* [HRMS ES+] found [M+H]$^+$ 260.1081; $C_{15}H_{15}FNO_2$ requires 260.1081; Retention time (achiral analytical HPLC method 1): 5.1 min.

(1*S*)-1-(2-Chlorophenyl)-1,2,3,4-tetrahydroisoquinoline-6,7-diol (5i)

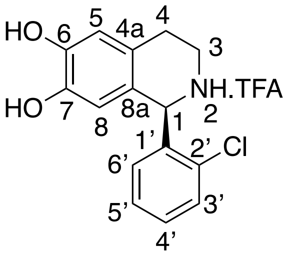

Purified by preparative HPLC method 1 and isolated as a white solid (13 mg, 35%). $[\alpha]_D^{25}$ 2.0 (*c* 0.12, MeOH); ν_{max}/ cm^{-1} (thin film): 2995, 2816, 2580, 1663, 1609, 1528; ^{1}H-NMR (700 MHz; CD_3OD) δ 7.61 (1H, dd, *J* = 8.1, 1.1 Hz, 3'-H), 7.48 (1H, td, *J* = 7.7, 1.6 Hz), 7.39 (1H, td, *J* = 7.7, 1.1 Hz, 5'-H or 4'-H), 7.19 (1H, dd, *J* = 7.7, 1.6 Hz), 6.70 (1H, s, 5-H), 6.14 (1H, s, 8-H), 6.04 (1H, s, 1-H), 3.50 – 3.43 (2H, m, 3-H), 3.11 (1H, dt, *J* = 17.3, 6.4 Hz, 4-*H*H), 3.05 – 3.00 (1H, *J* = 17.3, 6.2 Hz, 4-H*H*); ^{13}C-NMR (176 MHz; CD_3OD) δ 147.3, 146.2, 135.8, 135.6, 132.6, 132.5, 131.3, 129.0, 124.5, 122.1, 115.9, 114.7, 56.9, 41.2, 25.5; *m/z* [MS ES+] 276 ([M+H]$^+$, 100%); *m/z* [HRMS ES+] found [M+H]$^+$ 276.0786; $C_{15}H_{15}ClNO_2$ requires 276.0786; Retention time (achiral analytical HPLC method 1): 5.1 min.

(1*S*)-1-(3-Chlorophenyl)-1,2,3,4-tetrahydroisoquinoline-6,7-diol (5j)

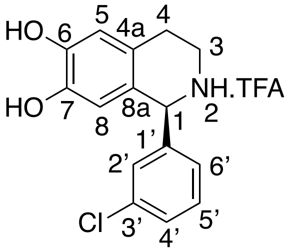

Purified by preparative HPLC method 1 and isolated as a white solid (5.2 mg, 14%). $[\alpha]_D^{25}$ 1.5 (*c* 0.015, MeOH); ν_{max}/ cm^{-1} (thin film): 3028, 2820, 2343, 2228, 1664, 1604, 1528; ^{1}H-NMR (700 MHz; CD_3OD) δ 7.52 – 7.45 (2H, m, 4'-H and 5'-H), 7.41 (1H, s, 2'-H), 7.30 (1H, d, *J* = 7.7 Hz, 6'-H), 6.69 (1H, s, 8-H), 6.19 (1H, s, 5-H), 5.60 (1H, s, 1-H), 3.51 – 3.38 (2H, m, 3-H), 3.17 – 3.10 (1H, m, 4-*H*H), 3.01 (1H, dt, *J* = 17.2, 5.5 Hz, 4-H*H*); ^{13}C-NMR (176 MHz; CD_3OD) δ 147.3, 146.0, 140.2, 136.1, 131.8, 130.9, 130.8, 129.3, 124.3, 122.4, 115.9, 115.0, 60.1, 41.4, 25.5; *m/z* [MS ES+] 276 ([M+H]$^+$, 100%); *m/z* [HRMS ES+] found [M+H]$^+$ 276.0786; $C_{15}H_{15}ClNO_2$ requires 276.0786; Retention time (achiral analytical HPLC method 1): 5.4 min.

(1*S*)-1-(4-Chlorophenyl)-1,2,3,4-tetrahydroisoquinoline-6,7-diol (5k)

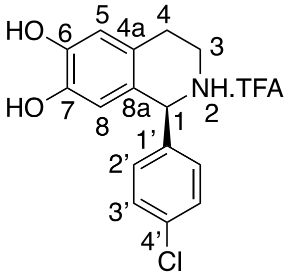

Purified by preparative HPLC method 1 and isolated as a white solid (6.9 mg, 19%). $[\alpha]_D^{25}$ 1.1 (*c* 0.0029, MeOH); ν_{max}/ cm^{-1} (thin film): 3166, 3102, 2772, 2634, 2556, 1800, 1670, 1610, 1589, 1535; ^{1}H-NMR (700 MHz; CD_3OD) δ 7.49 (2H, d, *J* = 8.5 Hz, 3'-H), 7.35 (2H, d, *J* = 8.5 Hz, 2'-H), 6.68 (1H, s, 5-H), 6.18 (1H, s, 8-H), 5.59 (1H, s, 1-H), 3.49 – 3.37 (2H, m, 3-H), 2.16 – 3.01 (1H, m, 4-*H*H), 3.00 (1H, dt, *J* = 17.2, 5.5 Hz, 4-H*H*); ^{13}C-NMR (176 MHz; CD_3OD) δ 147.2, 146.0, 136.8, 136.8, 132.5, 130.3, 124.3, 122.7, 115.9, 115.1, 60.0, 41.2, 25.5; *m/z* [MS ES+] 276 ([M+H]$^+$, 100%); *m/z* [HRMS ES+] found [M+H]$^+$ 276.0786; $C_{15}H_{15}ClNO_2$ requires 276.0786; retention time (achiral analytical HPLC method 1): 4.8 min.

(1*S*)-1-Cyclohexyl-6-methoxy-1,2,3,4-tetrahydroisoquinolin-7-ol (C6-OMe-7a) generated from using *Mx*SafC as the methyltransferase

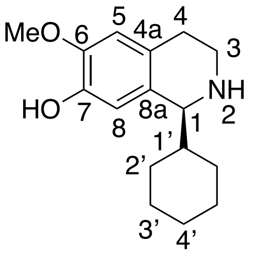

Purified by preparative HPLC method 2. $[\alpha]_D^{25}$ 1.1 (*c* 0.022, MeOH); ν_{max}/ cm^{-1} (thin film): 2930, 2855, 1670, 1614, 1516; ^{1}H-NMR (700 MHz; CD_3OD) δ 6.77 (1H, s, 5-H), 6.68 (1H, s, 8-H), 4.28 (1H, d, *J* = 5.2 Hz, 1-H), 3.84 (1H, s, OC*H*3), 3.56 – 3.51 (1H, m, 3-H), 3.30 – 3.26 (1H, m, 3-H), 3.06 – 2.99 (1H, m, 4-*H*H), 2.93 (1H, dt, *J* = 16.8, 5.3 Hz, 4-H*H*), 2.08 – 2.02 (1H, m, 1'-H), 1.89 – 1.83 (1H, m), 1.83 – 1.71 (3H, m), 1.59 – 1.53 (1H, m), 1.42 – 1.34 (1H, m), 1.34 – 1.26 (1H, m), 1.22 (2H, qd, *J* = 12.6, 3.4 Hz); ^{13}C-NMR (176 MHz; CD_3OD) δ 149.0, 146.8, 124.3, 123.0, 114.1, 112.6, 61.3, 56.3, 41.9, 41.5, 30.9, 27.6, 27.2, 27.0, 25.7; *m/z* [MS ES+] 262 ([M+H]$^+$, 100%); *m/z* [HRMS ES+] found [M+H]$^+$ 262.1802; $C_{16}H_{23}NO_2$ requires 262.1802; retention time (achiral analytical HPLC method 1): 6.2 min.

(1*S*)-6-Methoxy-1-phenyl-1,2,3,4-tetrahydroisoquinolin-7-ol (C6-OMe-7b) generated from using *Mx*SafC as the methyltransferase

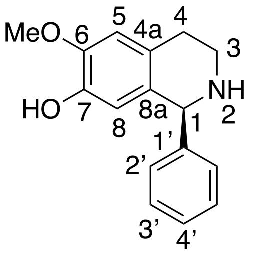

Purified by preparative HPLC method 2. $[\alpha]_D^{25}$ 1.35 (*c* 0.200, MeOH)**;** ν_{max}/ cm^{-1} (thin film): 2932, 2855, 2801, 2582, 1664, 1514; ^{1}H-NMR (700 MHz; CD_3OD) δ 7.50 – 7.45 (3H, m, 3'-H and 4'-H), 7.38 – 7.32 (2H, m, 2'-H), 6.85 (1H, s, 5-H), 6.21 (1H, s, 8-H), 5.60 (1H, s, 1-H), 3.87 (3H, s, OC*H*3), 3.52 – 3.47 (1H, m, 3-H), 3.47 – 3.41 (1H, m, 3-H), 3.23 - 3.17 (1H, m, 4-*H*H), 3.07 (1H, dt, *J* = 17.2, 5.5 Hz, 4-H*H*); ^{13}C-NMR (176 MHz; CD_3OD) δ 149.5, 146.9, 137.8, 130.9, 130.8, 130.3, 124.4, 124.2, 115.1, 112.3, 60.7, 56.3, 41.2, 25.7; *m/z* [MS ES+] 256 ([M+H]$^+$, 100%)); *m/z* [HRMS ES+] found [M+H]$^+$ 256.1332; $C_{16}H_{18}NO_2$ requires 256.1332; retention time (achiral analytical HPLC method 1): 5.7 min.

(1*S*)-6-Methoxy-1-phenyl-1,2,3,4-tetrahydroisoquinolin-7-ol (C6-OMe-7b) generated using *Rn*COMT as the methyltransferase. 87:13 ratio of C6-OMe-**7b**:C7-OMe-**7b** was generated.

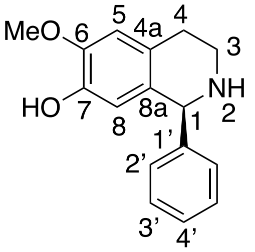

Purified by preparative HPLC method 2. ν_{max}/ cm^{-1} (thin film): 2932, 2855, 2801, 2582, 1664, 1514; ^{1}H-NMR (700 MHz; CD_3OD) δ 7.51 – 7.45 (3H, m, 3'-H and 4'-H), 7.38 – 7.33 (2H, m, 2'-H), 6.84 (0.9H, s, 5-H), 6.74 (0.1H, s, 5-H), 6.31 (0.1H, s, 8-H), 6.21 (0.9H, s, 8-H), 5.66 (0.1H, s, 1-H), 5.60 (0.9H, s, 1-H), 3.87 (2.7H, s, OC*H*$_3$), 3.62 (0.3H, s, OC*H*$_3$), 3.53 – 3.39 (2H, m, 3-H), 3.24 – 3.01 (2H, m, 4-H); ^{13}C-NMR (176 MHz; CD_3OD) δ 149.5, 146.9, 137.8, 130.9, 130.8, 130.3, 124.4, 124.2, 115.1, 112.3, 60.7, 56.3, 41.2, 25.7; *m/z* [MS ES+] 256 ([M+H]$^+$, 100%); *m/z* [HRMS ES+] found [M+H]$^+$ 256.1332; $C_{16}H_{18}NO_2$ requires 256.1332; retention time (achiral analytical HPLC method 1): 5.7 min.

(1*S*)-1-Cyclohexyl-7-methoxy-1,2,3,4-tetrahydroisoquinolin-6-ol

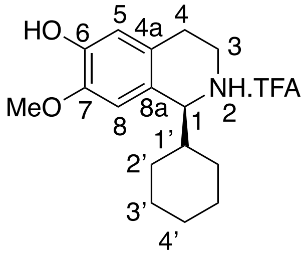

Synthesised from NCS mediated reaction between 4-methoxytyramine hydrochloride and **4a**. Purified by preparative HPLC method 2 and isolated as a white solid (8.4 mg, 32%). $[\alpha]_D^{26}$ -1.1 (*c* 0.29, MeOH)**;** ν_{max}/ cm^{-1} (thin film): 2971, 2853, 2165, 2033, 1677, 1517; ^{1}H-NMR (700 MHz, CD_3OD) δ 6.76 (1H, s, 8-H), 6.65 (1H, s, 5-H), 4.31 (1H, d, *J* = 5.6 Hz, C-1), 3.83 (3H, s, OC*H*$_3$), 3.53 (1H, dt, *J* = 12.3, 5.6 Hz, 3-*H*H), 3.28 – 3.22 (1H, m, 3-H*H*), 3.01 – 2.95 (1H, m, 4-*H*H), 2.88 (1H, dt, *J* = 16.9, 5.3 Hz, 4-H*H*), 2.12 – 2.06 (1H, m, 1'-H), 1.89 – 1.84 (1H, m), 1.83 – 1.71 (3H, m), 1.59 – 1.54 (1H, m), 1.43 – 1.35 (1H, qt, *J* = 12.8, 3.6 Hz), 1.35 – 1.26 (1H, m), 1.26 – 1.18 (1H, m), 1.10 (1H, qd, *J* = 12.6, 3.6 Hz); ^{13}C-NMR (176 MHz, CD_3OD) δ 148.3, 147.7, 125.9, 122.6, 116.2, 111.0, 61.5, 56.5, 41.9, 41.2, 30.9, 27.8, 27.2, 27.0, 26.9, 25.5; *m/z* [MS ES+] 262 ([M+H]$^+$, 100%); *m/z* [HRMS ES+] found [M+H]$^+$ 162.1799; $C_{16}H_{23}NO_2$ requires 262.1802.

(1*S*)-7-Methoxy-1-phenyl-1,2,3,4-tetrahydroisoquinolin-6-ol

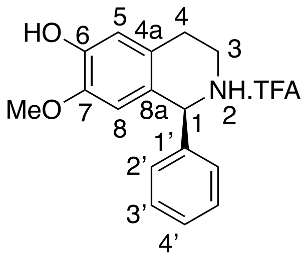

Synthesised from NCS mediated reaction between 4-methoxytyramine hydrochloride and **4b**. Purified by preparative HPLC method 2 and isolated as white solid (12 mg, 24%). $[\alpha]_D^{26}$ -2.0 (*c* 0.35, MeOH); ν_{max}/ cm^{-1} (thin film): 2925, 2853, 1631, 1515; ^{1}H-NMR (700 MHz, CD_3OD) δ 7.49 – 7.47 (3H, m, 2'-H and 4'-H), 7.38 – 7.35 (2H, m, 3'-H), 6.73 (1H, s, 5-H), 6.31 (1H, s, 8-H), 5.67 (1H, s, 1-H), 3.61 (3H, s, OC*H*$_3$), 3.49 – 3.39 (2H, m, 3-H), 3.17 – 3.12 (1H, m, 4-*H*H), 3.07 – 3.01 (1H, m, 4-*H*H); ^{13}C-NMR (176 MHz, CD_3OD) δ 148.5, 148.2, 137.9, 130.9, 130.9, 130.3, 125.8, 122.8, 115.9, 111.7, 60.6, 56.2, 40.7, 25.5; *m/z* [MS ES+] 256 ([M+H]$^+$, 100%)); *m/z* [HRMS ES+] found [M+H]$^+$ 256.1333; $C_{16}H_{18}NO_2$ requires 256.1332.

Preparation of calibration curves

Calibration curves were prepared by serial of a 2.5 mM stock of the product in 20% MeCN in water, which was isolated by preparative-HPLC purification (methods given for each compound isolated for characterisation purposes). Samples were analysed by achiral analytical HPLC method 1. Absorbance is given in mAU, measured at 280 nm.

Methods to determine methyltransferase selectivities

Experiments were performed as described in the methods section using *rac*-**5a** and (*S*)-**5a** and also *rac*-**5b** and (*S*)-**5b** with *Rn*COMT and *Mx*SafC, with analysis by RP-HPLC and chiral HPLC. Reaction conversions and regioselectivities, and where lower conversions were observed, enantioselectivities were monitored. To aid determination of the stereospecificity and regioselectivity, chemical standards for C7-OMe-**7a** and C7-OMe **7b** were synthesised using NCS (M97V), 4-methoxytyramine and the corresponding aldehydes (Supplementary Methods).

Methylation of 5a by *Rn*COMT (Supplementary Figures 27a and 28a):
Rac-**5a** was fully converted to **7a** and a 50:50 product ratio for C6:C7 methylation was observed. In addition, using the racemic standard of C7-OMe-**7a** synthesised showed that for this regioisomer a 84:16 *S*:*R* product was generated so *Rn*COMT is not regio- or enantioselective towards *rac*-**5a**. With (*S*)-**5a**, only C6-methylation was observed.
Methylation of 5a by *Mx*SafC (Supplementary Figures 27b and 28b):
Rac-**5a** and (*S*)-**5a** both gave only C6-OMe-**7a**. Conversions were increased when using *rac*-**5a** compared to (*S*)-**5a** as the starting material (46% vs. 33%). For the *rac*-**5a** reaction with *Mx*SafC, analysis of the remaining **5a** showed that only (*R*)-**5a** remained, so *Mx*SafC is selective towards (*S*)-**5a**.
Methylation of 5b by *Rn*COMT (Supplementary Figures 27c and 28c):
Rac-**5b** was was fully converted to **7b** and a 50:50 product ratio for C6:C7 methylation was observed. With (*S*)-**5b**, a 87:13 ratio for C6:C7-methylation was observed highlighting its generally poorer selectivity.
Methylation of 5b by *Mx*SafC (Supplementary Figures 27d and 28d):
Rac-**5b** and (*S*)-**5a** both gave only C6-OMe-**7b**. Conversions were similar when using *rac*-**5b** and (S)-**5b** (50% vs. 46%). For the *rac*-**5b** reaction with *Mx*SafC, analysis of the remaining **5b** showed that only (*R*)-**5b** remained, so *Mx*SafC is selective towards (*S*)-**5b**.

Synthesis of (*S*)-6,7-dimethoxy-1-phenyl-1,2,3,4-tetrahydroisoquinoline

(1*S*)-1-Phenyl-1,2,3,4-tetrahydroisoquinoline-6,7-diol (5b)

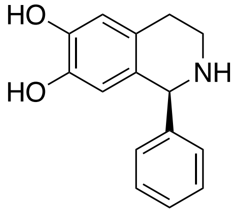

A solution of dopamine·HCl (37.9 mg, 0.2 mmol, 1 e.q.) and sodium ascorbate (39.6 mg, 0.2 mmol) in HEPES buffer (50 mM, pH 7.5, 18 mL) was prepared. To this, a solution of benzaldehyde (0.4 mmol, 40.8 μL, 2 e.q.) in MeCN (2 mL) was added and 4 mg of *Tf*NCS-M97V (10 mg mL^{-1} in 20 mM Tris, 50 mM NaCl, pH 7.5). The reaction mixture was stirred for 24 h at 37 °C. The reaction mixture was extracted into ethyl acetate (3 x 20 mL), dried and concentrated. The residue was resuspended in 1 M HCl (20 mL) and washed with dimethyl carbonate (1 x 5 mL). The aqueous layer was dried under reduced pressure. The product was resuspended in sat. aq. $NaHCO_3$, extracted into EtOAc (3 x 20 mL), dried (Na_2SO_4) and concentrated under reduced pressure to give the crude product as a yellow oil (30.0 mg, 62%) and the identity confirmed by ^{1}H-NMR analysis (matching that of purified **5b**).

Synthesis of *tert*-butyl 6,7-dihydroxy-1-phenyl-3,4-dihydroisoquinoline-2(1*H*)-carboxylate

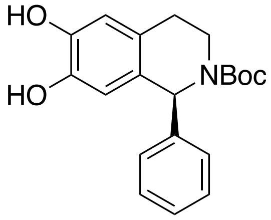

(1*S*)-1-Phenyl-1,2,3,4-tetrahydroisoquinoline-6,7-diol (30.0 mg, 0.124 mmol, 1 e.q.) was resuspended in THF (4 mL) and sat. aq. $NaHCO_3$ (4 mL). Boc anhydride (54.2 mg, 0.248 mmol, 2 e.q.) was added and the reaction mixture stirred at rt (24 h). The reaction mixture was extracted into EtOAc (3 x 15 mL), dried (Na_2SO_4) and concentrated under reduced pressure. The crude product was taken through to the next step without further purification.

Synthesis of *tert*-butyl (*S*)-6,7-dimethoxy-1-phenyl-3,4-dihydroisoquinoline-2(1*H*)-carboxylate[8]

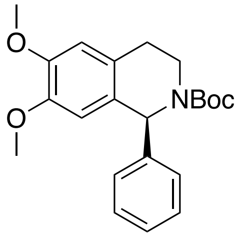

A solution of *tert*-butyl 6,7-dihydroxy-1-phenyl-3,4-dihydroisoquinoline-2(1*H*)-carboxylate (28 mg, 0.082 mmol) and base (potassium carbonate or caesium carbonate) (0.246 mmol) in methanol (5 mL) was prepared. Iodomethane (102 μL, 1.64 mmol) was added and the reaction mixture stirred for 24 h at

room temperature. Solvents were removed under reduced pressure and the resulting residue azeotroped with methanol (3 x 10 mL). The product was partially purified by silica chromatography (100% dichloromethane) to give the product as a yellow oil (6.5 mg, 21%). The crude product was taken through to the next step without further purification.

Synthesis of (*S*)-6,7-dimethoxy-1-phenyl-1,2,3,4-tetrahydroisoquinoline

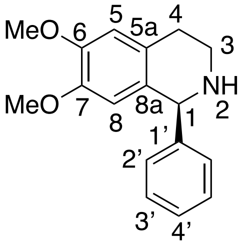

A solution of *tert*-butyl (*S*)-6,7-dimethoxy-1-phenyl-3,4-dihydroisoquinoline-2(1*H*)-carboxylate (6.5 mg, 0.018 mmol) in dichloromethane (3.2 mL) and trifluoroacetic acid (0.8 mL) was prepared and stirred at room temperature for 5 min. Solvents were removed under reduced pressure, then resuspended in 50% MeOH in water and concentrated. The resulting residue was purified by semi-preparative HPLC or preparative HPLC method 3 to give the desired product in 6% or 20% e.e. as determined by chiral HPLC methods 3 and 4, dependant on which base was used for the reaction (6% for potassium carbonate and 20% for caesium carbonate, Supplementary Figures 21 and 22). ^{1}H-NMR (700 MHz; CD_3OD) δ 7.50 – 7.47 (3H, m, Ar*H*), 7.36 – 7.34 (2H, m, Ar*H*), 6.89 (1H, s, 5-H), 6.34 (1H, s, 8-H), 5.69 (1H, s, 1-H), 3.85 (3H, s, OCH_3), 3.59 (3H, s, OCH_3), 3.51 – 3.41 (2H, m, 3-H), 3.25 – 3.18 (1H, m, 4-*H*H), 3.14 – 3.08 (1H, m, 4-H*H*); ^{13}C-HMR (176 MHz; CD_3OD) δ 150.9, 149.8, 137.6, 131.0, 130.9, 130.3, 125.8, 124.0, 112.6, 111.9, 60.5, 56.4, 56.3, 40.6, 25.7; *m/z* [ES+] 271 ([M+H]$^+$, 100%).

Synthesis of reaction intermediate mimic, 6

***N*-(3,4-Bis(benzyloxy)phenethyl) benzamide**

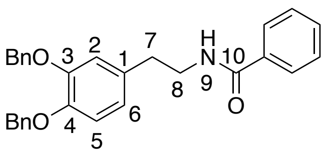

A solution of 2-(3,4-bis(benzyloxy)phenyl)ethan-1-amine (317 mg, 0.95 mmol, 1 e.q.), benzoic acid (128 mg, 1.04 mmol, 1.1 e.q.) and HATU (362 mg, 0.95 mmol, 1 e.q.) in DMF (5 mL) was prepared in anhydrous conditions and cooled to 0 °C. *N,N*-diisopropylethylamine (332 μL, 1.9 mmol, 2 e.q.) was added dropwise and the reaction mixture allowed to room temperature. The reaction mixture was stirred for 18 h, then solvents removed under reduced pressure. The resulting residue was resuspended in EtOAc (15 mL), washed with 1 M HCl (2 x 15 mL), $NaHCO_3$ (2 x 15 mL) and brine (2 x 15 mL), dried and concentrated under reduced pressure. The resulting residue was purified by column chromatography 5 – 25% ethyl acetate in petroleum ether, to give the pure product as a white solid (287 mg, 0.66 mmol, 69%). ν_{max}/ cm^{-1} (thin film): 3309, 3060, 3028, 2927, 2858, 1637, 1601, 1577, 1534, 1510; ^{1}H-NMR (700 MHz; $CDCl_3$) δ 7.68-7.64 (2H, m, COC(C*H*)$_2$), 7.50-7.26 (13H, m, Ar*H*), 6.90 (1H, m, 5-H), 6.83 (1H, m, 2-H), 6.74 (1H, m, 6-H), 6.07 (1H, m, N*H*), 5.14 (2H, s, PhC*H*$_2$O), 5.11 (2H, s, PhC*H*$_2$O), 3.65 (2H, q, *J* = 6.8 Hz, C*H*$_2$NH), 2.83 (2H, t, *J* = 6.8 Hz, C*H*$_2$CHNH); ^{13}C-NMR (176 MHz; $CDCl_3$) δ 167.5, 149.3, 147.9, 137.5, 137.3, 134.8, 132.4, 131.6, 128.7, 128.6, 128.0, 127.9, 127.5, 127.5, 126.9, 121.8, 115.9, 115.7, 71.6, 71.5, 41.2, 35.2; *m/z* [MS ES+] 438 ([M+H]$^+$, 100%); *m/z* [HRMS ES+] found [M+H]$^+$ 438.2050; $C_{29}H_{27}NO_2$ requires 438.2069.

***N*-Benzyl-2-(3,4-bis(benzyloxy)phenyl)ethan-1-amine**

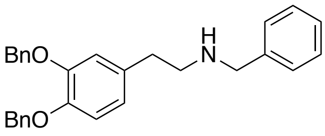

A solution of *N*-(3,4-bis(benzyloxy)phenethyl) benzamide (250 mg, 0.57 mmol) in THF (20 mL) was prepared under anhydrous conditions. Boron trifluoride diethyl etherate (36 μL, 0.29 mmol) was added and the reaction stirred under reflux for 10 min. Boron dimethyl sulfide complex (2 M in THF, 855 μL, 1.71 mmol) was added and the reaction mixture stirred for a further 3 h. The mixture was then cooled to 0 °C, 1 M HCl_{aq} added (10 mL) and stirred for 1 h. The reaction was then warmed to room temperature and stirred overnight. The pH was adjusted to 13 by addition of 2 M NaOH, the solution extracted with dichloromethane (3 x 20 mL). The organic phase was then dried with Na_2SO_4 and concentrated under reduced pressure to give the crude product as a grey oil which was taken through to the next step without further purification.

4-(2-(benzylamino)ethyl)benzene-1,2-diol (6)

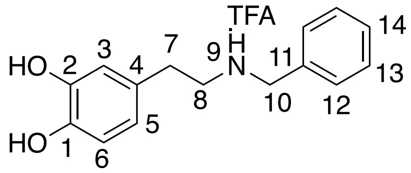

A solution of *N*-benzyl-2-(3,4-bis(benzyloxy)phenyl)ethan-1-amine (240 mg, 0.57 mmol) in MeOH (30 mL) and 37% HCl (6 mL) was heated under reflux for 18 h. Solvents were removed under reduced pressure and the resulting residue was purified by preparative HPLC (method 1), lyophilized and the pure product isolated as a white solid (57 mg, 29%). ν_{max}/ cm^{-1} (thin film): 3039, 1661, 1528; ^{1}H-NMR (700 MHz; D_2O) δ 7.47-7.37 (5H, m, 12-H,13-H,14-H), 6.84 (1H, d, J = 8.1 Hz, 6-H), 6.76 (1H, d, J = 2.2 Hz, 3-H), 6.68 (1H, dd, J = 8.1, 2.2 Hz, 5-H), 4.19 (2H, s, 10-H), 3.23 (2H, t, J = 7.6 Hz, 8-H), 2.86 (2H, t, J = 7.6 Hz, 7-H); ^{13}C-NMR (176 MHz; D_2O) δ 144.8, 143.6, 131.1, 130.3, 130.2, 129.8, 129.6, 121.7, 117.1, 117.0, 50.8, 48.5, 31.4; *m/z* [MS ES+] 244 ($[M+H]^+$, 100%); *m/z* [HRMS ES+] found $[M+H]^+$ 244.1332; $C_{15}H_{17}NO_2$ requires 244.1338.

Synthesis of 4-methoxytyramine hydrochloride

O-Benzylisovanillin

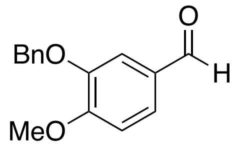

Under anhydrous conditions, a solution of isovanillin (5.00 g, 33.0 mmol) and potassium carbonate (3.25 g, 23.5 mmol) in DMF (75 mL) was prepared. Benzyl bromide (8.23 mL, 69.3 mmol) was added and the solution heated under reflux for 18 h. The solution was concentrated under reduced pressure, resuspended in diethyl ether (25 mL) and washed with 1 M NaOH (3 x 25 mL), brine (2 x 25 mL) and water (2 x 25mL). The organic phase was dried under reduced pressure and the resulting residue was washed with *n*-hexane (200 mL) and dried under vacuum to give *O*-benzylvanillin as a white solid (4.5 g, 57%).[9] m.p. = 61 – 62 °C (CH_2Cl_2, lit: 61 – 63 °C)[9]; ^{1}H-NMR (600 MHz; $CDCl_3$) δ 9.80 (1H, s, CO*H*), 7.46 (4H, m, Ar*H*), 7.38 (2H, t, *J* = 7.6 Hz, Ar*H*), 7.32 (1H, t, *J* = 7.6 Hz, Ar*H*), 6.98 (1H, d, *J* = 8.6 Hz, Ar*H*), 5.17 (2H, s, C*H*$_2$Ph), 3.93 (3H, s, OC*H*$_3$); ^{13}C-NMR (151 MHz; $CDCl_3$) δ 190.9, 155.2, 148.8, 136.5, 130.1, 128.8, 128.3, 127.6, 127.0, 111.6, 111.0, 71.0, 56.3; *m/z* [MS ES+] 243 ([M+H]$^+$, 100%).

Trans-3-benzyloxy-4-methoxy-β-nitrostyrene

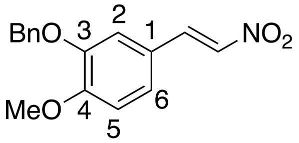

A solution of *O*-benzylvanillin (1.00 g, 4.10 mmol) in acetic acid (12.5 mL), nitromethane (1.40 mL, 25.8 mmol) and ammonium acetate (1.60 g, 20.8 mmol) was prepared and heated under reflux for 4 h, rt for 18 h then reflux for 6 h. The reaction was quenched by addition of water (20 mL) followed by extraction into diethyl ether (3 x 30 mL). The organic phases were combined, dried with anhydrous $MgSO_4$ and concentrated under reduced pressure to give *trans*-3-benzyloxy-4-methoxy-β-nitrostyrene as a yellow solid (1.05 g, 90%).[9] m.p. = 126 - 128 °C (EtOH, lit: 126 – 128 °C)[9]; ^{1}H-NMR (600 MHz; $CDCl_3$) δ 7.90 (1H, d, *J* = 13.6 Hz, C*H*NO$_2$), 7.47 – 7.30 (5H, m, ArC*H* and Ar*H*), 7.33 (1H, m, Ar*H*), 7.17 (1H, dd, *J* = 8.3, 2.1 Hz, 6-H), 7.03 (1H, d, *J* = 2.1 Hz, 2-H), 6.93 (1H, d, *J* = 8.3 Hz, 5-H), 5.18 (2H, s, PhC*H*$_2$), 3.95 (3H, s, OC*H*$_3$); ^{13}C-NMR (151 MHz; $CDCl_3$) δ 153.6, 148.7, 139.5, 136.4, 135.2, 128.9, 128.8, 128.4, 127.5, 125.0, 122.8, 113.2, 111.8, 71.3, 56.3; *m/z* [MS ES+] 286 ([M+H]$^+$, 100%).

(3-Benzyloxy-4-methoxyphenyl)ethylamine

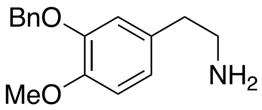

Under anhydrous conditions, a solution of *trans*-3-benzyloxy-4-methoxy-β-nitrostyrene (2.80 g, 9.81 mmol) in THF (50 mL) was prepared. The solution was cooled to 0 °C and $LiAlH_4$ (2M in THF, 2.66 mL, 4.0 mmol) was added dropwise. The reaction was heated under reflux for 4 h then cooled to 0 °C and quenched by addition of water (1.4 mL), 1 M NaOH (1.4 mL) and water (4.2 mL). The solution was allowed to warm to rt, filtered and concentrated under reduced pressure to give (3-benzyloxy-4-methoxyphenyl)ethylamine which was taken through to the next step without further purification.

4-Methoxytyramine hydrochloride

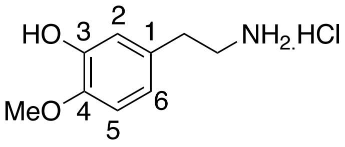

A solution of (3-benzyloxy-4-methoxyphenyl)ethylamine in methanol (20 mL) and 37% HCl (10 mL) was prepared and heated under reflux for 48 h. Solvents were removed under reduced pressure and the resulting residue resuspended in 1 M HCl and washed with ethyl acetate (3 x 30 mL). The aqueous layer was concentrated under reduced pressure, washed with acetone (100 mL) and dried further under reduced pressure to give crude 4-methoxytyramine as a brown oil (563 mg, 70%).[10] The compound was used directly for enzymatic reactions. ^{1}H-NMR (600 MHz; CD_3OD) δ 6.89 (1H, d, J = 8.2 Hz, 5-H), 6.73 (1H, d, J = 2.0 Hz, 2-H), 6.70 (1H, dd, J = 8.2, 2.0 Hz, 6-H), 3.83 (3H, s, OCH_3), 3.11 (2H, t, J = 7.5 Hz, CH_2NH_2), 2.83 (2H, t, J = 7.5 Hz, CH_2CH_2NH_2); ^{13}C-NMR (151 MHz; CD_3OD) δ 148.3, 148.0, 130.5, 120.9, 116.7, 113.1, 56.3, 42.1, 33.9; *m/z* [MS ES+] 166 ($[M+H]^+$, 100%).

Supplementary References

1. Ruff, B. M., Bräse, S. & O'Connor, S. E. Biocatalytic production of tetrahydroisoquinolines. *Tetrahedron Lett.* **53**, 1071–1074 (2012).
2. Emsley, P., Lohkamp, B., Scott, W. G. & Cowtan, K. Features and development of Coot . *Acta Crystallogr. Sect. D Biol. Crystallogr.* **66**, 486–501 (2010).
3. Roddan, R. *et al.* Acceptance and Kinetic Resolution of α-Methyl-Substituted Aldehydes by Norcoclaurine Synthases. *ACS Catal.* **9**, 9640–9649 (2019).
4. Lichman, B. R., Zhao, J., Hailes, H. C. & Ward, J. M. Enzyme catalysed Pictet-Spengler formation of chiral 1,1'-disubstituted- and spiro-tetrahydroisoquinolines. *Nat. Commun.* **8**, 14883 (2017).
5. Siegrist, J. *et al.* Regiocomplementary O-Methylation of Catechols by Using Three-Enzyme Cascades. *ChemBioChem* **16**, 2576–2579 (2015).
6. Mordhorst, S., Siegrist, J., Müller, M., Richter, M. & Andexer, J. N. Catalytic Alkylation Using a Cyclic S-Adenosylmethionine Regeneration System. *Angew. Chemie Int. Ed.* **56**, 4037–4041 (2017).
7. Pesnot, T., Gershater, M. C., Ward, J. M. & Hailes, H. C. Phosphate mediated biomimetic synthesis of tetrahydroisoquinoline alkaloids. *Chem. Commun.* **47**, 3242–3244 (2011).
8. Collins, J. L., Fujii, A., Roshandel, S., To, C. A. & Schramm, M. P. Calixarene-mediated liquid membrane transport of choline conjugates 3: The effect of handle variation on neurotransmitter transport. *Bioorganic Med. Chem. Lett.* **27**, 2953–2956 (2017).
9. Bermejo, A. *et al.* Syntheses and antitumor targeting G1 phase of the cell cycle of benzoyldihydroisoquinolines and related 1-substituted isoquinolines. *J. Med. Chem.* **45**, 5058–5068 (2002).
10. Lichman, B. R. *et al.* Structural Evidence for the Dopamine-First Mechanism of Norcoclaurine Synthase. *Biochemistry* **56**, 5274–5277 (2017).
